# Nile Red-Based Covalent Organic Framework as a Photocatalyst for C–H Bond Functionalization

**DOI:** 10.1021/acscatal.5c02173

**Published:** 2025-06-06

**Authors:** Marta Gordo-Lozano, Diego G. Matesanz, Marcos Martínez-Fernández, Pedro Almendros, Emiliano Martínez-Periñán, José L. Segura, Sara Cembellín

**Affiliations:** a Macromolecular and Heterocyclic Organic Materials Group, Organic Chemistry Department, Facultad de Ciencias Químicas, 16734 Universidad Complutense de Madrid, Madrid 28040, Spain; b Green Catalysis Laboratory, Organic Chemistry Department, Facultad de Ciencias Químicas, 16734 Universidad Complutense de Madrid, Madrid 28040, Spain; c Center for Innovation in Advanced Chemistry (ORFEO−CINQA), https://orfeocinqa.es/; d Instituto de Química Orgánica General, IQOG-CSIC, Consejo Superior de Investigaciones Científicas, Madrid 28006, Spain; e Sensors and Biosensors group, Departamento de Química Analítica y Análisis Instrumental, Facultad de Ciencias, and Institute for Advanced Research in Chemical Sciences (IAdChem), 16722 Universidad Autónoma de Madrid, Campus de Cantoblanco, Madrid 28049, Spain

**Keywords:** COF, Nile Red, photocatalysis, C−H functionalization, heterogeneous catalysis

## Abstract

The search for efficient photocatalysts based on covalent organic frameworks (COFs) is an area of increasing interest. However, the development of these heterogeneous photocatalysts is hindered by the symmetry restrictions of the linkers used to construct these materials. Herein, we report the straightforward synthesis of an imine-based 2D-COF, **NR**_**0.17**_ **-COF**, which incorporates a Nile Red (NR) unit via postmodification with a NR-alkyne scaffold. This framework exhibits remarkable photocatalytic activity across various photoredox-catalyzed C–H functionalization reactions, demonstrating the ability to directly functionalize prevalent bonds in organic molecules under mild conditions and with low-energy light. The **NR**_**0.17**_ **-COF** showcases notable versatility, effectively generating aryl, sulfur, and nitrogen radicals from different radical precursors while maintaining good functional group tolerance. Moreover, our heterogeneous photocatalyst outperforms traditional homogeneous systems by addressing critical challenges such as scalability and recyclability, allowing for a 10-fold increase in the reaction scale and enabling recovery and reuse up to six times. This advancement significantly enhances the potential of COF postsynthetic modification for practical applications in organic synthesis, which marks a substantial step forward in photocatalytic technology.

## Graphical abstract

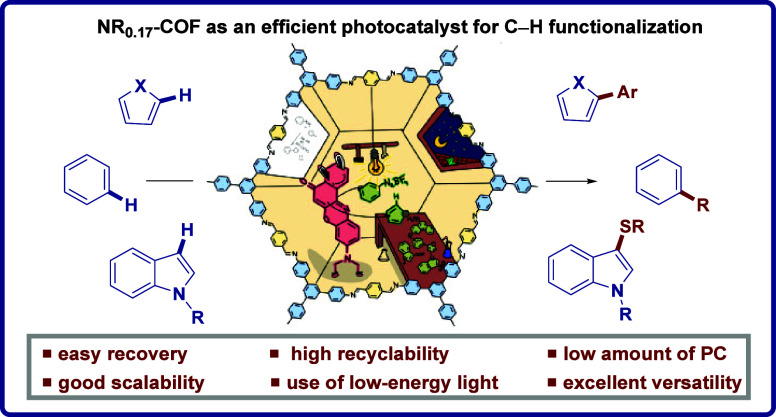

## 1 Introduction

Over the past few decades, society has increasingly acknowledged the pressing need to create cleaner and more sustainable production processes.^1^ Photocatalysis stands out as a viable solution, as it enables an environmentally conscious and sustainable approach to conducting chemical reactions under exceptionally mild conditions. In particular, visible light photocatalysis has attracted great attention in organic synthesis, avoiding the necessity of special equipment and reducing side reactions, often associated with high energy UV light protocols.^2^ Hence, numerous efficient, economical, and ecological transformations have been developed through this strategy, experiencing recently a considerable growth the reactions enabled with low-energy photons, which can bear potential biomedical applications.^3^

In the field of photocatalysis, a compound known as a photocatalyst has the ability to harness light energy, making it possible to initiate or accelerate reactions that would otherwise be unfeasible. During last years, numerous discrete molecular photocatalysts, including transition metal complexes and organic dyes, have been extensively investigated, showcasing remarkable efficacy across diverse synthetic protocols.^4^ Nonetheless, homogeneous photocatalysts come with inherent drawbacks, such as limited recovery, recyclability and often substantial costs, which hinder their practical application in industrial settings. Likewise, the challenges associated with scaling up these photocatalytic transformations should also be considered (Scheme 1a, left).^5^

**Scheme 1:**
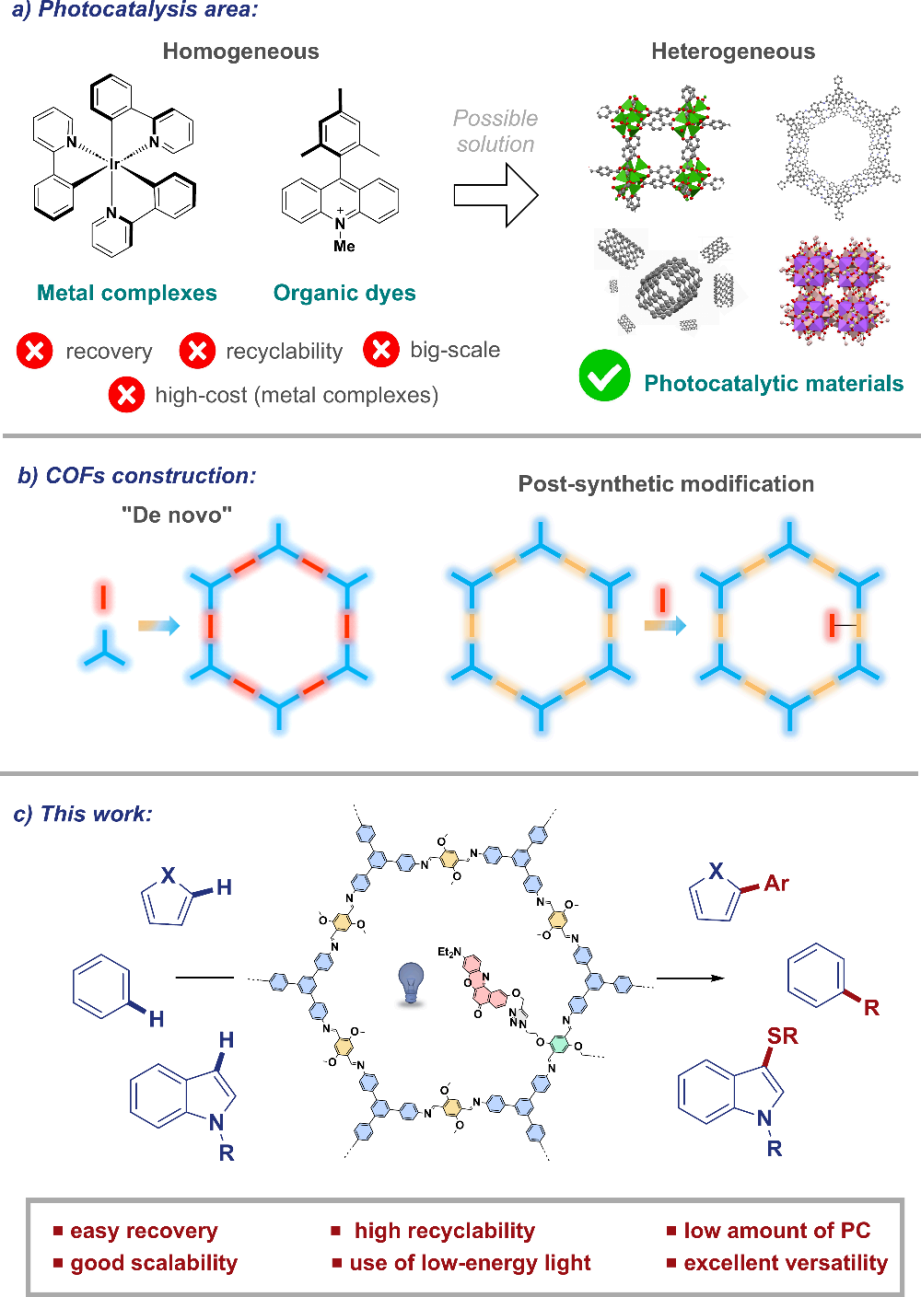
(a–c) Overview of This Work

Consequently, the cited inconveniences have propelled the surge in interest and recognition for heterogeneous photocatalysts, thanks to their simplified integration into industrial processes, coupled with advantageous attributes like prolonged lifespan and reusability.^6^ In this context, porous organic materials such as zeolites, silicas, carbon nanotubes, or MOFs, along with others, are gaining a prominent role due to their facile incorporation of photoactive fragments and their ability to construct highly conjugated, photocatalytic pristine materials (Scheme 1a, right).^7^ Among them, Covalent Organic Frameworks (COFs) have attracted high attention due to their predesignability and the ability to form crystalline and hierarchically organized porous structures.^8^ In fact, COFs have been employed as efficient photocatalysts to carry out diverse organic transformations,^9^ contaminant degradation (such as dyes or CO2)^10^ or energy-related conversions (e.g., hydrogen evolution).^11^ Moreover, their insoluble nature has put them in the spotlight for the heterogeneous operando in different applications such as adsorption, batteries, and other types of catalysis.^12^

However, most of these materials are produced through *de novo* synthesis, involving the reaction between the different linkers of the COFs to generate the final crystalline structure. This necessitates that the linkers satisfy specific symmetry requirements to construct the crystalline frameworks,^13^ which limits the number of photoactive fragments that can be incorporated into the extended network as most of them do not meet these criteria. In order to solve this problem, in recent years it has been emerged several new strategies, such as coordination, decoration, or pendant group reaction, that can include the photoactive unit in the already formed organic framework through postsynthetic modifications, yielding accessible active sites (Scheme 1b).^14^

Taking advantage of this postfunctionalization approach, we have described various COFs bearing different electroactive moieties.^15^ In particular, in 2022 we reported the synthesis of a Nile Red-based COF by using the copper­(I) catalyzed azide–alkyne cycloaddition (CuAAC) and explored its electrocatalytic applications.^16^ Nile Red (NR) is a notable neutral chromophore, primarily used for biological imaging, with absorption around 500 nm and emission around 600 nm, in nonpolar solvents. Additionally, it offers several sites for modifying its organic structure without losing the photophysical properties of the push–pull system.^17^ However, presumably due to the high cost (776€/g) it is rarely used as photocatalyst.^18,19^ Therefore, we envisioned that evaluating the photocatalytic potential of this type of material would be highly interesting, particularly by synthesizing a new Nile Red-based COF through a more efficient pathway. In this manner, we would obtain a photoactive material capable of absorbing at longer wavelengths, thereby addressing the primary disadvantage of Nile Red, as a smaller amount of photoactive molecule would be required per mmol of the heterogeneous photocatalyst and thus also per mmol of reaction product.

Herein, we report the efficient realization of this approach through the synthesis of **NR**_**0.17**_ **-COF**, with a molar loading of 6 × 10^–4^ mmol of NR unit per mg of host COF, achieved via a postmodification of the framework with a NR-alkyne (**NR-Alk**) scaffold prepared directly from commercially available materials. The photocatalytic activity of the material has proven to be remarkable in a variety of photoredox-catalyzed C–H functionalization reactions (Scheme 1c). Thus, it has demonstrated its ability to directly functionalize the most common bonds in organic molecules under mild conditions, even using low-energy light, while allowing the reactions to be scaled up without any loss of efficiency. Finally, due to its insoluble nature, this heterogeneous photocatalyst can be completely recovered and recycled over six cycles, overcoming many of the challenges associated with homogeneous photocatalysis.

## 2 Materials and Methods

### 2.1 Materials

Solvents and commercially available chemicals, such as 3-diethylaminophenol, 1,6-dihydroxy naphtol, propargyl bromide, etc., were obtained from Merck, VWR, Fisher Scientific, Scharlab, Alfa Aesar and Fluorochem, and used without further purification.

### 2.2 Material Characterization

Fourier transform infrared (FT-IR) spectroscopy was performed on a PerkinElmer 100 spectrophotometer equipped with a PIKE Technologies MlRacle Single Reflection Horizontal ATR Accessory and on a Bruker TENSOR 27 instrument on a diamond plate. Mass spectroscopy (MS) was performed on a Bruker Model HCT Ultra Ion Trap Mass Spectrometer (Mass rage:50–6000 amu) coupled to HPLC with ESI, APCI and NS interfaces. Powder X-ray diffraction (PXRD) measurements were carried out with an X’PERT MPD with conventional Bragg–Brentano geometry using monochromatic Cu Kα1 radiation (λ = 1.5406 Å) in the 2θ = 1.8°-40° range. For N_2_ sorption isotherms, N_2_ (77 K) adsorption–desorption measurements were carried out on a Micromeritics Tristar 3000 and samples were previously activated for 4 h under high vacuum high vacuum (<10^–7^ bar) at 120 °C. UV–vis absorption spectra were recorded on a Varian Cary 50 Scan UV–vis Spectrophotometer, and emission spectra were recorded on a Jasco FP-6300 Spectrofluorometer. Scanning Electron Microscopy (SEM) images were acquired on a JEOL JSM7600F microscope. Transmission Electron Microscopy (TEM) images were recorded on a JEOL JEM 2100 microscope.

### 2.3 Electrochemical Measurements

Electroche-mical measurements were performed with a potentiostat Autolab PGSTAT302N (EcoChemie, NL) using the software package NOVA 2.16. A three-electrode setup using a homemade single-compartment electrochemical cell was employed. Glassy carbon (GC) from CH Instruments were used as working electrodes and Pt wire as counter electrode. Specific calomel electrode (1 M LiCl for organic media from Radiometer Analytical) was used as the reference electrode. Electrochemical measurements have been carried out in 0.1 M TBAP (tetrabutylammonium perchlorate)/acetonitrile solution previously deoxygenated using argon.

### 2.4 Phoyocatalytic Reactions and Product Analysis

Photocatalytic reactions were performed in a homemade photoreactor with LEDs of different wavelengths and 18 W of intensity (measured temperature with fan on = 24 °C; for more details, see the SI). Analytical thin layer chromatography (TLC) was performed on silica gel 60 F254 aluminum plates (Merck) and they were visualized by exposure to short wave ultraviolet light (254 nm, 366 nm) and/or by staining. Flash chromatography was performed on Scharlab silica gel (40–63 mesh) by standard techniques using appropriate mixtures of hexane and ethyl acetate with compressed air. ^1^H- and ^13^C NMR spectra were recorded at room temperature on a Bruker AVIII HD 300 MHz BACS-60, Bruker Neo 300 MHz, Bruker AVIII HD-WB 400 MHz and Bruker AVIII 700 MHz in deuterated solvents as indicated or in the solid state.

## 3 Results and Discussion

### 3.1 Improved Synthesis of the Photoactive Fragment NR-Alk

In contrast to the preparation of our previous Nile Red-based COF,^16^ we now opted to introduce the alkyne unit, necessary for further functionalization, directly into the photoactive fragment, reducing the number of steps in the protocol. Hence, **NR-Alk** was synthesized by the reaction of commercially available *N,N’*-diethyl-3-aminophenol (**1**) with NaNO_2_, producing 5-diethylamino-2-nitrosophenol (**2**). Treatment of **2** with 1,6-dihydroxynaphthol (**3**) formed Nile Red functionalized with a hydroxyl anchor point (**NR–OH**). Finally, the alcohol functional group was transformed by Williamson etherification using an excess of propargyl bromide (**4**), yielding **NR-Alk** in a more straightforward procedure (Scheme 2).

**Scheme 2:**
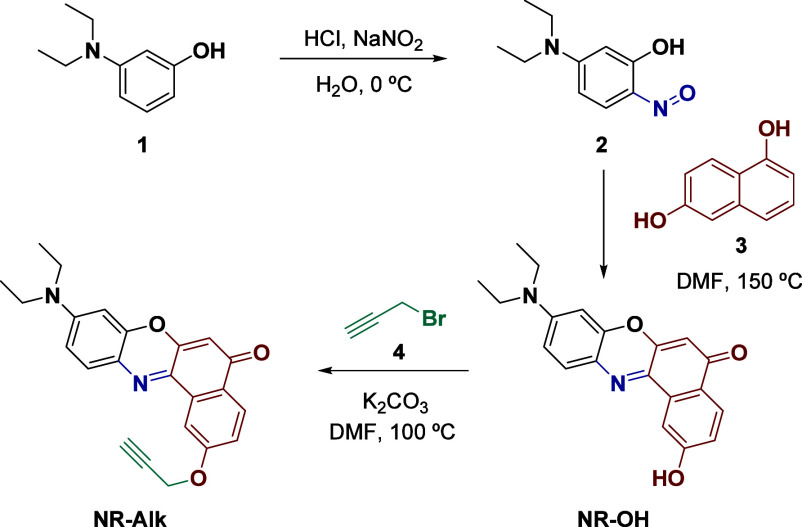
Synthesis of NR-Alk

### 3.2 Synthesis and Characterization of **Azide_0.17_-COF** and **NR_0.17_-COF**

The synthesis of the covalent organic framework containing azides as pendant groups (**Azide**_**0.17**_ **-COF**) was carried out by solvothermal Schiff’s base reaction of 1,3,5-tris­(4-aminophenyl) benzene (**TAPB**) with a mixture of 2,5-dimethoxyterephtalaldehyde (**DMTA**) and 2,5-bis­(2-azidoethoxy) terephthalaldehyde (**BAETA**) in a 1/6 ratio (x = 0.17) (Scheme 3, see the SI for the synthesis of these linkers). The successful polymerization of **Azide**_**0.17**_ **-COF** was followed by ^13^C-Cross-Polarization Magic Angle Spinning Nuclear Magnetic Resonance (^13^C–CP-MAS NMR) and Fourier transformed infrared (FTIR) spectroscopy (Figures S1 and S2). On the one hand, the ^13^C–CP-MAS NMR spectrum revealed imine linkages (ca. 160 ppm) along with the disappearance of aldehyde signals, while retaining the remaining anisochronous carbons. On the other hand, FTIR spectroscopy showed the disappearance of the aromatic amine and aldehyde functionalities (ca. 3400 cm^–1^ and 1667 cm^–1^ respectively), with the emergence of an imine band at 1622 cm^–1^. Moreover, the azide stretching band of the **BAETA** linker remains intact centered at 2100 cm^–1^, corroborating the retainment of the N_3_ groups after the solvothermal polymerization. In addition, powder X-ray diffraction (PXRD) was employed to analyze the crystalline nature of the obtained material, revealing the diffraction pattern of the expected honeycomb lattice with P6 symmetry and lattice parameters a = b=37.57 Å and c = 3.61 Å (Figure S3).^15a^ Thus, the diffraction maxima appearing at 2.9°, 4.92°, 5.7°, 7.51°, 9.8°, and 25.53° could be ascribed to the (100), (110), (200), (210) and (001) facets, respectively.

**Scheme 3:**
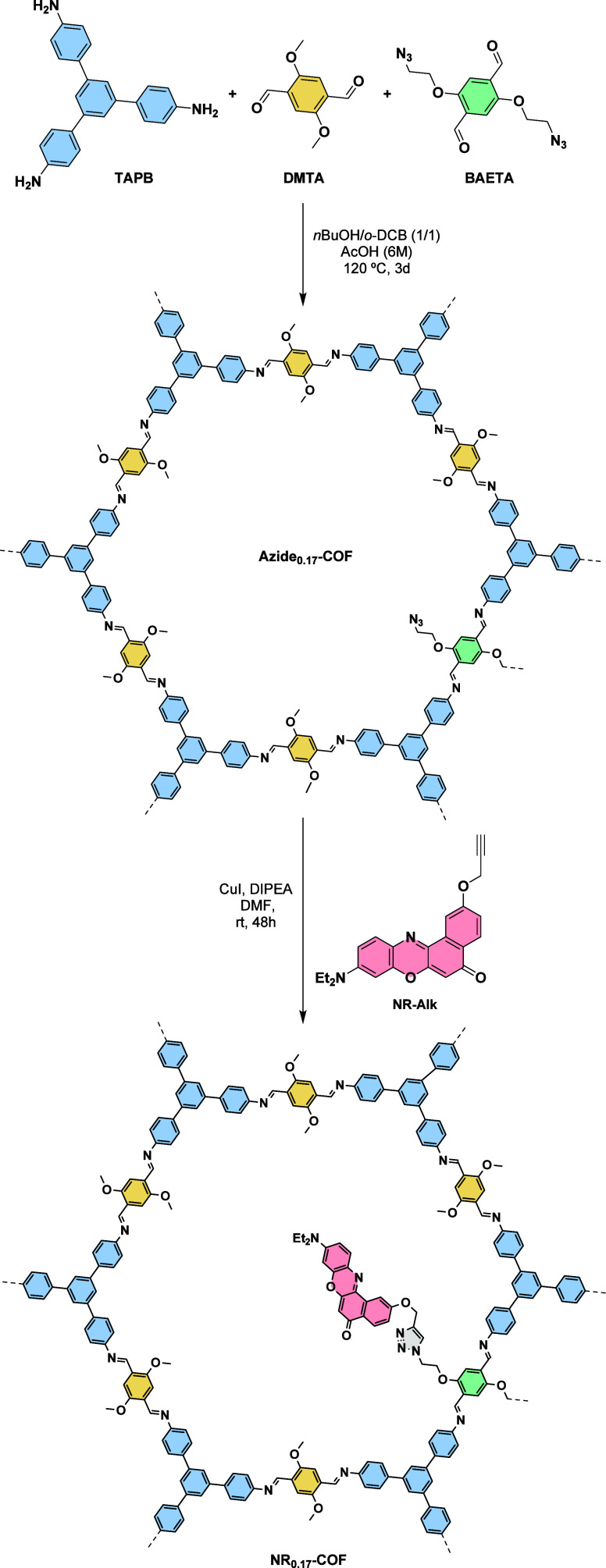
Synthesis of NR_0.17_-COF

Finally, the synthesis of **NR**_**0.17**_ **-COF** was carried out by postsynthetic reaction between **Azide**_**0.17**_ **-COF** and **NR-Alk** through a copper­(I) catalyzed azide–alkyne Hüisgen’s cycloaddition (Scheme 3, see the SI for more details). The progress of the transformation was followed by FTIR (Figure 1A and Figures S2 and S10) with the total disappearance of the azide (2100 cm^–1^) and the ≡C—H and C≡C stretching bands (ca. 3300 and 2125 cm^–1^) corresponding to **Azide**_**0.17**_ **-COF** and **NR-Alk**, respectively. Also, its ^13^C–CP-MAS NMR spectrum (Figure S9) shows the disappearance of the azide alpha carbons at 69 ppm and the absence of the alkyne signals (at 76 ppm) with the emergence of the carbonyl (182 ppm) and the aliphatic (62, 47, and 14 ppm) signals of the targeted material. These data confirm the quantitative click postsynthetic modification, which ensures a molar loading of 6 × 10^–4^ mmol of the NR unit/mg of host COF. Furthermore, regarding the crystallinity, the obtained **NR**_**0.17**_ **-COF** retains the diffraction pattern of **Azide**_**0.17**_ **-COF**, showing the diffraction maxima at 2.9°, 4.92°, 5.7°, 7.51°, 9.8°, and 25.53°, which correspond to the mentioned (100), (110), (200), (210), and (001) facets, respectively (Figure 1B and Figure S11). It should be noted that the relative intensity of these diffraction maxima of **NR**_**0.17**_ **-COF** decreased compared to those of **Azide**_**0.17**_ **-COF**, likely due to the random staking of the Nile Red moieties within the COF cavities. **NR**_**0.17**_ **-COF** exhibited P6 symmetry with lattice parameters of *a* = *b* = 37.21 Å and *c* = 3.70 Å, as determined by Rietveld refinement using the GSAS-II software (Figure S12). These results are consistent with the reported symmetry and lattice parameters of the **Azide**_**0.17**_ **-COF** intermediate and its postsynthetic modification,^15a,20^ confirming that the crystallinity of the framework remains unaltered after modification.

**Figure 1:**
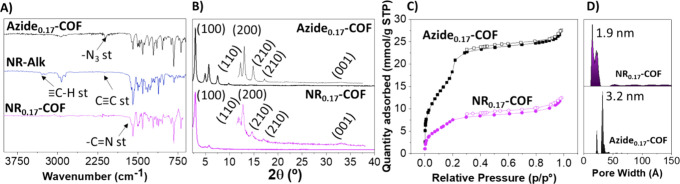
(A) FTIR spectra of **Azide**_**0.17**_ **-COF** (black), **NR-Alk** (blue), and **NR**_**0.17**_ **-COF** (magenta). (B) PXRD patterns of **Azide**_**0.17**_ **-COF** (black) and **NR**_**0.17**_ **-COF** (magenta). The inset shows a magnification in the 5.4–30° range. (C) N_2_ sorption isotherms of **Azide**_**0.17**_ **-COF** (black) and **NR**_**0.17**_ **-COF** (magenta). Empty symbols represent the desorption branches. (D) Pore size distribution of **Azide**_**0.17**_ **-COF** (black) and **NR**_**0.17**_ **-COF** (magenta).

In the next step, the porous features of the materials were investigated by measuring their nitrogen adsorption isotherms at 77K, which reveals a type IV isotherm (Figure 1C and Figures S4 and S13) for both COFs, as expected for mesoporous materials. However, it is important to remark that after postsynthetic modification the pore volume was reduced from 0.921 cm^3^/g to 0.385 cm^3^/g at 0.95 p/p^0^ and the Brunauer-Emmet-Teller (BET) surface area decreased from 1496 m^2^/g to 707 m^2^/g (Figures S5 and S14). Similarly, the pore size was calculated using nonlocal density functional theory (NLDFT), observing a width reduction from 3.2 to 1.9 nm (Figure 1D and Figures S6 and S15). This effect is attributed to the presence of pendant Nile Red moieties in the COF pores and correlates well with the observations found in PXRD. These data are in line with other reports in the literature of COFs with pendant groups anchored to their pore walls.^14,21^

The nanosized morphology of both products was also investigated by Scanning Electron Microscopy (SEM). SEM micrographs (Figure 2A and Figures S7 and S16) evidenced polygranular agglomerates of rod-like particles for both materials, demonstrating that the *click* reaction does not alter the morphology of the COF. Additionally, the products were suspended in a THF/H_2_O (7/3) mixture and subjected to an ultrasonic bath (3 min, 35 kHz, 80W) to produce colloids of the COFs and study their optical properties. Transmission electron microscopy (TEM) micrographs (Figure 2B and Figures S8 and S17) showed that the products retained the polygranular agglomerate morphology, with sizes around 100 nm for both materials. To conclude the characterization, the UV–vis spectra of the materials were recorded, confirming the incorporation of the Nile Red chromophore into the framework and showing no significant differences between the spectrum of **NR**_**0.17**_ **-COF** and that of a mixture consisting of **Azide**_**0.17**_ **-COF** and **NR-Alk** in the same proportion (Figure 2C). Interestingly, the absorption band at ca. 545 nm of **NR-Alk** and **NR**_**0.17**_ **-COF** aligns with that of Eosin Y, one of the most commonly used photocatalysts in organic reactions, which prompted us to select Eosin Y as the model homogeneous photocatalyst for studying the photocatalytic activity of the synthesized Nile Red-based COF.^22^

**Figure 2:**
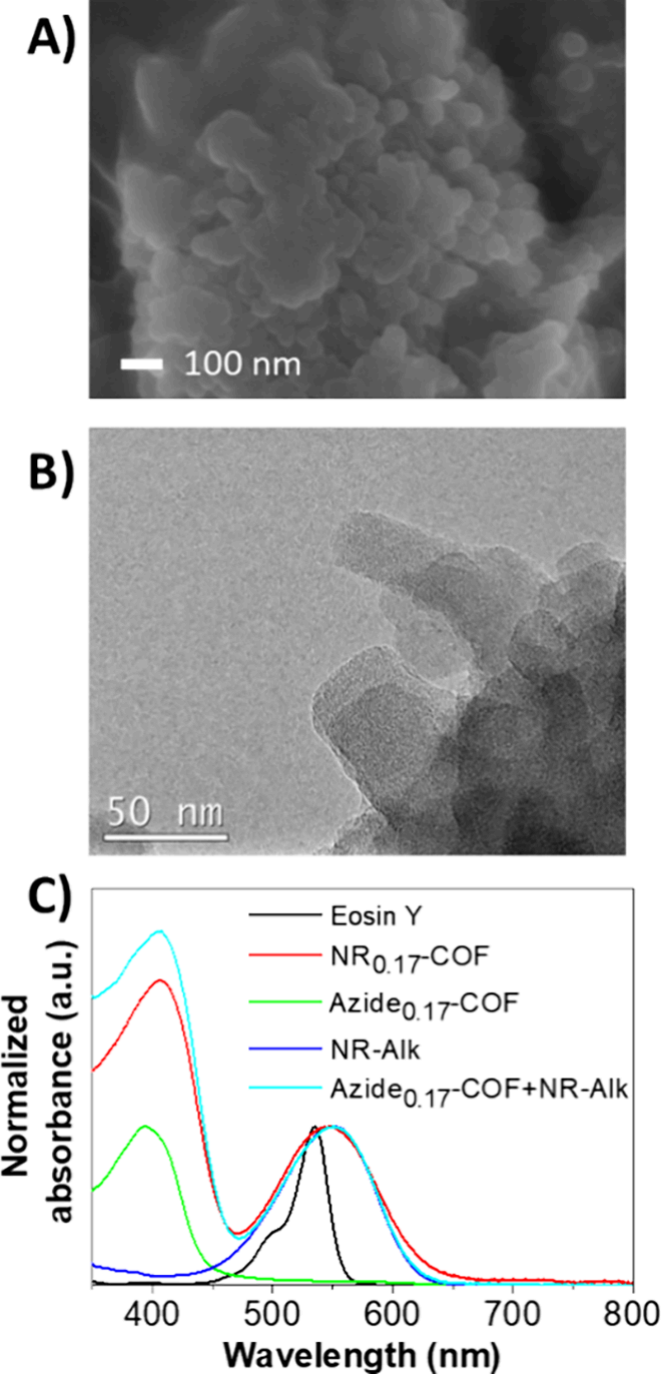
(A) SEM micrograph of **NR**_**0.17**_ **-COF**. (B) TEM micrograph of **NR**_**0.17**_ **-COF**. (C) UV–vis spectra of the compounds under study in a THF/H_2_O (7/3) mixture.

### 3.3 Photocatalytic Activity of **NR_0.17_-COF**

To evaluate the catalytic performance of **NR**_**0.17**_ **-COF** we selected light-mediated C–H functionalization reactions. These transformations have garnered significant interest over the past decades due to their ability to directly modify natural products, pharmaceuticals, petroleum feedstocks and polymers under mild conditions.^23^ In particular, the photocatalytic activity of our material was initially tested in the metal-free arylation of heteroarenes via aryldiazoniums, which was reported by König and co-workers using Eosin Y (Table 1).^24^ To our delight, **NR**_**0.17**_ **-COF** effectively catalyzed this reaction, achieving the arylated product **7a** with 71% yield under green light irradiation (Entry 1). This result closely resembles the yield obtained when we reproduced the experimental conditions from the original work on homogeneous catalysis but using our homemade photoreactor (72%, Entry 2). As control experiments, we carried out the transformation in the dark and without any catalyst (Entries 3 and 4), observing a significant decrease in conversion (20 and 23% respectively), which confirms that both light and the photocatalyst are essential for substantial product formation. In addition, the performance of the **Azide**_**0.17**_ **-COF** was evaluated (Entry 5), yielding the product **7a** at only 25%, indicating that the framework is not photoactive and highlighting the necessity of incorporating the Nile Red fragment into the structure.

**Table 1:**
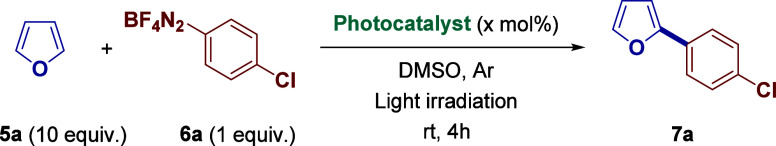
Initial Experiments for the Light-Mediated Arylation of Heteroarenes with Aryldiazonium Salts

**entry**	**PC(*x*** mol %)	**light**	**yield (%)** ^a^
1	**NR_0.17_-COF** (1 mol % NR)	green	71
2	Eosin Y (1 mol %)	green	72
3	**NR_0.17_-COF** (1 mol % NR)	dark	20
4	-	green	23
5	**Azide_0.17_-COF** (1 mol % N_3_)	green	25
6	NR-Alk (1 mol %)	green	25
7	NR-Alk (7 mol %)	green	62
8	**Azide_0.17_-COF** (1 mol % N_3_) + NR-Alk (1 mol %)	green	26
9	**NR_0.17_-COF** (1 mol % NR)	yellow	69
10	**NR_0.17_-COF** (1 mol % NR)	red	48

a Yields determined by ^1^H NMR spectroscopy. PC= photocatalyst. Reaction conditions: **5a** (2.3 mmol), **6a** (0.23 mmol), PC (1–7 mol %), and DMSO (1 mL).

To assess the advantage of **NR**_**0.17**_ **-COF** over the comparable homogeneous catalyst, the Nile Red molecule, we conducted the reaction employing our precursor **NR-Alk** (Entry 6). In this way, we observed that the same amount of photocatalyst (1 mol %) did not drive the process effectively, requiring 7 times more catalyst (7 mol %) to achieve the same result as in heterogeneous conditions (Entry 7), thus demonstrating the superior efficiency of the material. Due to this, a final control experiment using a mixture of **Azide**_**0.17**_ **-COF** and **NR-Alk** (1 mol %) was carried out, leading to the formation of **7a** in 26% yield, which confirms that the photoactive unit must be covalently attached to the backbone of the COF (Entry 8). Furthermore, given that the UV–vis absorption band of **NR**_**0.17**_ **-COF** is significantly broader than that of Eosin Y, we considered testing the methodology also with low-energy light. Fortunately, the reaction performed just as well under yellow light, yielding the arylated product **7a** with a 69% yield (Entry 9)^25^ and could even be carried out under red light, albeit with a reduced yield of 48% (Entry 10).

The applicability of our heterogeneous photocatalyst was also examined by reacting various aryl diazonium salts with different heteroarene groups (Scheme 4). Pleasingly, **NR**_**0.17**_ **-COF** allows the utilization of a range of substituted aromatic scaffolds containing either electron-donating or electron-withdrawing groups such as Cl, Br, NO_2_, CN or OMe, among others. Moreover, not only furan but also thiophene and pyrrole can be tolerated in the studied protocol. Thus, nine arylated products were isolated from moderate to very good yields (33–77%) applying our new catalytic system, which shows its excellent functional group tolerance, with no homocoupling products observed in any case.

**Scheme 4:**
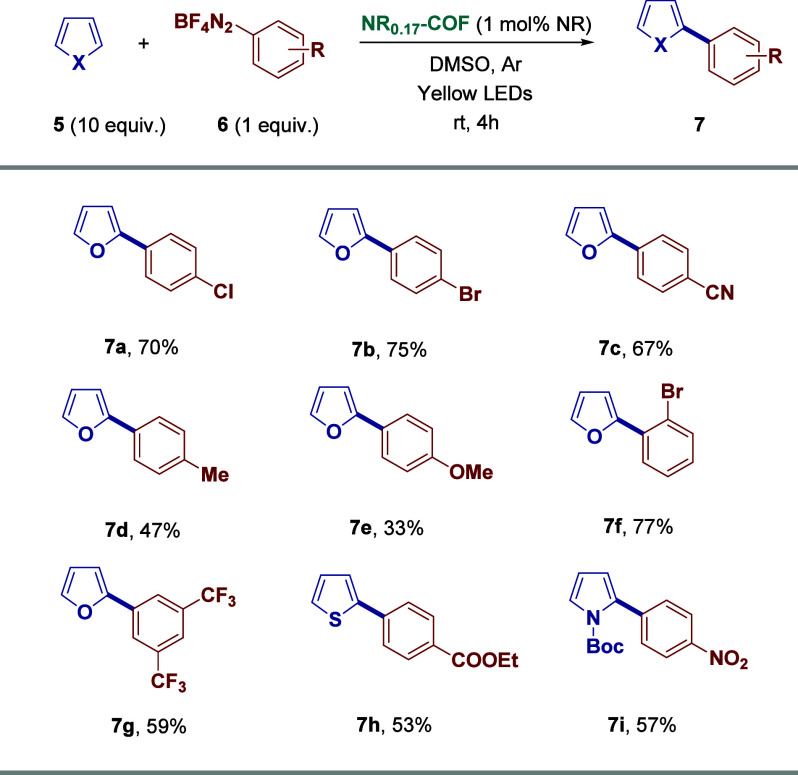
Substrate Scope of the Light-Mediated Arylation of Heteroarenes with Aryldiazonium Salts

Next, in order to explore the potential of **NR**_**0.17**_ **-COF** in light-mediated organic transformations, we aimed to investigate its performance in more complex C–H-functionalization reactions. It was observed that aromatic radicals, generated from diazonium salts, could be successfully employed in a cascade reaction involving alkyne insertion, followed by cyclization (Scheme 5a). In this manner, the material was able to replicate the results achieved by Eosin Y in homogeneous catalysis, yielding compound **9**, although with slightly lower yield (41%).^26^ Encouraged by this result, we advanced to test the activity of the heterogeneous photocatalyst in reactions involving radicals of different natures. Favorably, both sulfur-centered radicals, specifically thiocyanate radicals, and nitrogen-centered cationic radicals, were efficiently generated and selectively incorporated at the C3 position of the indole rings (Scheme 5b,c). In both cases, **NR**_**0.17**_ **-COF** successfully replaced now the homogeneous photocatalyst Rose Bengal, yielding excellent results not only in the thiocyanation reaction (**12**, 71%),^27^ but also in the preparation of formylated heteroarenes (**15**, 63%).^28^ These experiments demonstrate the remarkable versatility of the synthesized material, which exhibits good functionality in a wide range of solvents with diverse properties, such as DMSO, THF, MeOH, MeCN, and even water. Additionally, it can tolerate various reaction conditions, including elevated temperatures (60 °C) and noninert atmospheres, such as air or oxygen. Lastly, taking advantage of the broader UV–vis absorption spectrum of **NR**_**0.17**_ **-COF** compared to Eosin Y or Rose Bengal we performed the three reactions under yellow LED irradiation, successfully obtaining the expected final products in all cases, although with slightly lower yields.

**Scheme 5:**
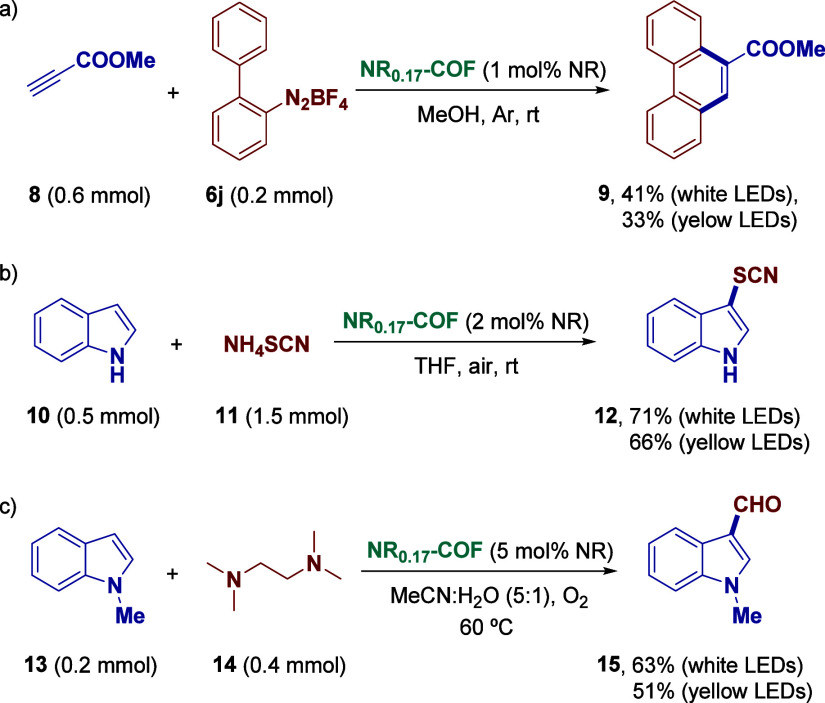
(a–c) **NR_0.17_-COF** as a Photocatalyst in Other Light-Mediated C–H Functionalization Reactions

### 3.4 Scale-Up and Recyclability Experiments

Considering the main drawbacks of homogeneous catalysis, we decided to test both the scalability and the recyclability of **NR**_**0.17**_ **-COF**. First, we evaluated the scalability of the system by conducting the model reaction with 2 mmol of starting material **6a**, which represents a 10-fold increase compared to the previous scale, while maintaining the catalyst loading at 1 mol % of NR. To our satisfaction, the final product **7a** was obtained after 4 h of irradiation with a yield of 68%, demonstrating consistent performance under these scaled-up conditions (Figure 3A). On the other hand, to probe its recyclability, the material was recovered by centrifugation at the end of the standard reaction protocol, washed three times with THF/Hexane (5:1) and reused for a new catalytic cycle. The procedure was repeated 6 times, showing minimal loss of activity and yielding 0.92 mmol (165 mg) of 2-(4-chlorophenyl)­furan (**7a**) using just 3.8 mg of heterogeneous photocatalyst (1 mol % of NR) (Figure 3B). Hence, in terms of turnover number (TON), **NR**_**0.17**_ **-COF** achieved 400 TON (with respect to NR) after six runs, confirming its robustness and recyclability. Moreover, the preservation of both its chemical identity and crystallinity was verified by comparing the FTIR spectra and PXRD patterns of the material before and after catalysis (Figure 3C and D).

**Figure 3:**
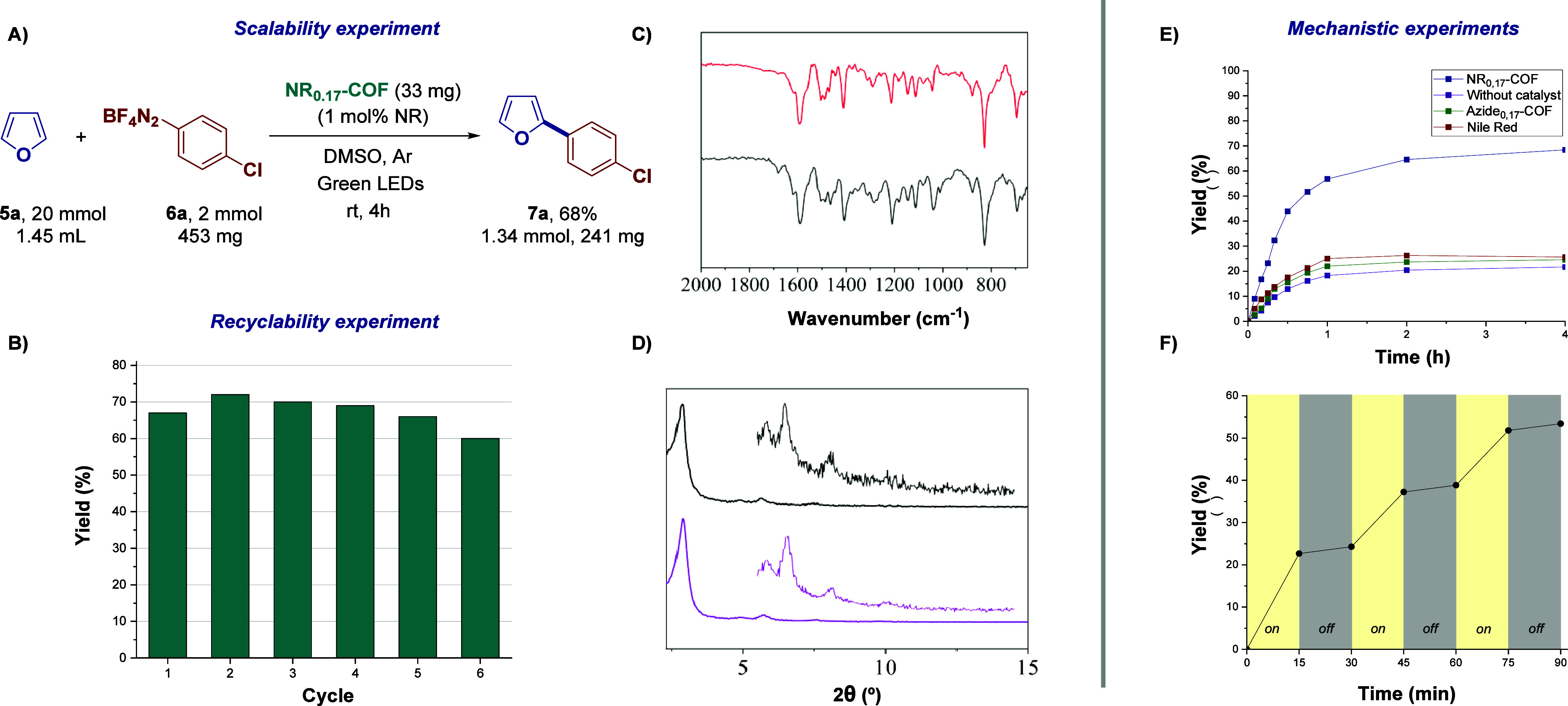
(A) Scaled-up standard reaction using **NR**_**0.17**_ **-COF**. (B) Recyclability of **NR**_**0.17**_ **-COF** in the standard reaction. (C) Comparison of FTIR spectra of **NR**_**0.17**_ **-COF** before (red) and after (black) catalysis. (D) Comparison of PXRD patterns of **NR**_**0.17**_ **-COF** before (black) and after (magenta) catalysis. (E) Kinetic profiles for the standard reaction with different catalysts. (F) “Light/dark” experiments for the standard reaction using **NR**_**0.17**_ **-COF**.

### 3.5 Mechanistic Experiments

To gain further insight into the mode of action of our material, a series of experiments were conducted using the protocol for the arylation of heteroarenes. Thus, the transformation of the model substrate was monitored by ^1^H NMR employing 1 mol % NR in both heterogeneous and homogeneous conditions. It was observed that **NR**_**0.17**_ **-COF** behaved as a straightforward catalyst, reducing the starting material **6a** in a smooth reaction without any induction period, reaching saturation within just 2 h (Figure 3E). In contrast, the homogeneous complex **NR-Alk**, exhibited the same profile as the framework without the photoactive fragment, **COF-N**_**3**_, and the blank reaction, which confirms again that NR is inactive at this concentration. In all three cases, the maximum conversion reached was between 20–25%, corresponding to the background level of the reaction, in agreement with the results observed in Table 1 (Figure 3E).

On the other hand, in order to confirm that the arylation protocol using our material proceeds via a radical mechanism, as in the homogeneous approach, we carried out a radical capture experiment, adding TEMPO to the mixture of aryl diazonium salt **6a**, furan, and **NR**_**0.17**_ **-COF**. In this case, the formation of the final compound was partially suppressed, yielding **7a** in only 19% yield, while a TEMPO-trapped intermediate was isolated as the mayor product (38%), suggesting that the reaction follows a radical pathway (for more details, see the SI). Likewise, a “light/dark” experiment was performed to investigate the effect of irradiation on the heterogeneous photocatalytic process. For this purpose, after 15 min of illumination, the light was switched off, and the reaction system was kept in the dark for an equivalent period, repeating the operation three times. As shown in Figure 3F, the conversion was temporarily suppressed during the dark phases (with only a slight increase, likely due to the aforementioned background reaction), which does not completely rule out the involvement of a radical chain mechanism.^29^

Finally, the electrochemical properties of **NR**_**0.17**_ **-COF** were studied in comparison with those of the **NR-Alk** molecule and the material without the photoactive fragment, **Azide**_**0.17**_ **-COF** (Figure 4). Cyclic voltammograms recorded at glassy carbon (GC) electrodes modified with **NR**_**0.17**_ **-COF,** in 0.1 M TBAP (tetrabutylammonium perchlorate)/ acetonitrile solution previously deoxygenated using argon, showed an onset oxidation potential of +0.32 V and an onset reduction potential of −0.56 V. From these values, the valence band (4.72 eV) and conduction band (3.84 eV) of the material can be estimated, resulting in a band gap of 0.88 eV (for more details, see the SI).

**Figure 4:**
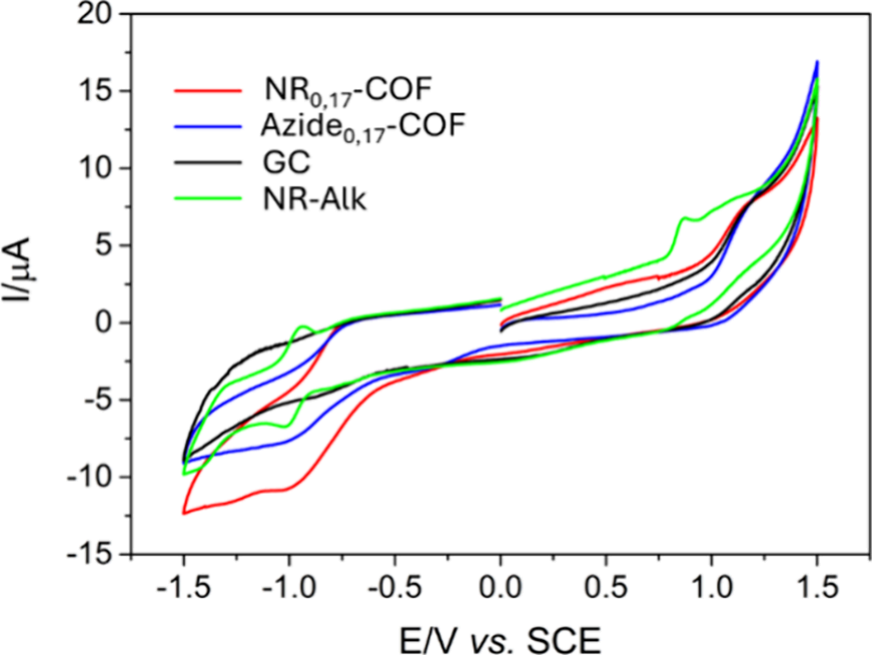
Cyclic voltammograms recorded at modified glassy carbon with carbon black (black) and with **NR**_**0.17**_ **-COF/**carbon black (red) and **Azide**_**0.17**_ **-COF/**carbon black (blue) in a 0.1 M TBAP/acetonitrile solution in the absence of O_2_. Cyclic voltammograms were recorded at modified glassy carbon with carbon black in the presence of 1 mg/mL **NR-Alk** (green) in 0.1 M TBAP/acetonitrile solution in the absence of O_2_.

Based on the above information, a plausible mechanism for the standard transformation is outlined in Scheme 6.^30^ First, irradiation of **NR**_**0.17**_ **-COF** with visible light generates the excited state of the photoredox catalyst, which induces single-electron transfer (SET) to the aryl diazonium salt (*E*_red_ from −0.3 to 0.2 V),^31^ forming aryl radical **16**. Next this aryl radical captures the heteroarene, yielding intermediate **17**, which is subsequently oxidized by the radical cation of **NR**_**0.17**_ **-COF**, leading to carbocation **18**. Lastly, deprotonation of **18** restores aromaticity, affording the obtained product **7**. Additionally, the possibility that oxidation of intermediate **17** may also occur via the aryl diazonium salt **6** through a radical chain transfer mechanism cannot be ruled out.^32^

**Scheme 6:**
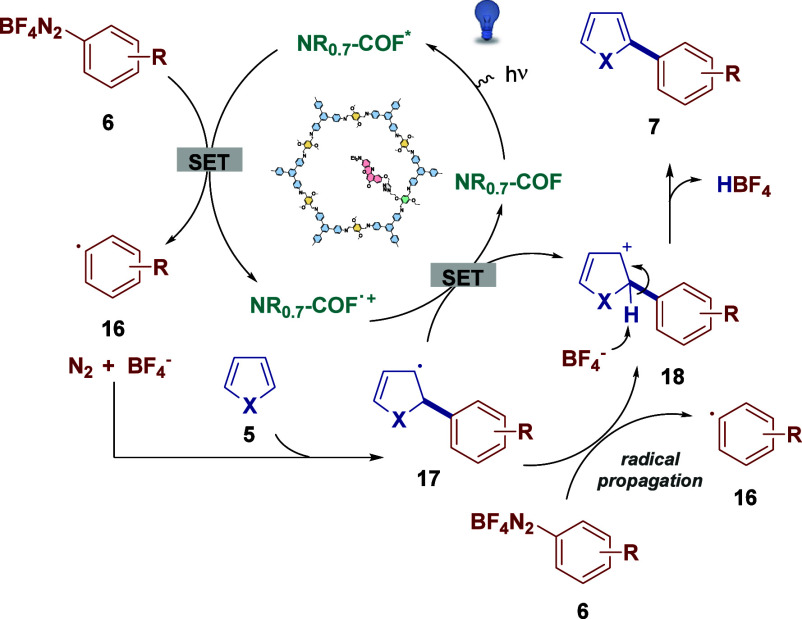
Plausible Mechanism for Light-Mediated Arylation of Heteroarenes with **NR_0.17_-COF** as a Catalyst

## 4 Conclusions

In summary, we have performed the predesign, synthesis, and characterization of a new Nile Red-based COF, which demonstrates high catalytic activity toward various photomediated C–H functionalization reactions. This **NR**_**0.17**_ **-COF** exhibits notable versatility and excellent functional group tolerance, capable of utilizing different precursors to form aryl, sulfur, and nitrogen radicals, while operating under distinct reaction conditions. Moreover, the properties of the synthesized material confer the advantage of enabling reactions with low-energy light by using a minimal amount of the photoactive fragment. Finally, compared to homogeneous photocatalysis, our heterogeneous system shows clear superiority by addressing key challenges such as scalability and recyclability. Thus, the reaction was easily scaled up by approximately 10-fold, and the catalyst could be recovered and recycled up to six times, yielding a TON of 400 for Nile Red.

## Supplementary Material



## Notes

Experimental procedures, characterization of compounds, and NMR spectra of products (PDF)
