# Supplementary material for: Nile Red-Based Covalent Organic Framework as a Photocatalyst for C–H Bond Functionalization
Source: ACS Catal. 2025 Jun 6;15(12):10736–45. doi: 10.1021/acscatal.5c02173 (PMC12186703; doi:10.1021/acscatal.5c02173)

Supporting Information

Nile Red-based Covalent Organic Framework as a Photocatalyst for C−H bond Functionalization

Marta Gordo-Lozano,[a#] Diego G. Matesanz,[b#] Marcos Martínez-Fernández,[a#] Pedro Almendros,[c] Emiliano Martínez-Periñán,[d] José L. Segura,[a*] and Sara Cembellín[b*]

[a] Macromolecular and Heterocyclic Organic Materials Group, Organic Department, Facultad de Ciencias Químicas, Universidad Complutense de Madrid, 28040 Madrid, Spain

[b] Green Catalysis Laboratory, Organic Department, Facultad de Ciencias Químicas, Universidad Complutense de Madrid, 28040 Madrid, Spain; and Center for Innovation in Advanced Chemistry (ORFEO−CINQA).

[c] Instituto de Química Orgánica General, IQOG-CSIC, Consejo Superior de Investigaciones Científicas, 28006 Madrid, Spain.

[d] Sensors and Biosensors group. Departamento de Química Analítica y Análisis Instrumental, Facultad de Ciencias, and Institute for Advanced Research in Chemical Sciences (IAdChem), Universidad Autónoma de Madrid, Campus de Cantoblanco, Madrid 28049, Spain.

segura@ucm.es
scembellin@ucm.es

Supporting Information

Table of Contents

1. General Information........ S3

2. Procedure and Analytical Data of COF precursors S4

3. Procedure and Analytical Data of COFs........ S9

4. Synthesis of Substrates for Catalytic Reactions S17

5. Initial Experiments for the Arylation of Heteroarenes........ S17

6. Procedure and Analytical Data of Arylated Products **7** S19

7. Procedure and Analytical Data of Compounds **9**, **12** and **15**........ S22

8. Scale-up Experiment........ S24

9. Recyclability Experiment S25

10. Kinetic Experiments........ S26

11. Radical Capturing Experiments S28

12 “Light/dark” Experiment S29

13. Electrochemical Measurements........ S29

14. Comparison of **$NR_{0.17}$-COF** with Other Photocatalysts........ S31

15. References........ S31

16. NMR Spectra of Products........ S33

1. General Information

All reactions were conducted in oven-dried glassware (120 °C). Solvents and commercially available chemicals were obtained from Merck, VWR, Fisher Scientific, Scharlab, Alfa Aesar and Fluorochem, and used without further purification.

Reaction temperatures are reported as the temperature of the heat transfer medium surrounding the vessel unless otherwise stated.

Analytical thin layer chromatography (TLC) was performed on silica gel 60 F254 aluminum plates (Merck) and they were visualized by exposure to short wave ultraviolet light (254 nm, 366 nm) and/or by staining. For staining the TLC plates were dipped into a solution of $KMnO_4$ (1 g $KMnO_4$, 6 g K_2CO_3 and 0.1 g KOH in 100 mL H_2O) or solution of vanillin (2 g vanillin in 150 mL ethanol and 5 mL H_2SO_4) and developed with a heat gun if necessary. Flash chromatography was performed on Scharlab silica gel (40-63 mesh) by standard techniques using appropriate mixtures of hexane and ethyl acetate with compressed air.

^{1}H- and ^{13}C-NMR spectra were recorded at room temperature on a Bruker AVIII HD 300MHz BACS-60, Bruker Neo 300MHz, Bruker AVIII HD-WB 400MHz and Bruker AVIII 700MHz in deuterated solvents as indicated or in solid state. Chemical shifts (δ) for ^{1}H- and ^{13}C-NMR spectra are given in ppm relative to tetramethylsilane (TMS) using the residual solvent signals as references for ^{1}H and ^{13}C NMR spectra ($CDCl_3$: δ_H = 7.26 ppm, δ_C = 77.06 ppm; DMSO-d_6: δ_H = 2.50 ppm, δ_C = 39.53 ppm;). ^{13}C-NMR spectra were acquired on a broadband decoupled mode. Chemical shifts are generally reported with two (^{1}H) or one (^{13}C) digits after the decimal point. NMR-data are reported as follows: chemical shift (multiplicity [s = singlet, d = doublet, dd = double doublet, dt = double triplet, t = triplet, q = quartet, m = multiplet, br = broad], coupling constants (*J*, Hz) and integration). All spectra were processed using the MestreNova program.

Fourier transformed infrared (FT-IR) spectroscopy was performed on a PerkinElmer 100 spectrophotometer equipped with a PIKE Technologies MIRacle Single Reflection Horizontal ATR Accessory and on a Bruker TENSOR 27 on a diamond plate.

Mass spectroscopy (MS) was performed on a Bruker Model HCT Ultra Ion Trap Mass Spectrometer (Mass rage:50-6000 amu) coupled to HPLC with ESI, APCI and NS interfaces.

Powder X-ray diffraction (PXRD) measurements were carried out with X'PERT MPD with conventional Bragg-Brentano geometry using monochromatic Cu Kα1 radiation (λ = 1.5406 Å) in the 2θ = 1.8°- 40° range.

For N_2 sorption isotherms, N_2 (77 K) adsorption-desorption measurements were carried out on a Micromeritics Tristar 3000 and samples were previously activated for 4 h under high vacuum high vacuum (<10^{-7} bar) at 120 °C.

UV-vis absorption spectra were recorded on a Varian Cary 50 Scan UV-VIS Spectrophotometer and emission spectra on a Jasco FP-6300 Spectrofluorometer.

Scanning Electron Microscopy (SEM) images were acquired on a JEOL JSM7600F microscope.

Transmission Electron Microscopy (TEM) images were recorded on a JEOL JEM 2100 microscope.

Photocatalytic reactions were performed in a homemade photoreactor with LEDs of different wavelengths and 18 W of intensity, as the set-up showed below:

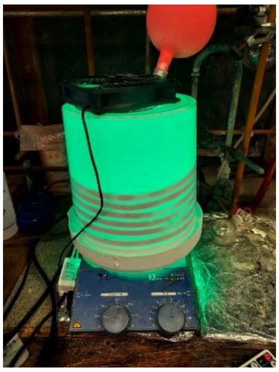

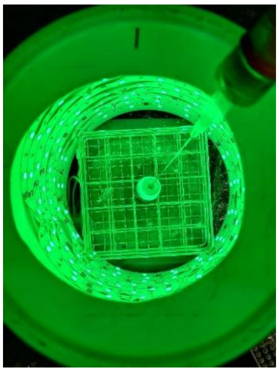

Measured temperature (with fan on) = 24 ºC

2. Procedure and Analytical Data of COF precursors

*Synthesis of 5-diethylamino-2-nitrosophenol (**2**):*

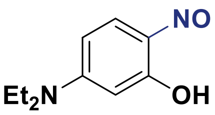

In a 100 mL round bottom flask 3-diethylaminophenol (**1,** 36 mmol, 6 g) were dissolved in water (8 mL) and concentrated HCl (14 mL). After cooling the mixture to 0 ºC, a solution of $NaNO_2$ (36 mmol,

2.49 g) in water (20 mL) was added dropwise to the solution and the mixture was stirred at 0 ºC for 4 hours. Afterwards, the crude was filtered, and the solid was washed with a solution of HCl 4M, and finally dried. The obtained solid **2** was used without further purification in the next step. ^{1}H-NMR spectrum is in accordance with the literature.[1]

*Synthesis of 9-diethylamino-2-hydroxy-5*H*-benzo[*a*]phenoxazin-5-one (**NR-OH**)*

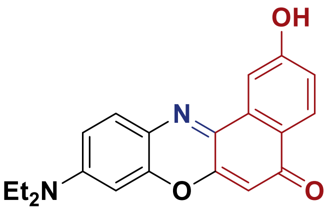

In a 250 mL round bottom flask 5-diethylamino-2-nitrosophenol (**2**, 5 mmol, 1 g) and 1,6-dihydroxynaphtol (**3**, 4.6 mmol, 0.73 g) were added and dissolved in DMF (90 mL). The solution was heated at reflux overnight. Then, the solvent was removed via vacuum evaporation and the resulting solid was purified by flash chromatography (silica gel; gradient from hexane/EtOAc 9:1 to hexane/EtOAc 1:1). The compound **NR-OH** was obtained (300 mg, 20%) as a purple solid. **^{1}H-NMR (300 MHz, DMSO-d$_6$)** δ 10.45 (s, 1H), 7.97 (d, J= 8.6 Hz, 1H), 7.88 (d, *J* = 2.4 Hz, 1H), 7.57 (d, *J* = 9.1 Hz, 1H), 7.10 (dd, J= 8.6 Hz, 2.5 Hz, 1H), 6.80 (dd, *J* = 9.2, 2.7 Hz, 1H), 6.63 (d, *J* = 2.6 Hz, 1H), 6.15 (s, 1H), 3.54 – 3.47 (m, 4H), 1.15 (d, *J* = 6.0 Hz, 6H). **^{13}C-NMR (75 MHz, DMSO-d$_6$)** δ 182.5, 161.5, 152.5, 151.6, 147.3, 139.6, 134.6, 131.7, 128.3, 124.8, 119.3, 111.0, 109.0, 105.1, 98.0, 45.3, 13.3.

*Synthesis of 9-(diethylamino)-2-(prop-2-yn-1-yloxy)-5*H*-benzo[*a*]phenoxazin-5-one (**NR-Alk**):*

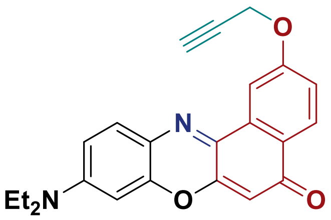

NR-Alk was synthesized according to a reported procedure with slight modifications.[2] In a 25 mL double necked round bottom flask with a reflux system, 9-diethylamino-2-hidroxy-5*H*-benzo[*a*]phenoxazin-5-one (**NR-OH**, 0.45 mmol, 150 mg) and K_2CO_3 (1.73 mmol, 239 mg) were added, and the system was filled with argon. After the solids were solved in DMF (3.4 mL), propargyl bromide (**4**, 1.8 mmol, 0.2 mL) are added dropwise, and then, the mixture was heated at 100 °C overnight. Afterwards, solvent was eliminated under reduced pressure and the residue was purified by flash chromatography (silica gel; hexane/EtOAc 2:1). The compound **NR-Alk** was obtained (80.9 mg, 48%) as a purple solid. **^{1}H-NMR (300 MHz, CDCl$_3$)** δ

8.25 (d, *J* = 8.7 Hz, 1H), 8.15 (d, *J* = 2.6 Hz, 1H), 7.62 (d, *J* = 9.1 Hz, 1H), 7.24 (dd, *J* = 8.7, 2.7 Hz, 1H), 6.66 (dd, *J* = 2.7, 9.1 Hz, 1H), 6.47 (d, *J* = 2.7 Hz, 1H), 6.32 (s, 1H), 4.9 (d, *J* = 2.4 Hz, 2H), 3.48 (q, *J* = 7.1 Hz, 4H), 2.58 (t, *J* = 2.4 Hz, 1H), 1.25 (t, *J* = 2.4 Hz, 6H). **^{13}C-NMR (75 MHz, $CDCl_3$)** δ 183.2, 179.9, 162.5, 160.1, 150.2, 139.9, 131.2, 127.9, 118.4, 109.5, 105.3, 96.4, 76.0, 56.1, 45.1, 36.3, 29.7, 12.6. **FTIR (ATR) (cm^{-1})** 3290, 3221, 2969, 2922, 2853, 2116, 1624, 1594, 1500, 1470, 1405, 1346, 1316, 1268, 1251, 1221, 1183, 1114, 1084, 1015, 819.

*Synthesis of 1,3,5-Tris-(4-nitrophenyl)benzene (**TNPB**):*

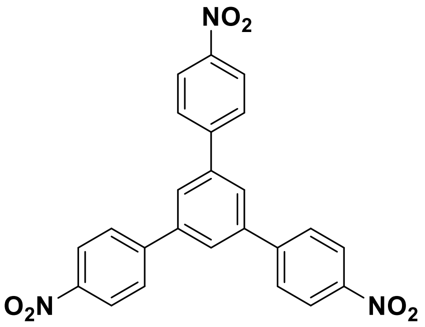

In a 100 mL double necked round bottom flask connected to a Dean-Stark, 4-nitroacetophenone (60.55 mmol, 10 g) was introduced and dissolved in toluene (50 mL). Trifluoro- methanesulfonic acid (0.25 mL) was added and the reaction was heated to reflux for 72 hours. The resulting black solution was cooled to 0 ºC and the black precipitate was isolated via filtration. The obtained solid was washed in a Soxhlet extractor with DMF and methanol until the solid turns pale grey. Once dry, **TNPB** product (5.6944 g, 64%) was obtained as an insoluble solid in al common deuterated solvents. **FTIR (ATR) (cm^{-1})** 1596, 1508, 1344, 1249, 1105, 863, 840, 814, 749, 690.

*Synthesis of 1,3,5-Tris-(4-aminophenil)benzene (**TAPB**):*

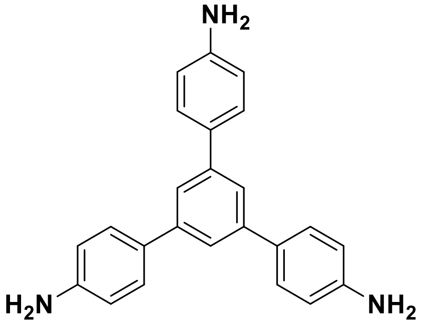

Under argon atmosphere, in a 250 mL round bottom flask **TNPB** (4.53 mmol, 2.0 g) and of carbon palladium catalyst (10 mol%, 0.2 g) were suspended in ethanol (80 mL) and the mixture was heated to reflux. Once heated, hydrated hydrazine (285.4 mmol, 20.5 mL) was added dropwise, and the reaction was heated for 10 hours. Then, the crude was filtered over celite and recrystallized from ethanol yielding **TAPB** (984.6 mg, 62%) as yellow crystals. **^{1}H-NMR**

(300 MHz, DMSO-d$_6$) δ 7.53 – 7.43 (m, 9H), 6.66 (d, J = 8.5 Hz, 6H), 5.21 (s, 6H). **^{13}C-NMR (75 MHz, DMSO-d$_6$)** δ 145.9, 142.0, 131.9, 128.2, 122.9, 115.4. **FTIR (ATR) (cm^{-1})** 3434, 3344, 3222, 3029, 1613, 1511, 1451, 1404, 1280, 1178, 826, 560. **MS m/z (ESI)**: calc. for $C_{24}H_{21}N_3$ (M+H$^+$) 352.18, found 352.1.

*Synthesis of dimethoxyterephtaldehyde (**DMTA**):*

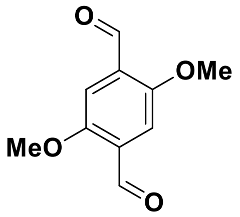

In a 100 mL Schlenk tube 1,4-dibromo- 2,5-dimethoxybenzene (6.77 mmol, 2.0 g) were dissolved in anhydrous deoxygenated THF (34 mL) at -78ºC under Argon. Then, BuLi (25 mmol, 10 mL) were added dropwise, and the reaction was stirred for 3 hours. Afterwards, anhydrous DMF (2.0 mL) were added, stirring for another hour. The crude was warmed up to room temperature and was poured over 150 mL of a saturated NH_4Cl solution. The solvent was removed under reduced pressure and the aqueous phase was extracted with DCM. Then, the organic phase was washed with water, dried with anhydrous $MgSO_4$, filtered and concentrated under reduced pressure. After purification with flash chromatography (silica gel; hexane/EtOAc 9:1, increasing gradually the polarity) the product **DMTA** (370.8 mg, 28%) was obtained as a yellow solid. **^{1}H-NMR (300 MHz, CDCl$_3$)** δ 10.49 (s, 2H), 7.45 (s, 2H), 3.94 (s, 6H). **^{13}C-NMR (75 MHz, CDCl$_3$)** δ 189.3, 155.7, 129.1, 110.9, 56.2. **FTIR (ATR) (cm^{-1})** 2872, 1680, 1478, 1401, 1301, 1214, 1130, 1028, 880, 659. **MS m/z (MALDI-TOF):** calc. for $C_8H_6O_4$ (M+H$^+$) 194.1, found 194.1.

*Synthesis of dihydroxyterephtaldehyde (**DHTA**):*

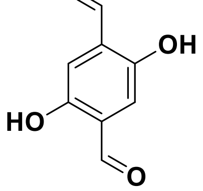

In a 25 mL double necked round bottom flask **DMTA** (1.75 mmol, 340 mg) were dissolved in DCM (6.8 mL) at 0ºC under Argon atmosphere. Afterwards, of BBr_3 (70.6 mmol, 6.8 mL) was added and the reaction was stirred at room temperature for 8 hours. The crude was extracted with DCM and the organic phase was washed with water, dried with anhydrous $MgSO_4$, filtered and concentrated under reduced pressure. Then, a recrystallisation in EtOAc afforded the product **DHTA** (147.1 mg, 51%) as a yellow

solid. **^{1}H-NMR (300 MHz, CDCl$_3$)** δ 10.23 (s, 2H), 9.96 (s, 2H), 7.24 (s, 2H). **^{13}C-NMR (75 MHz, CDCl$_3$)** δ 190.2, 152.8, 127.7, 115,2. **FTIR (ATR) (cm^{-1})** 3280, 2888, 1668, 1477, 1281, 1127, 888, 832, 796, 679. **MS m/z (MALDI-TOF):** calc. for $C_8H_6O_4$ (M-H$^+$) 166.03, found 165.0.

*Synthesis of 2,5-bis(2-bromoethoxy)terephtaldehyde (**BBETA**)*

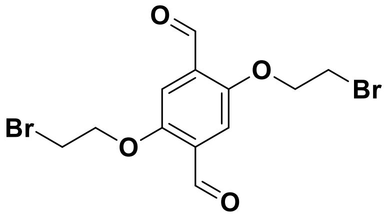

In a 25 mL round bottom flask **DHTA** (0.21 mmol, 35 mg) and K_2CO_3 (1.27 mmol, 174.8 mg) were solved in of anhydrous DMF (2.5 mL). The mixture was heated to 65 ºC and dibromoethane (6.33 mmol, 0.55 mL) were added and then, the reaction was stirred for 8 hours. The crude was extracted with DCM and the organic phase was washed with water, dried with anhydrous $MgSO_4$, filtered and concentrated under reduced pressure. After a purification via flash chromatography (silica gel; DCM) the product **BBETA** was obtained analytically pure (25.4 mg, 32%) as a yellow solid. **^{1}H-NMR (300 MHz, CDCl$_3$)** δ 10.56 (s, 2H), 7.45 (s, 2H), 4.46 (t, J = 5.6 Hz, 4H), 3.71 (t, J = 5.6 Hz, 4H). **^{13}C-NMR (75 MHz, CDCl$_3$)** δ 188.8, 154.7, 129.6, 112.2, 68.9, 28.8.

*Synthesis of 2,5-bis(2-azidoethoxy)terephtaldehyde (**BAETA**)*

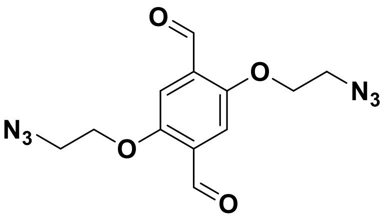

In a 25 mL round bottom flask **BBETA** (0.52 mmol, 198.3 mg) and sodium azide (1.88 mmol, 122.12 mg) were added and dissolved in anhydrous DMF (10.2 mL). The reaction was stirred for 8 hours at 85 ºC. After that time, the crude was extracted with DCM and the organic phase was washed with water, dried with anhydrous $MgSO_4$, filtered and concentrated under reduced pressure. The product **BAETA** (143.1 mg, 90%) was obtained as a yellow solid without further purification. **^{1}H-NMR (300 MHz, CDCl$_3$)** δ 10.53 (s, 2H), 7.48 (s, 2H), 4.31 (t, J = 4.7 Hz, 4H), 3.69 (t, J = 4.9 Hz, 4H). **^{13}C-NMR (75 MHz, CDCl$_3$)** δ 188.6, 154.7, 129.3, 111.9, 68.2, 50.2. **FTIR (ATR) (cm^{-1})** 2947, 2922, 2884, 2150, 2108, 2087, 2066, 1667, 1480, 1462, 1426, 1405, 1387, 1281, 1247, 1208, 1129, 1056, 999, 950,

875, 838, 692, 638. **MS m/z (ESI)**: calc. for $C_{12}H_{12}N_6O_4$ (M^+) 304.27, found 304.516.

3. Procedure and Analytical Data of COFs

*Synthesis of **Azide$_{0.17}$-COF**:*

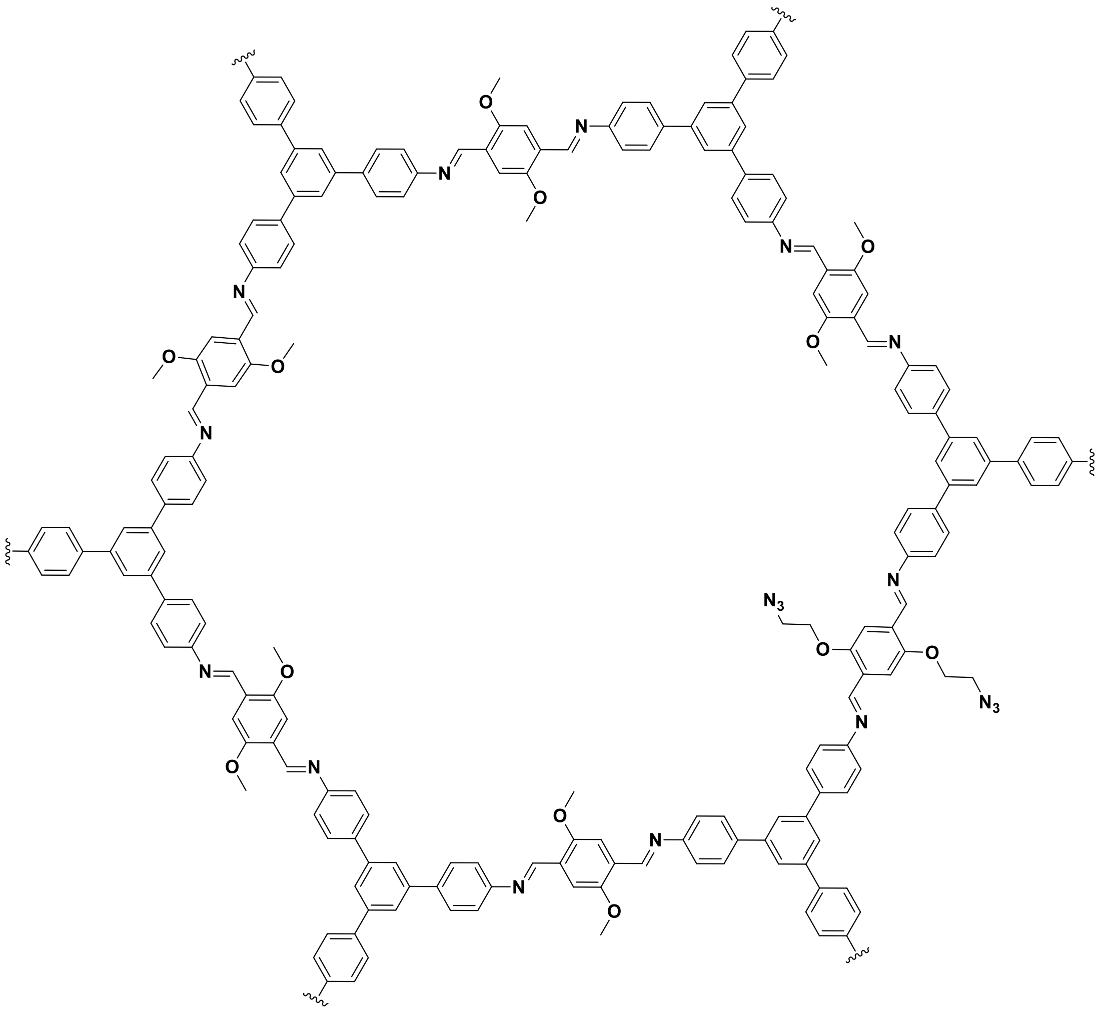

In a Pyrex tube **DMTA** (0.87 mmol, 169.25 mg), **BAETA** (0.18 mmol, 54.75 mg) and **TAPB** (0.7 mmol, 246.25 mg) were introduced. Then, a mixture of *ortho*-dichlorobenzene (4.75 mL), butanol (4.75 mL) and a solution of acetic acid (0.93 mL, 6M) were added. The mixture was sonicated and put through 3 freeze-pump-thaw cycles. The tube was flame-sealed, and the reaction was heated at 120 ºC for 3 days. The resulting solid was filtered and washed with THF, ethanol and again THF. After drying the solid, the polymer **Azide$_{0.17}$-COF** (423 mg, 98%) was obtained as a yellow solid. **^{13}C-NMR-CP-MAS (75 MHz)** δ 154.48, 140.40, 128.47, 122.77, 109.87, 68.82,

54.25. **FTIR (ATR) (cm^{-1})** 2106, 2095, 1615, 1591, 1505, 1489, 1464, 1443, 1409, 1374, 1289, 1210, 1181, 1144, 1040, 1013, 971, 879, 828, 732, 693. **PXRD (2Θ) (º)** 2.873, 4.909, 5.687, 7.509, 9.813. **Formula:** $C_{79}H_{61}N_9O_6$. **Porosity:** 1496 m^{2}g^{-1} (BET surface area); 0.921 cm^{3}g^{-1} (pore volume at 0.95 p/pº); 3.2 nm (pore size).

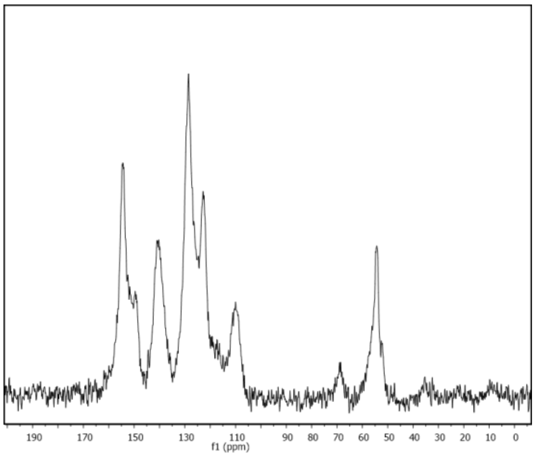

Figure S1. ^{13}C-NMR-CP-MAS spectrum of **Azide$_{0.17}$-COF**.

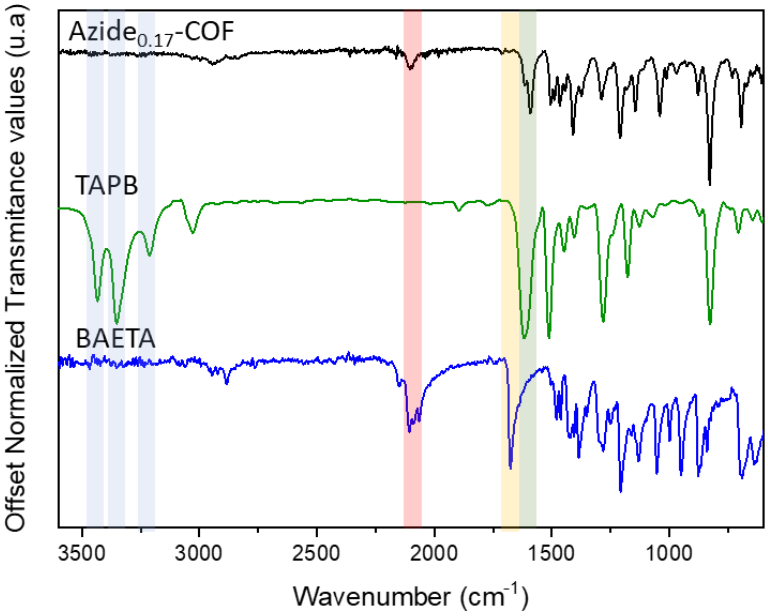

Figure S2. FTIR spectra analysis for the **Azide$_{0.17}$-COF** (black) formation. **TAPB** (green) and **BAETA** (blue). The disappearance of amine and aldehyde functionalities are highlighted in blue and yellow, respectively. The retainment of azide moieties is highlighted in red. Appearance of imine band is highlighted in green.

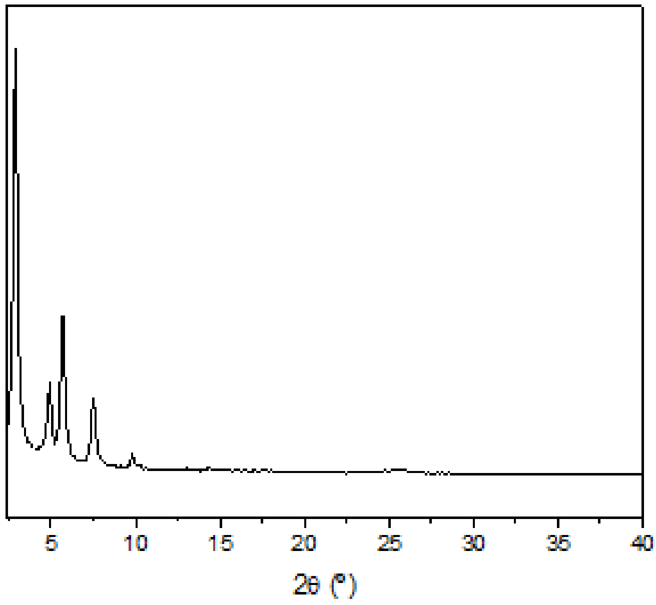

Figure S3. PXRD of **Azide$_{0.17}$-COF**.

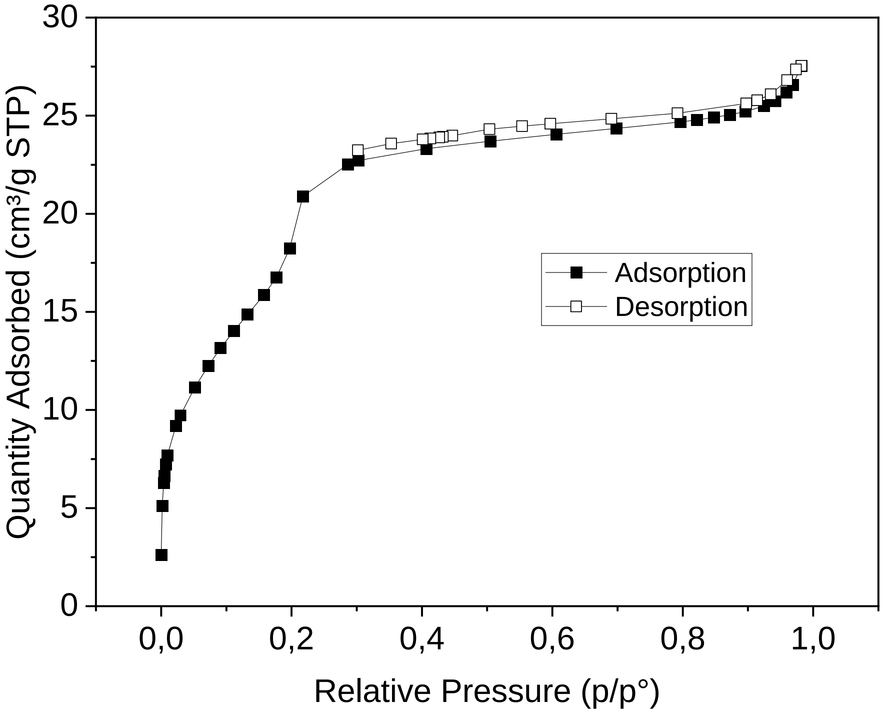

Figure S4. N_2 sorption isotherm for **Azide$_{0.17}$-COF**.

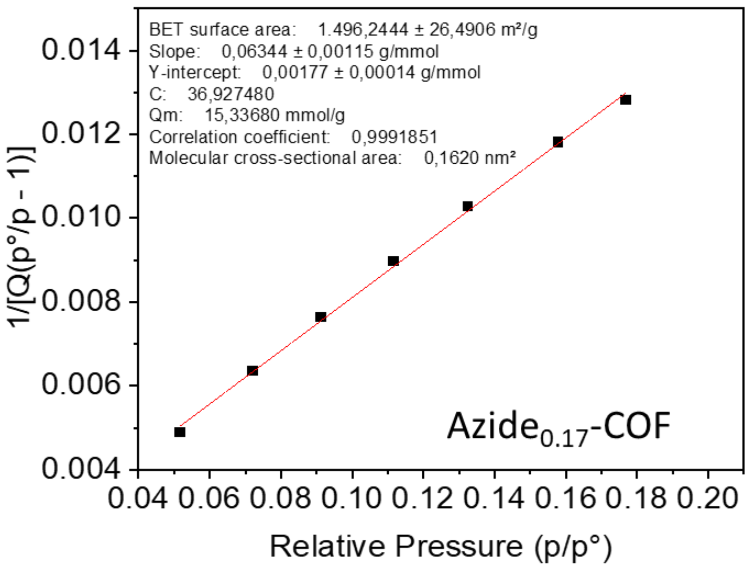

Figure S5. BET plot for N_2 sorption for **Azide$_{0.17}$-COF**.

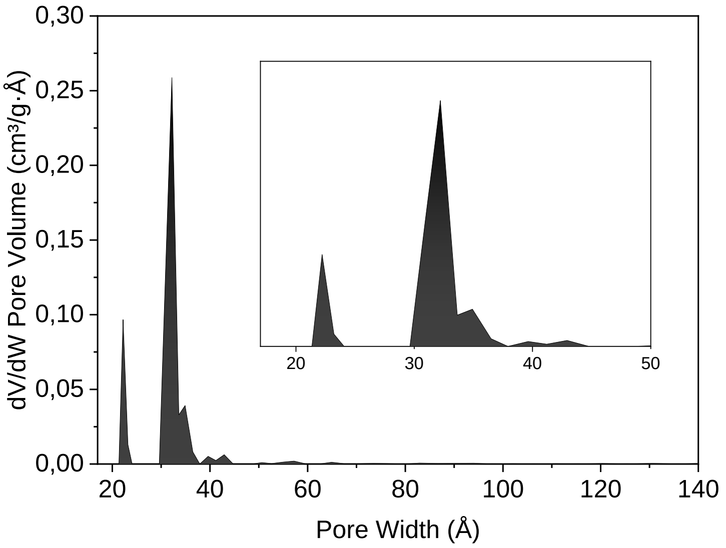

Figure S6. Full pore size distribution for **Azide$_{0.17}$-COF**, and magnification between 10 and 50 Amstrong.

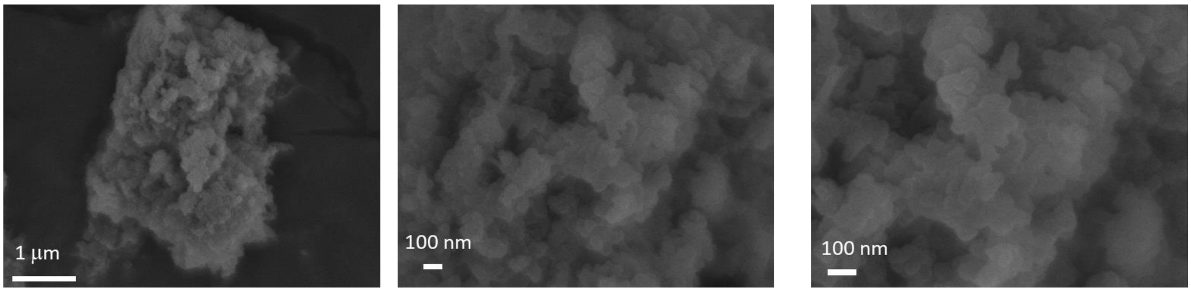

Figure S7. SEM micrographs of **Azide$_{0.17}$-COF**. Left: scale bar 1 µm (20.000x). Center: scale bar 100 nm (60.000x). Right: scale bar 100 nm (90.000x).

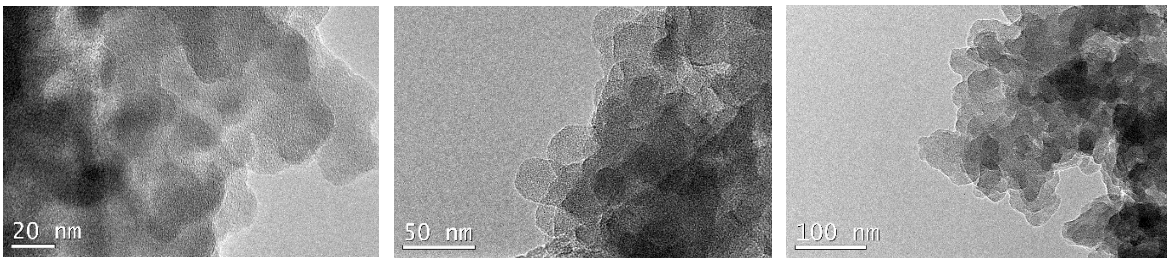

Figure S8. TEM micrographs of **Azide$_{0.17}$-COF**. Left: scale bar 20nm. Center: scale bar 50 nm. Right: scale bar 100 nm.

*Synthesis of **NR$_{0.17}$-COF**:*

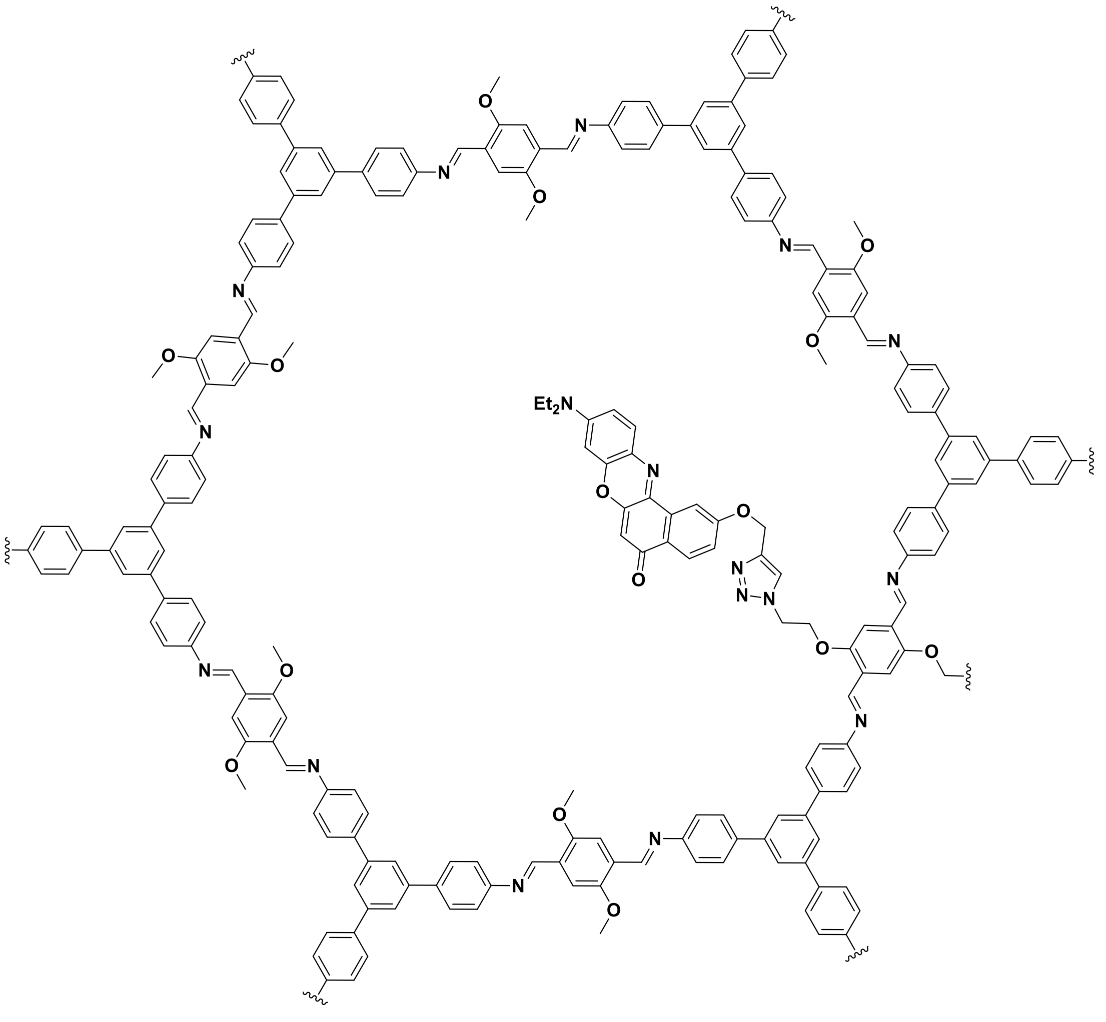

In a 100 mL round bottom flask **Azide$_{0.17}$-COF** (7.65×10^{-4} mmol N_3/mg COF), 110 mg) and CuI (0.046 mmol, 8.8 mg) were added, and the system was left under vacuum. Afterwards, it was filled with argon and a solution of **NR-Alk** (0.093 mmol, 34.7 mg) in deoxygenated DMF (19.14 mL) was added. Sequentially, DIPEA (0.252 mmol, 43.9

μL) was added and the flask was stirred at room temperature for 48 hours. The resulting solid was collected by filtration and washed with DMF, acetonitrile, THF and hexane. Finally, the polymer **$NR_{0.17}$-COF** (106.1 mg, quantitative yield) was obtained as a purple solid. **^{13}C-NMR-CP-MAS (75 MHz)** δ 182.8, 153.2, 139.2, 127.2, 121.2, 108.7, 96.4, 62.9, 54.8, 45.2, 12.6. **FTIR (ATR) (cm^{-1})** 2997, 2929, 1593, 1502, 1490, 1469, 1439, 1414, 1317, 1288, 1208, 1182, 1144, 1110, 1081, 1047, 878, 832, 692. **PXRD (2Θ) (º)** 2.902, 4.930, 5.746, 7.554, 9.803. **Formula:** $C_{101}H_{81}N_{11}O_9$. **Porosity:** 707 m^2g^{-1} (BET surface area); 0.385 cm^3g^{-1} (pore volume at 0.95 p/pº); 1.9 nm (pore size).

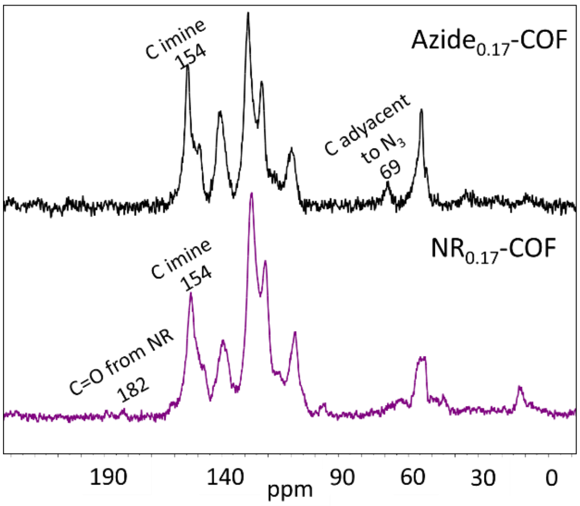

Figure S9. ^{13}C-NMR-CP-MAS spectra of **$Azide_{0.17}$-COF** and **$NR_{0.17}$-COF**.

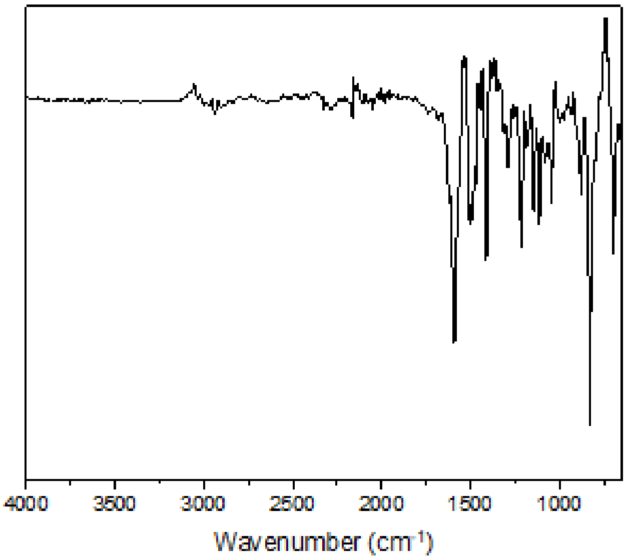

Figure S10. FTIR spectra of **$NR_{0.17}$-COF**.

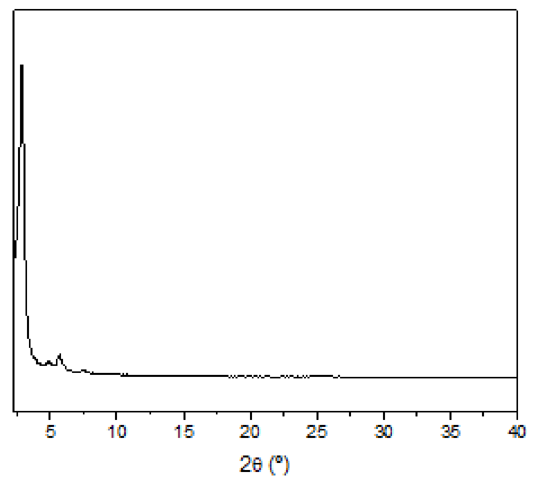

Figure S11. PXRD of **$NR_{0.17}$-COF**.

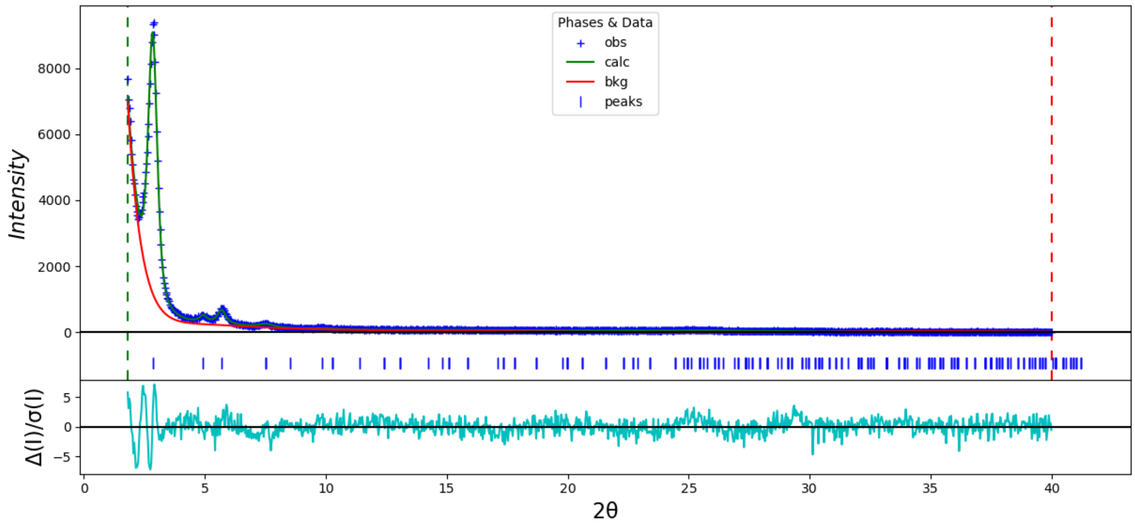

Figure S12. Results of Rietveld refinement for **$NR_{0.17}$-COF**. (R_w= 8.317%; χ^2=2.177).

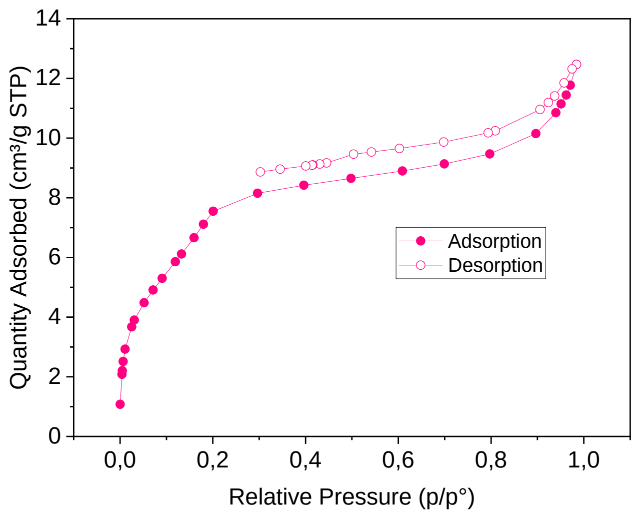

Figure S13. N_2 sorption isotherm for **$NR_{0.17}$-COF**.

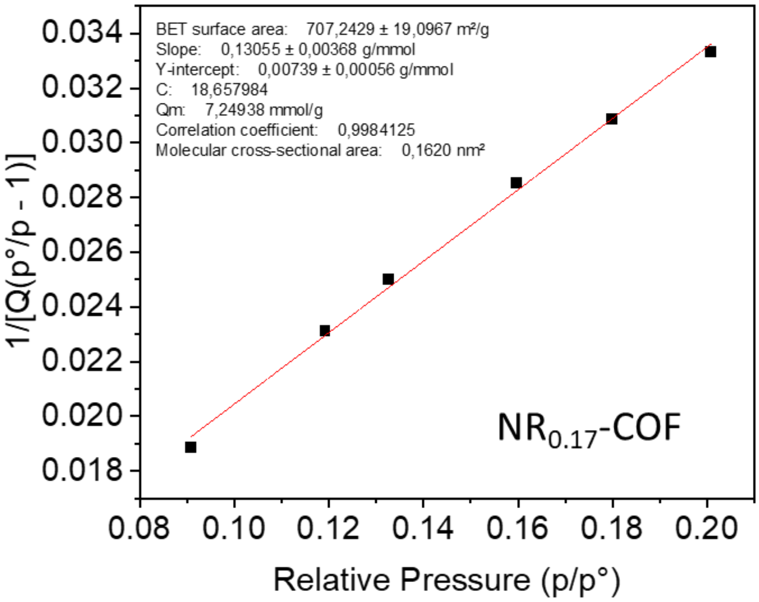

Figure 14. BET plot for N_2 sorption for **$NR_{0.17}$-COF**.

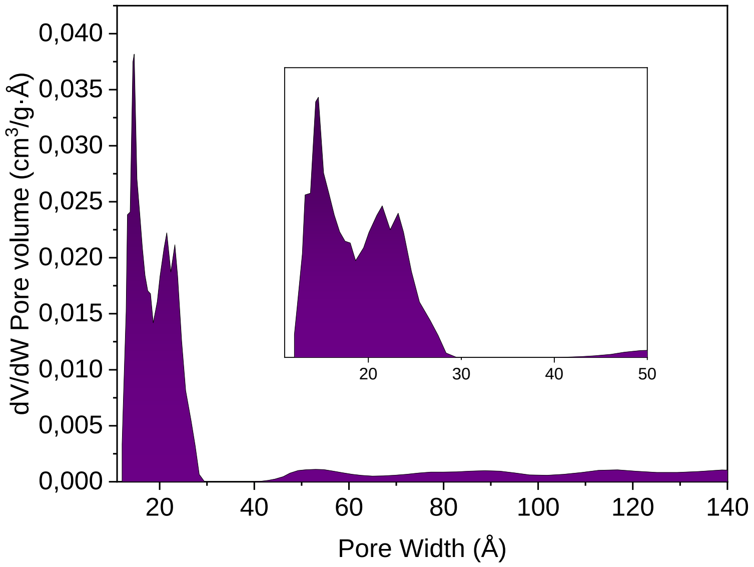

Figure S15. Full pore size distribution for **$NR_{0.17}$-COF**, and magnification between 10 and 50 Amstrong.

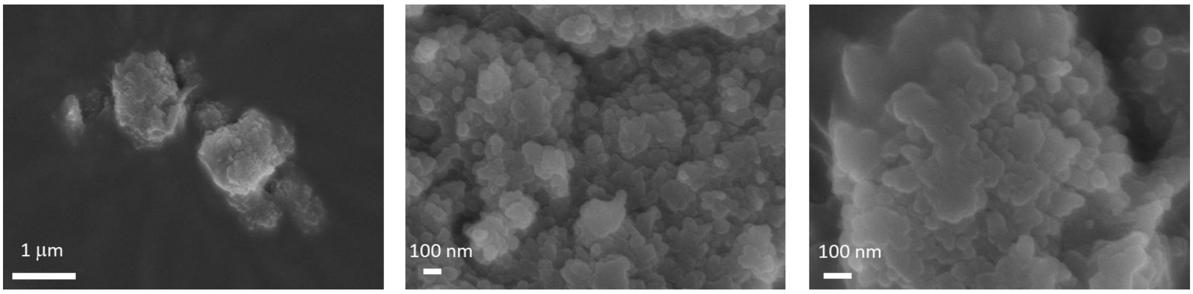

Figure S16. SEM micrographs of **$NR_{0.17}$-COF**. Left: scale bar 1 µm (20.000x). Center: scale bar 100 nm (60.000x). Right: scale bar 100 nm (90.000x).

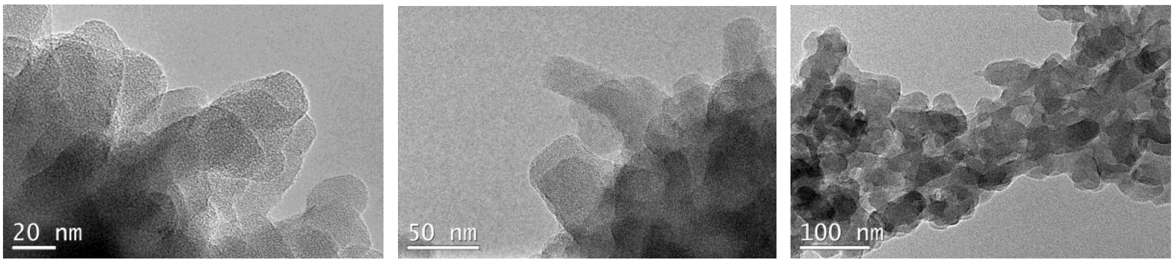

Figure S17. TEM micrographs of **NR$_{0.17}$-COF**. Left: scale bar 20 mm. Center: scale bar 50 nm. Right: scale bar 100 nm.

4. Synthesis of Substrates for Catalytic Reactions

Aryldiazonium salts **6a-j** were synthesized from the corresponding anilines according to the procedure described in the literature.[3] All the reactions were employed without any further optimization.

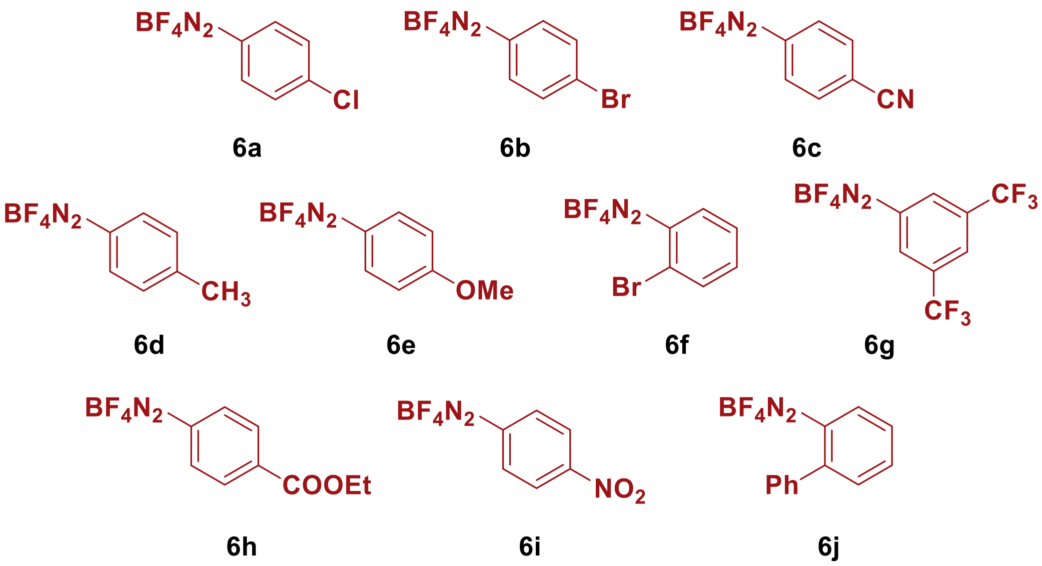

Figure S18. Synthesized aryldiazonium salts **6a-j**.

5. Initial Experiments for the Arylation of Heteroarenes

General procedure for the initial experiments:

In a 20 mL glass vial with a stirring bar, 4-chlorobenzenediazonium tetrafluoroborate **6a** (0.23 mmol, 1 equiv.) and **PC** (1-7 mol%) were added under air. Then, the reaction vessel was filled with argon. Afterwards, furane **5a** (2.3 mmol, 10 equiv.) and

sequentially deoxygenated anhydrous DMSO (1.0 mL) were added under an argon atmosphere, and the mixture was stirred at room temperature under green LEDs irradiation for 4 hours. After this time, the reaction mixture was transferred to a separating funnel, diluted with EtOAc and washed with water. The aqueous layer was washed three times with EtOAc. The organic layers were combined, dried with anhydrous $MgSO_4$, filtered and concentrated under reduced pressure. The yield was determined by ^{1}H-NMR analysis using nitromethane (0.1 mmol, 5.6 µL) as the internal standard.

Table S1. Initial experiments for arylation of heteroarenes.

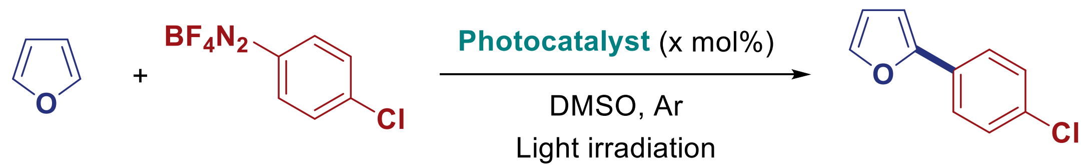

5a (10 equiv.) **6a** (1 equiv.) **7a**

Entry	Photocatalyst	Amount of PC	Light	Yield of 7a (%)
1	**NR$_{0.17}$-COF**	1 mol% NR	Green LEDs	71
2	Eosin Y	1 mol%	Green LEDs	72
3	**NR$_{0.17}$-COF**	1 mol% NR	Dark	20
4	-	-	Green LEDs	23
5	**Azide$_{0.17}$-COF**	1 mol% N_3	Green LEDs	25
6	**NR-Alk**	1 mol%	Green LEDs	25
7	**NR-Alk**	7 mol%	Green LEDs	62
8	**Azide$_{0.17}$-COF + NR-Alk**	1 mol% N_3 + 1 mol%	Green LEDs	26
9	**NR$_{0.17}$-COF**	1 mol% NR	Yellow LEDs	69
10	**NR$_{0.17}$-COF**	1 mol% NR	Red LEDs	48

DMSO = dimethylsulfoxide. NR = Nile Red. PC = photocatalyst.

6. Procedure and Analytical Data of Arylated Products 7

General procedure for the synthesis of arylated products ***7****:*

In a 20 mL glass vial with a stirring bar, diazonium salt **6** (0.23 mmol, 1 equiv.) and **$NR_{0.17}$-COF** (1 mol% NR, 3.8 mg) were added under air. Then, the reaction vessel was filled with argon. Afterwards, heteroaromatic substrate **5** (2.3 mmol, 10 equiv.) and sequentially deoxygenated anhydrous DMSO (1.0 mL) were added under an argon atmosphere, and the mixture was stirred at room temperature under yellow LEDs irradiation for 4 hours. After this time, the reaction mixture was transferred to a separating funnel, diluted with EtOAc and washed with water. The aqueous layer was washed three times with EtOAc. The organic layers were combined, dried with anhydrous $MgSO_4$, filtered and concentrated under reduced pressure. Finally, the product **7** was obtained by flash chromatography (silica gel; hexane/EtOAc).

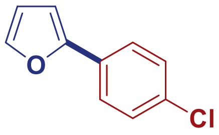

7a

2-(4-Chlorophenyl)furan (7a): Following the general procedure, the reaction of 4-chlorobenzenediazonium tetrafluoroborate (**6a**) (0.23 mmol, 52.1 mg), furane (**5a**) (2.3 mmol, 170 µL) and **$NR_{0.17}$-COF** (1 mol% NR, 3.8 mg) in DMSO (1 mL) at room temperature under yellow LEDs, afforded the product **7a** (70%, 28.7 mg) as a white solid. **^{1}H-NMR (300 MHz, $CDCl_3$)** δ 7.60 (dt, *J* = 8.7, 2.0 Hz, 2H), 7.48 (dd, *J* = 1.8, 0.8 Hz, 1H), 7.36 (dt, *J* = 8.7, 2.0 Hz, 2H), 6.64 (dd, *J* = 3.4, 0.8 Hz, 1H), 6.48 (dd, *J* = 3.4, 1.8 Hz, 1H). **^{13}C-NMR (75 MHz, $CDCl_3$)** δ 153.1, 142.5, 133.1, 129.5, 129.0, 125.1, 111.9, 105.6. NMR spectra are in accordance with the literature.[3]

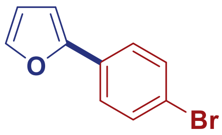

7b

2-(4-Bromophenyl)furan (7b): Following the general procedure, the reaction of 4-bromobenzenediazonium tetrafluoroborate (**6b**) (0.23 mmol, 62.3 mg), furane (**5a**) (2.3 mmol, 170 µL) and **$NR_{0.17}$-COF** (1 mol% NR, 3.8 mg) in DMSO (1 mL) at room temperature under yellow LEDs, afforded the product **7b** (75%, 38.3 mg) as a white solid. **^{1}H-NMR (300 MHz, $CDCl_3$)** δ 7.57 – 7.50 (m, 4H), 7.47 (dd, *J* = 1.8, 0.8 Hz, 1H), 6.65 (dd, *J* = 3.4, 0.8 Hz, 1H), 6.47 (dd, *J* = 3.4, 1.8 Hz, 1H). **^{13}C-NMR (75 MHz, $CDCl_3$)** δ 153.1,

142.5, 131.9, 129.9, 125.4, 121.2, 111.9, 105.7. NMR spectra are in accordance with the literature.[3]

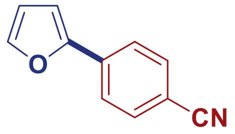

7c

4-(Furan-2-yl)benzonitrile (7c): Following the general procedure, the reaction of 4-cyanobenzenediazonium tetrafluoroborate (**6c**) (0.23 mmol, 49.9 mg), furane (**5a**) (2.3 mmol, 170 μL), and **$NR_{0.17}$-COF** (1 mol% NR, 3.8 mg) in DMSO (1 mL) at room temperature under yellow LEDs, afforded the product **7c** (67%, 25.9 mg) as a white solid. **^{1}H-NMR (300 MHz, $CDCl_3$)** δ 7.74 (d, J = 8.7 Hz, 2H), 7.65 (d, J = 8.7 Hz, 2H), 7.54 (dd, J = 1.8, 0.7 Hz, 1H), 6.81 (dd, J = 3.5, 0.8 Hz, 1H), 6.53 (dd, J = 3.4, 1.8 Hz, 1H). **^{13}C-NMR (75 MHz, $CDCl_3$)** δ 152.1, 143.8, 134.8, 132.7, 124.1, 119.1, 112.4, 110.4, 108.3. NMR spectra are in accordance with the literature.[3]

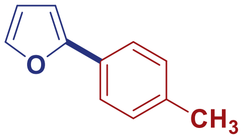

7d

2-(*P*-tolyl)furan (7d): Following the general procedure, the reaction of 4-methylbenzenediazonium tetrafluoroborate (**6d**) (0.23 mmol, 47.4 mg), furane (**5a**) (2.3 mmol, 170 μL), and **$NR_{0.17}$-COF** (1 mol% NR, 3.8 mg) in DMSO (1 mL) at room temperature under yellow LEDs, afforded the product **7d** (47%, 17.1 mg) as a white solid. **^{1}H-NMR (300 MHz, $CDCl_3$)** δ 7.58 (d, J = 8.2 Hz, 2H), 7.45 (dd, J = 1.8, 0.8 Hz, 1H), 7.20 (dt, J = 7.9, 0.7 Hz, 2H), 6.60 (dd, J = 3.4, 0.8 Hz, 1H), 6.46 (dd, J = 3.3, 1.8 Hz, 1H), 2.37 (s, 3H). **^{13}C-NMR (75 MHz, $CDCl_3$)** δ 154.4, 141.8, 137.3, 129.5, 128.4, 123.9, 111.7, 104.3, 21.4. NMR spectra are in accordance with the literature.[3]

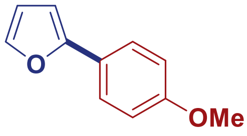

7e

2-(4-methoxyphenyl)furan (7e): Following the general procedure, the reaction of 4-methoxybenzenediazonium tetrafluoroborate (**6e**) (0.23 mmol, 51.0 mg), furane (**5a**) (2.3 mmol, 170 μL) and **$NR_{0.17}$-COF** (1 mol% NR, 3.8 mg) in DMSO (1 mL) at room temperature under yellow LEDs, afforded the product **7e** (33% 13.2 mg) as a white solid. **^{1}H-NMR (300 MHz, $CDCl_3$)** δ 7.60 (d, J = 8.8 Hz, 2H), 7.43 (dd, J = 1.8, 0.8 Hz, 1H), 6.92 (d, J = 8.8 Hz, 2H), 6.51 (dd, J = 3.3, 0.8 Hz, 1H), 6.44 (dd, J = 3.3, 1.8 Hz, 1H), 3.84 (s, 3H). **^{13}C-NMR (75 MHz, $CDCl_3$)** δ 159.2, 154.2, 141.5, 125.4, 124.2, 114.3, 111. 7, 103.5, 55.5. NMR spectra are in accordance with the literature.[3]

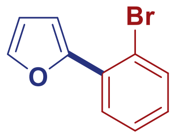

7f

2-(2-Bromophenyl)furan (7f): Following the general procedure, the reaction of 2-bromobenzenediazonium tetrafluoroborate (**6f**) (0.23 mmol, 62.3 mg), furane (**5a**) (2.3 mmol, 170 μL), and **$NR_{0.17}$-COF** (1 mol% NR, 3.8 mg) in DMSO (1 mL) at room temperature under yellow LEDs, afforded the product **7f** (77%, 39.7 mg) as a white solid. **^{1}H-NMR (300 MHz, $CDCl_3$)** δ 7.80 (dd, J = 7.9, 1.7 Hz, 1H), 7.66 (dd, J = 8.1, 1.3 Hz, 1H), 7.53 (dd, J = 1.8, 0.7 Hz, 1H), 7.36 (ddd, J = 7.9, 7.3, 1.3 Hz, 1H), 7.18 (dd, J = 3.4, 0.7 Hz, 1H), 7.13 (ddd, J = 8.0, 7.3, 1.7 Hz, 1H), 6.53 (dd, J = 3.4, 1.8 Hz, 1H). **^{13}C-NMR (75 MHz, $CDCl_3$)** δ 151. 5, 142.4, 134.2, 131.4, 128.9, 128. 5, 127.5, 119. 8, 111.5, 110.7. NMR spectra are in accordance with the literature.[4]

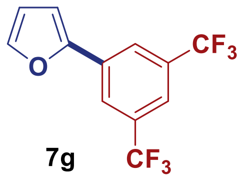

7g

2-(3,5-Bis(trifluoromethyl)phenyl)furan (7g): Following the general procedure, the reaction of 3,5-bis(trifluoromethyl) benzenediazonium tetrafluoroborate (**6g**) (0.23 mmol, 75.4 mg), furane (**5a**) (2.3 mmol, 170 μL) and **$NR_{0.17}$-COF** (1 mol% NR, 3.8 mg) in DMSO (1 mL) at room temperature under yellow LEDs, afforded the product **7g** (59%. 38.0 mg) as a white solid. **^{1}H-NMR (300 MHz, $CDCl_3$)** δ 8.07 (d, J = 1.7 Hz, 2H), 7.73 (t, J = 1.6 Hz, 1H), 7.55 (dd, J = 1.8, 0.7 Hz, 1H), 6.85 (dd, J = 3.5, 0.8 Hz, 1H), 6.55 (dd, J = 3.5, 1.8 Hz, 1H). **^{13}C-NMR (75 MHz, $CDCl_3$)** δ 151.1, 143.8, 132.8, 132.3 (q, J = 33.4 Hz), 123.6, 123.4 (q, J = 272.9 Hz), 120.5, 112.4, 108.0. NMR spectra are in accordance with the literature.[5]

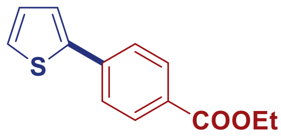

7h

Ethyl 4-(thiophen-2-yl)benzoate (7h): Following the general procedure, the reaction of 4-(ethoxycarbonyl) benzenediazonium tetrafluoroborate (**6h**) (0.23 mmol, 60.7 mg), thiophene (**5b**) (2.3 mmol, 185 μL) and **$NR_{0.17}$-COF** (1 mol% NR, 3.8 mg) in DMSO (1 mL) at room temperature under yellow LEDs, afforded the product **7h** (53%, 28.3 mg) as a white solid. **^{1}H-NMR (300 MHz, $CDCl_3$)** δ 8.05 (d, J = 8.7 Hz, 2H), 7.67 (d, J = 8.7 Hz, 2H), 7.42 (dd, J = 3.6, 1.2 Hz, 1H), 7.36 (dd, J = 5.1, 1.1 Hz, 1H), 7.11 (dd, J = 5.1, 3.7 Hz, 1H), 4.39 (q, J = 7.1 Hz, 2H), 1.41 (t, J = 7.1

Hz, 3H). **^{13}C-NMR (75 MHz, $CDCl_3$)** δ 166.4, 143.3, 138.7, 130.4, 129.3, 128.4, 126.4, 125.6, 124.6, 61.1, 14.5. NMR spectra are in accordance with the literature.[3]

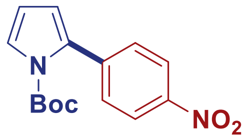

7i

***Tert*-butyl 2-(4-nitrophenyl)-1*H*-pyrrole-1-carboxylate (7i):** Following the general procedure, the reaction of 4-nitrobenzenediazonium tetrafluoroborate (**6i**) (0.23 mmol, 54.5 mg), *tert*-butyl 1*H*-pyrrole-1-carboxylate (**5c**) (0.46 mmol, 76.9 mg) and **$NR_{0.17}$-COF** (1 mol% NR, 3.8 mg) in DMSO (1 mL) at room temperature under yellow LEDs, afforded the product **7i** (57%, 37.7 mg) as a white solid. **^{1}H-NMR (300 MHz, $CDCl_3$)** δ 8.21 (d, J = 8.9 Hz, 2H), 7.51 (d, J = 9.0 Hz, 2H), 7.41 (dd, J = 3.3, 1.8 Hz, 1H), 6.32 (dd, J = 3.4, 1.8 Hz, 1H), 6.27 (t, J = 3.3 Hz, 1H), 1.43 (s, 9H). **^{13}C-NMR (75 MHz, $CDCl_3$)** δ 149.0, 146.7, 140.8, 132.9, 129.7, 124.4, 123.1, 116.7, 111.3, 84.7, 27.9. NMR spectra are in accordance with the literature.[3]

7. Procedure and Analytical Data of Compounds 9, 12 and 15

*Synthesis of phenanthrene **9**:*

In a 20 mL vial with a stirring bar, the **$NR_{0.17}$-COF** (1 mol% NR, 3.3 mg) was added under air and the system was filled with argon.[6] Then, methyl propiolate (**8**, 0.6 mmol, 54 μL) and sequentially MeOH (0.1 mL) were incorporated. [1,1'-Biphenyl]-2-diazonium tetrafluoroborate (**6j**, 0.2 mmol, 53.6 mg) was divided into 6 fractions, each fraction was dissolved in 0.1 mL of MeOH and added every hour. After 20 hours of white/yellow LEDs irradiation at room temperature, the crude was filtered through a short path of silica gel and eluting with EtOAc. The volatiles were removed under reduced pressure and the residue was purified by flash chromatography (silica; hexane/EtOAc).

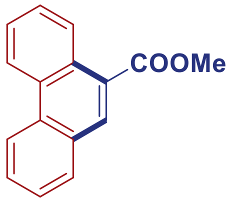

9

Methyl phenanthrene-9-carboxylate (9): Following the general procedure, the reaction afforded the product **9** (41%, 19.5 mg with white LEDs) (33%, 15.4 mg, with yellow LEDs) as a white solid. **^{1}H-NMR (700 MHz, $CDCl_3$)** δ 8.92 (dd, J = 7.9, 1.8 Hz, 1H), 8.74

(dd, J = 7.2, 2.3 Hz, 1H), 8.70 (d, J = 8.3 Hz, 1H), 8.49 (s, 1H), 7.97 (dd, J = 8.0, 1.4 Hz, 1H), 7.78 – 7.73 (m, 1H), 7.73 – 7.67 (m, 2H), 7.67 – 7.62 (m, 1H), 4.05 (s, 3H). **^{13}C-NMR (176 MHz, $CDCl_3$)** δ 168.2, 132.6, 132.3, 130.8, 130.2, 130.1, 129.2, 129.1, 127.6, 127.2, 127.1, 126.8, 126.3, 123.0, 122.8, 52.5. NMR spectra are in accordance with the literature.[6]

*Synthesis of thiocyanato-indole **12**:*

Following the general procedure described in the literature,[7] over a solution of indole (**10**, 0.5 mmol, 59.1 mg) and ammonium thiocyanate (**11**, 1.5 mmol, 116.5 mg) in THF (5 mL) in a test tube, **$NR_{0.17}$-COF** (2 mol% NR, 16.4 mg) was added. The mixture was stirred at room temperature under white LEDs irradiation. After 2 days, the crude was diluted with CH_2Cl_2, filtered through a short path of silica gel and the volatiles were removed under reduced pressure. The product **12** was obtained analytically pure after purification by flash chromatography using silica gel and hexane/EtOAc as eluent.

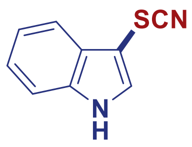

12

3-Thiocyanato-1*H*-indole (12): Following the general procedure, the reaction afforded the product **12** (71%, 61.5 mg with white LEDs) (66%, 57.7 mg, with yellow LEDs) as a pale white solid. **^{1}H-NMR (300 MHz, $CDCl_3$)** δ 8.85 (br, 1H), 7.86 – 7.75 (m, 1H), 7.46 – 7.37 (m, 2H), 7.36 – 7.27 (m, 2H). **^{13}C-NMR (75 MHz, $CDCl_3$)** δ 136.1, 131.3, 127.7, 123.9, 121.9, 118. 7, 112.4, 112.3, 91.7. NMR spectra are in accordance with the literature.[7]

*Synthesis of formylated indole **15**:*

A 20 mL vial was charged with 1-methyl-1*H*-indole (**13**, 0.2 mmol, 25 µL), TMDA (**14**, 0.4 mmol, 61 µL), **$NR_{0.17}$-COF** (5 mol% NR, 16.5 mg), KI (0.8 mmol, 134.1 mg), MeCN (1.0 mL) and H_2O (0.2 mL).[8] The system was charged with an O_2 balloon and placed in a clear oil heated at 60 ºC. After 48 hours of white LEDs irradiation, the reaction mixture was quenched with water and extracted 3 times with EtOAc. The combined organic phases were dried over anhydrous $MgSO_4$, filtered and concentrated under reduced pressure. The crude was finally purified by silica gel chromatography using

hexane/EtOAc as eluent.

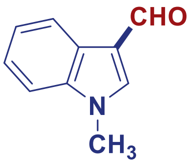

15

1-Methyl-1*H*-indole-3-carbaldehyde (15): Following the general procedure, the reaction afforded the product **15** (63%, 19.8 mg with white LEDs) (51%, 16.3 mg with yellow LEDs) as a pale white solid. **^{1}H-NMR (300 MHz, $CDCl_3$)** 10.00 (s, 1H), 8.35 – 8.26 (m, 1H), 7.68 (s, 1H), 7.40 – 7.30 (m, 3H), 3.88 (s, 3H). **^{13}C-NMR (75 MHz, $CDCl_3$)** δ 184.5, 139.3, 138.0, 125.4, 124.2, 123.1, 122.2, 118.2, 110.0, 33.8. NMR spectra are in accordance with the literature.[8]

8. Scale-up Experiment

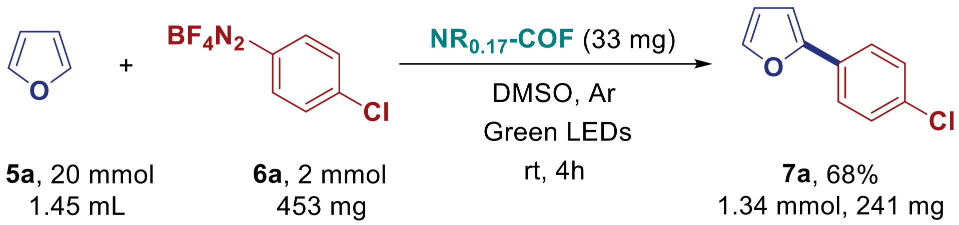

Following the general procedure for the synthesis of compounds **7**, 20 mL glass vial with a stirring bar, 4-chlorobenzenediazonium tetrafluoroborate **6a** (2.0 mmol, 452.7 mg) and **$NR_{0.17}$-COF** (1 mol%, 33.1 mg) were added under air and, afterwards, the reaction vessel with the reflux system was filled with argon. Then, furane **5a** (20 mmol, 1.45 mL) and sequentially deoxygenated anhydrous DMSO (8.7 mL) were added under an argon atmosphere, and the mixture was stirred at room temperature under green LEDs irradiation for 4 hours. After this time, the reaction mixture was transferred to a separating funnel, diluted with EtOAc and washed with water. The aqueous layer was washed three times with EtOAc. The organic layers were combined, dried with anhydrous $MgSO_4$, filtered and concentrated under reduced pressure. Finally, the analytically pure product **7a** was obtained in 68% (241.2 mg) by flash chromatography (silica gel; hexane/EtOAc).

9. Recyclability Experiment

In a 20 mL glass vial with a stirring bar, 4-chlorobenzenediazonium tetrafluoroborate **6a** (0.23 mmol, 52.1 mg) and **NR$_{0.17}$-COF** (1 mol% NR, 3.8 mg) were added under air. Then, the reaction vessel was filled with argon. Afterwards, furane **5a** (2.3 mmol, 170 µL) and sequentially deoxygenated anhydrous DMSO (1.0 mL) were added under an argon atmosphere, and the mixture was stirred at room temperature under green LEDs irradiation for 4 hours. After this time, the glass vial with the crude was centrifuged and washed three times with THF/Hexane (5:1). The crude extracted was concentrated over reduced pressure and the residue was transferred to a separating funnel, diluted with ethyl acetate and washed with water. The aqueous layer was washed three times with ethyl acetate. The organic layers were combined, dried with anhydrous $MgSO_4$, filtered and concentrated under reduced pressure. After this time, the crude was analyzed by NMR to determine the yield using nitromethane as internal standard. Meanwhile, the catalyst recovered as a red powder was dried under vacuum overnight before the next catalytic run. Using **NR$_{0.17}$-COF** as catalyst, 6 catalytic runs were performed, producing 165.7 mg (0.92 mmol) of 2-(4-chlorophenyl)furan **7a**.

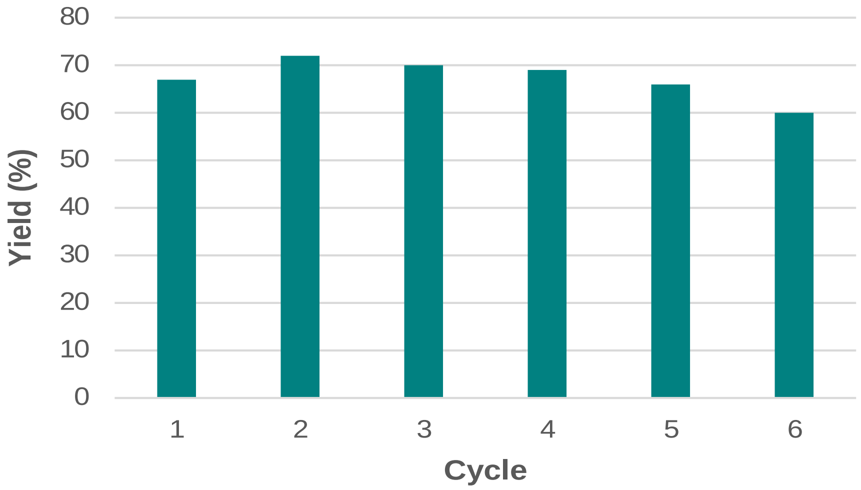

Figure S19. Recyclability of **NR$_{0.17}$-COF**.

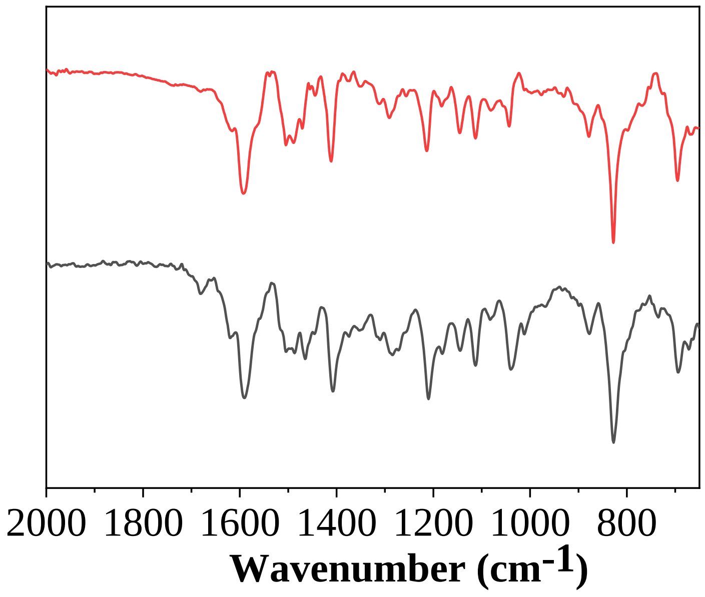

Figure S20. Comparison of FTIR spectra of **NR$_{0.17}$-COF** before and after catalysis.

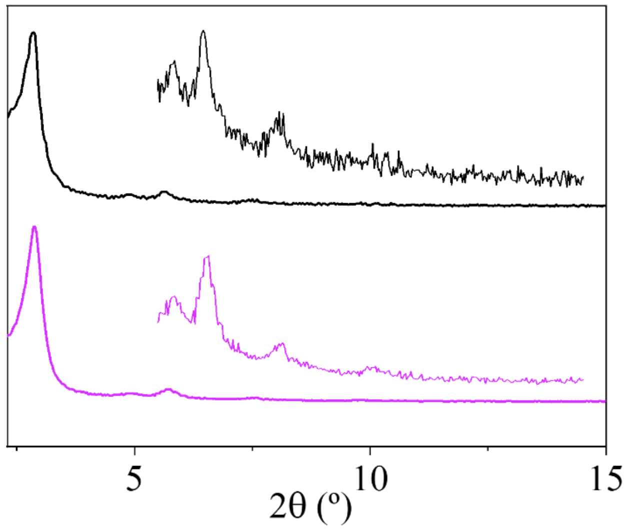

Figure S21. Comparison of PXRD patterns of **NR$_{0.17}$-COF** before and after catalysis.

10. Kinetic Experiments

Following the general procedure for the synthesis of compounds **7**, 20 mL glass vial with a stirring bar, 4-chlorobenzenediazonium tetrafluoroborate **6a** (0.57 mmol, 130.5 mg) and **NR$_{0.17}$-COF** (1 mol% NR, 9.5 mg) or **NR-Alk** (1 mol%, 2.1 mg) or **Azide$_{0.17}$-COF** (1 mol% N_3, 9.5 mg) or no catalyst were added under air and, afterwards, the

reaction vessel was filled with argon. Then, furane **5a** (5.7 mmol, 420 µL) and sequentially deoxygenated anhydrous deuterated DMSO-d_6 (2.5 mL) were added under an argon atmosphere, and the mixture was stirred at room temperature under green LEDs. Once the reaction started, an aliquot was taken at 5, 10, 15, 20, 30, 45, 60, 120 and 240 minutes from the reaction and was analyzed by ^{1}H-NMR to determine the yield using nitromethane as internal standard.

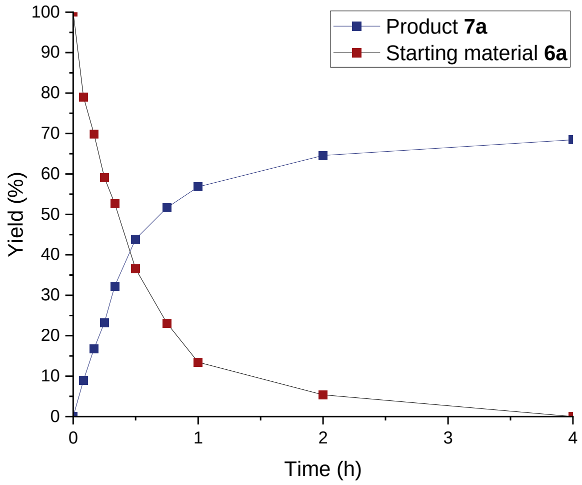

Figure S22. Kinetic profile for the consumption of **6a**/production of **7a** using **NR$_{0.17}$-COF** as catalyst.

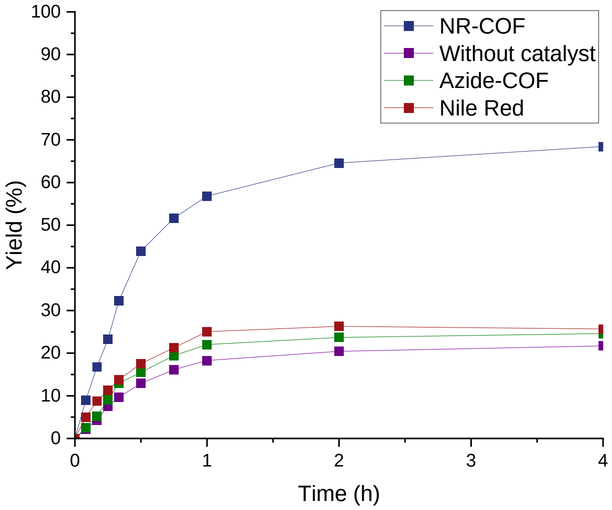

Figure S23. Kinetic profiles for the standard reaction using different catalysts.

11. Radical Capturing Experiment

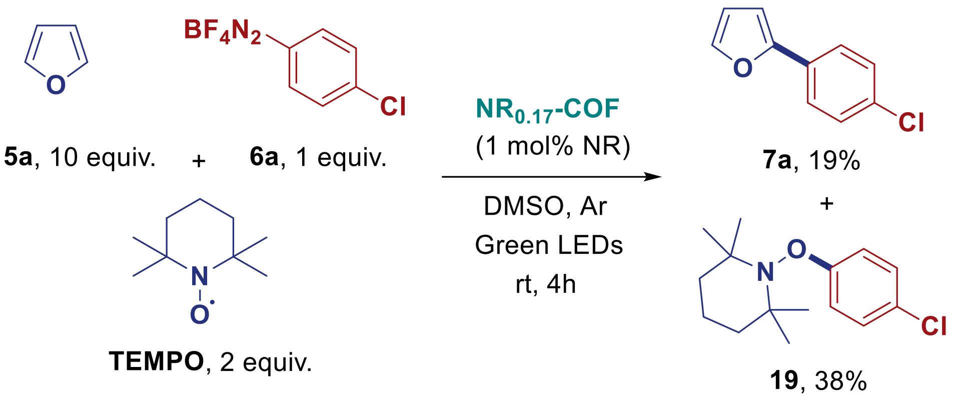

Following the general procedure for the synthesis of compounds **7**, 20 mL glass vial with a stirring bar, 4-chlorobenzenediazonium tetrafluoroborate **6a** (0.23 mmol, 52.1 mg), **$NR_{0.17}$-COF** (1 mol% NR, 3.8 mg) and **TEMPO** (0.46 mmol, 72.6 mg) were added under air and, afterwards, the reaction vessel was filled with argon. Then, furane **5a** (2.3 mmol, 170 μL) and sequentially deoxygenated anhydrous DMSO (1.0 mL) were added under an argon atmosphere, and the mixture was stirred at room temperature under green LEDs irradiation for 4 hours. After this time, the reaction mixture was transferred to a separating funnel, diluted with EtOAc and washed with water. The aqueous layer was washed three times with EtOAc. The organic layers were combined, dried with anhydrous $MgSO_4$, filtered and concentrated under reduced pressure. Finally, after purification by flash chromatography (silica gel; hexane/EtOAc) the product **7a** (19%, 7.9 mg) was obtained in combination with **19**.

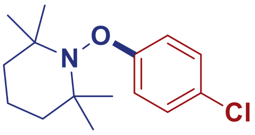

19

1-(4-Chlorophenoxy)-2,2,6,6-tetramethylpiperidine (19): Following the procedure, the reaction afforded the product **19** (38%, 23.2 mg) as a pale white solid. **^{1}H-NMR (300 MHz, $CDCl_3$)** δ 7.16 (d, J = 9.4 Hz, 2H), 7.11 (d, J = 9.3 Hz, 2H), 1.70 – 1.36 (m, 6H), 1.22 (s, 6H), 0.99 (s, 6H). **^{13}C-NMR (75 MHz, $CDCl_3$)** δ 162.4, 128.7, 124.5, 115.3, 60.6, 39.9, 32.6, 20.6, 17.1. NMR spectra are in accordance with the literature.[9]

12. "Light/dark" Experiment

Following the general procedure for the synthesis of compounds **7**, 20 mL glass vial with a stirring bar, 4-chlorobenzenediazonium tetrafluoroborate **6a** (0.46 mmol, 104.1 mg) and **$NR_{0.17}$-COF** (1 mol% NR, 7.6 mg) were added under air and, afterwards, the reaction vessel was filled with argon. Then, furane **5a** (4.6 mmol, 330 μL) and sequentially deoxygenated anhydrous deuterated DMSO-d_6 (2.0 mL) were added under an argon atmosphere. The mixture was stirred at room temperature and alternating periods of 15 minutes of irradiation under green LEDs and darkness have been performed. At the end of each period, an aliquot was taken and was analyzed by ^{1}H-NMR to determine the yield using nitromethane as internal standard.

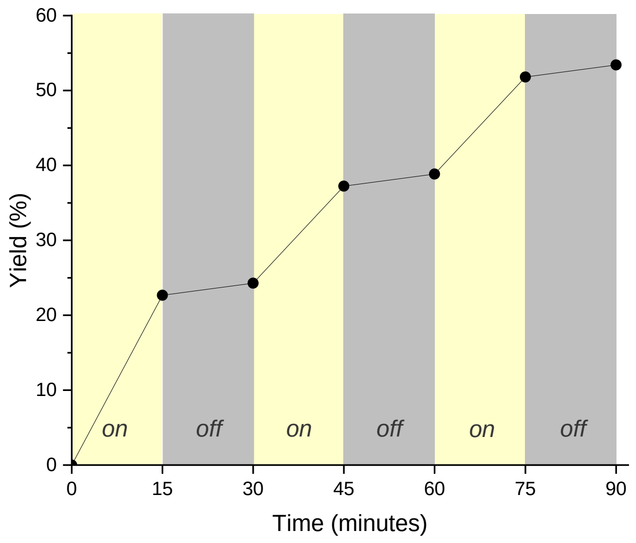

Figure S24. "Light/dark" experiment for the standard reaction using **$NR_{0.17}$-COF** as catalyst.

13. Electrochemical Measurements

Electrochemical measurements were performed with a potentiostat Autolab PGSTAT302N (EcoChemie, NL) using the software package NOVA 2.16. A three-electrode set-up using a homemade single-compartment electrochemical cell was employed. Glassy carbon (GC) from CH Instruments were used as working electrodes and Pt wire as counter electrode. Specific calomel electrode (1 M LiCl for organic media from Radiometer Analytical) was used as reference electrode. Electrochemical

measurements have been carried out in 0.1 M TBAP (tetrabutylammonium perchlorate)/ acetonitrile solution previously deoxygenated using argon.

GC working electrodes were modified by drop-casting with 5 µL of the corresponding suspension. The suspensions were prepared mixing 1 mg of the **$NR_{0.17}$-COF** or 1 mg of **$Azide_{0.17}$-COF** with 1 mg of carbon black (CB) super P in 1 mL of a water solution containing 20 % ethanol and 0.05 % Nafion®. After the modified electrode was dried at 70 ºC during 10 min, the electrodes were employed.

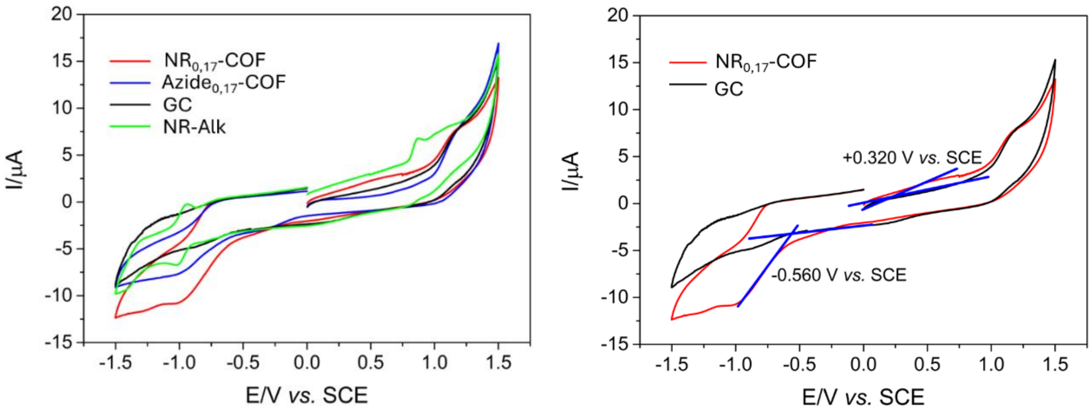

Figure S25. Cyclic voltammograms recorded at modified glassy carbon with carbon black (black) and with **$NR_{0.17}$-COF/**carbon black (red) and **$Azide_{0.17}$-COF/**carbon black (blue) in 0.1 M TBAP/acetonitrile solution in the absence of O_2. Cyclic voltammograms recorded at modified glassy carbon with carbon black in the presence of 1 mg/mL of **NR-Alk** (green) in 0.1 M TBAP/acetonitrile solution in the absence of O_2.

$$E_{LUMO}=(E_{red}-E_{ferrocene})+4.8\ eV = 3.837\ eV$$

$$E_{HOMO}=(E_{ox}-E_{ferrocene})+4.8\ eV = 4.717\ eV$$

$$E_{gap} = 0.880\ eV$$

$E_{ferrocene}$ *vs.* SCE = -0.403 V`[10]

14. Comparison of $NR_{0.17}$-COF with Other Photocatalysts

The following table, Table S2, shows the comparison of photocatalytic activity of $NR_{0.17}$-COF with that of other homogeneous and heterogeneous photocatalysts previously reported for the arylation of heterocycles with diazonium salts.

Table S2. Comparison of **$NR_{0.17}$-COF** with other photocatalysts employed in the arylation of heteroarenes using diazonium salts.

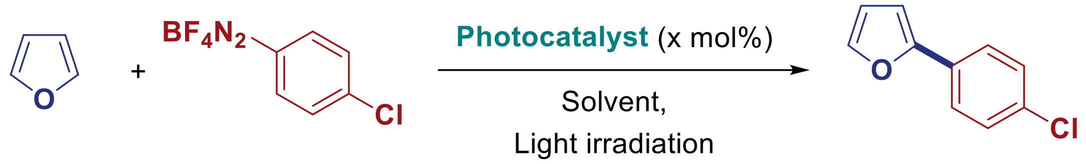

5a (x equiv.) **6a** (1 equiv.) **7a**

Entry	Photocatalyst	Equiv. of 5a	Solvent	T (ºC)	Time (h)	Light (LEDs)	Yield of 7a (%)
1[11]	Eosin Y	10	DMSO	20	2	Green	74
2[12]	$AcrH_2$	20	CH_3CN	rt	12	Blue	69
3[13]	Bismuthene	20	DMSO	25	2	Indoor	75
4[14]	Pnictogen bismuth	20	DMSO	rt	2	Blue	79
5[15]	Black phosphorus	10	DMSO	25	2	150 W metal halide lamp	76
6[16]	$CuInS_2$/ K-C_3N_4	10	H_2O	rt	8	Blue	81
This work	**$NR_{0.17}$-COF**	10	DMSO	rt	4	Yellow	70

DMSO = dimethylsulfoxide. NR = Nile Red.

15. References

(1) Yang, Z.; He, Y.; Lee, J. H.; Chae, W.-S.; Ren, W. X.; Lee, J. H.; Kang, C.; Kim, S. A Nile Red/BODIPY-based bimodal probe sensitive to changes in the micropolarity and microviscosity of the endoplasmic reticulum. *Chem. Commun.* **2014**, *50*, 11672-11675.

(2) Börgardts, M.; Verlinden, K.; Neidhardt, M.; Wöhrle, T.; Herbst, A.; Laschat, S.; Janiak, C.; Müller, T. J. J. Synthesis and optical properties of covalently bound Nile Red in mesoporous silica hybrids–comparison of dye distribution of materials prepared by facile grafting and by co-condensation routes. *RSC Advances* **2016**, *6*, 6209-6222.

(3) Hari, D.P.; Schroll, P.; König, B. Metal-Free, Visible-Light-Mediated Direct C–H Arylation of Heteroarenes with Aryl Diazonium Salts. *J. Am. Chem. Soc.* **2012**, *134*, 2958-2961.

(4) Rybicka-Jasińska, K.; König, B.; Gryko, D. Porphyrin-Catalyzed Photochemical C–H

Arylation of Heteroarenes. *Eur. J. Org. Chem.* **2017**, 2104–2107.

(5) Chen, Y.-L.; Sun, J.-Q.; Wei, X.; Wong, W.-Y.; Lee, A. W. M. Generation of Synthetic Equivalents of Benzdiynes from Benzobisoxadisiloles. *J. Org. Chem.* **2005**, *70*, 3525-3529.

(6) Xiao, T.; Dong, X.; Tang, Y.; Zhou, L. Phenanthrene Synthesis by Eosin Y-Catalyzed, Visible Light-Induced [4+2]Benzannulation of Biaryldiazonium Salts with Alkynes. *Adv. Synth. Catal.* **2012**, *354*, 3195-3199.

(7) Fan, W.; Yang, Q.; Xu, F.; Li, P. A Visible-Light-Promoted Aerobic Metal-Free C-3 Thiocyanation of Indoles. *J. Org. Chem.* **2014**, *79*, 10588-10592.

(8) Li, X.; Gu, X.; Li, Y.; Li, P. Aerobic Transition-Metal-Free Visible-Light Photoredox Indole C-3 Formylation Reaction. *ACS Catal.* **2014**, *4*, 1897-1900.

(9) Zhang, N.; Quan, Z.-J.; Zhang, Z.; Da, Y.-X.; Wang, X.-C. Synthesis of stilbene derivatives via visible-light-induced cross-coupling of aryl diazonium salts with nitroalkenes using -NO_2 as a leaving group. *Chem. Commun.* **2016**, *52*, 14234-14237.

(10) Pomrnerehne, J.; Vestweber, H.; Guss, W.; Mahrt, R. E.; Bassler, H.;Porsch, M.; Daub, J. Efficient Two Layer LEDs on a Polymer Blend Basis. *Adv. Mater.* **1995**, *7*, 551-554.

(11) Hari, D. P.; Schroll, P.; König, B. Metal-Free, Visible-Light-Mediated Direct C–H Arylation of Heteroarenes with Aryl Diazonium Salts. *J. Am. Chem. Soc.* **2012**, *134*, 2958-2961.

(12) Feng, Y.-S.; Bu, X.-S.; Huang, B.; Rong, C.; Dai, J.-J.; Xu, J.; Xu, H.-J. NADH Coenzyme Model Compound as Photocatalyst for the Direct Arylation of (Hetero)arenes. *Tetrahedron Lett.* **2017**, *58*, 1939-1942.

(13) Ozer, M. S.; Eroglu, Z.; Yalin, A. S.; Kiliç, M.; Rothlisberger, U.; Metin, O. Bismuthene as a versatile photocatalyst operating under variable conditions for the photoredox C–H bond functionalization. *Appl. Catal. B* **2022**, *304*, 120957.

(14) Huang, Z.; Zhu, J.; Dong, Q.; Huang, Q.; Wang, M.; Zhu, Y.; Huang, W.; Zi, Y. Photoredox C–H Arylation of Heteroarenes by High-Efficiency Bismuth Quantum Dots. *Tetrahedron Lett.* **2023**, *125*, 154667.

(15) Kalay, E.; Küçükkeçeci, H.; Kilic, H.; Metin, Ö. Phosphorus as a Metal-Free, Visible-Light-Active Heterogeneous Photoredox Catalyst for the Direct C–H Arylation of Heteroarenes with Aryl Diazonium Salts. *Chem. Commun.* **2020**, *56*, 5901-5904.

(16) Liu, Q.-H.; Kang, S.-L.; Cui, Z.-S.; Liu, Y.-H.; Zhang, M.; Zhang, Z.-H. Visible Light-Driven C–H Arylation of Heteroarenes with Aryl Diazonium Salts in Water Catalyzed by Z-Scheme $CuInS_2$/K-C_3N_4 Heterojunction. *Green Chem.* **2024**, *26*, 4803-4810.

16. NMR Spectra of Products

^{1}H-NMR (300 MHz, $CDCl_3$)

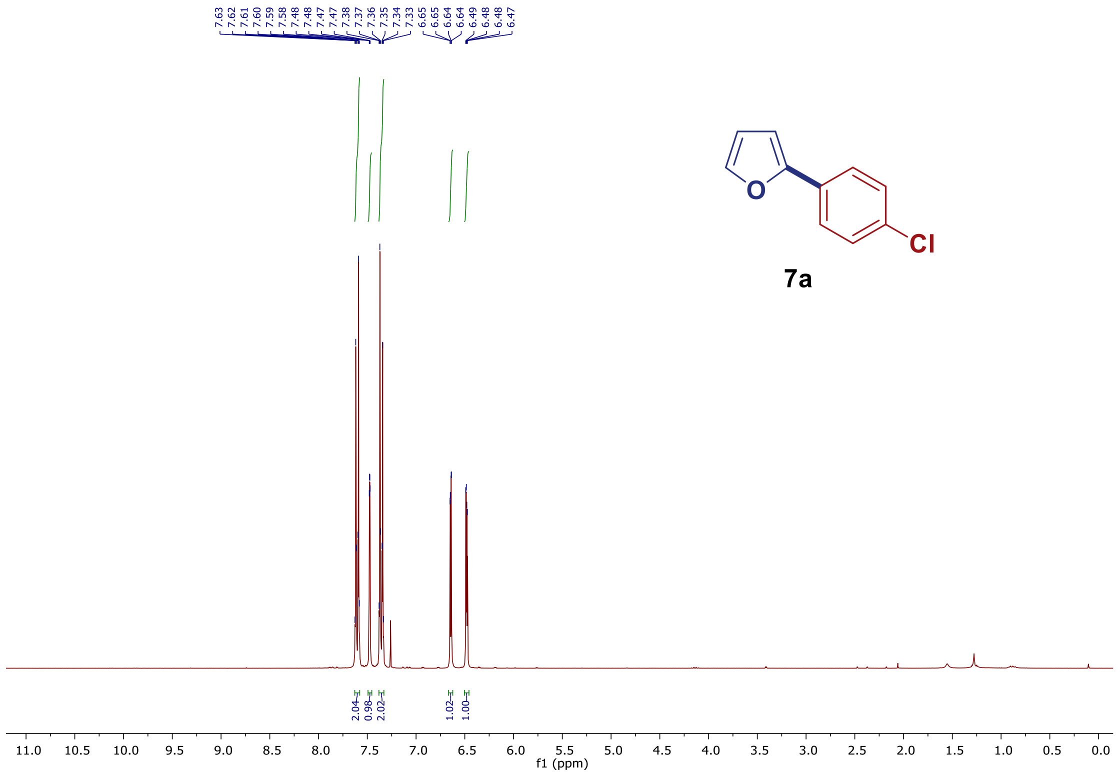

^{13}C-NMR (75 MHz, $CDCl_3$)

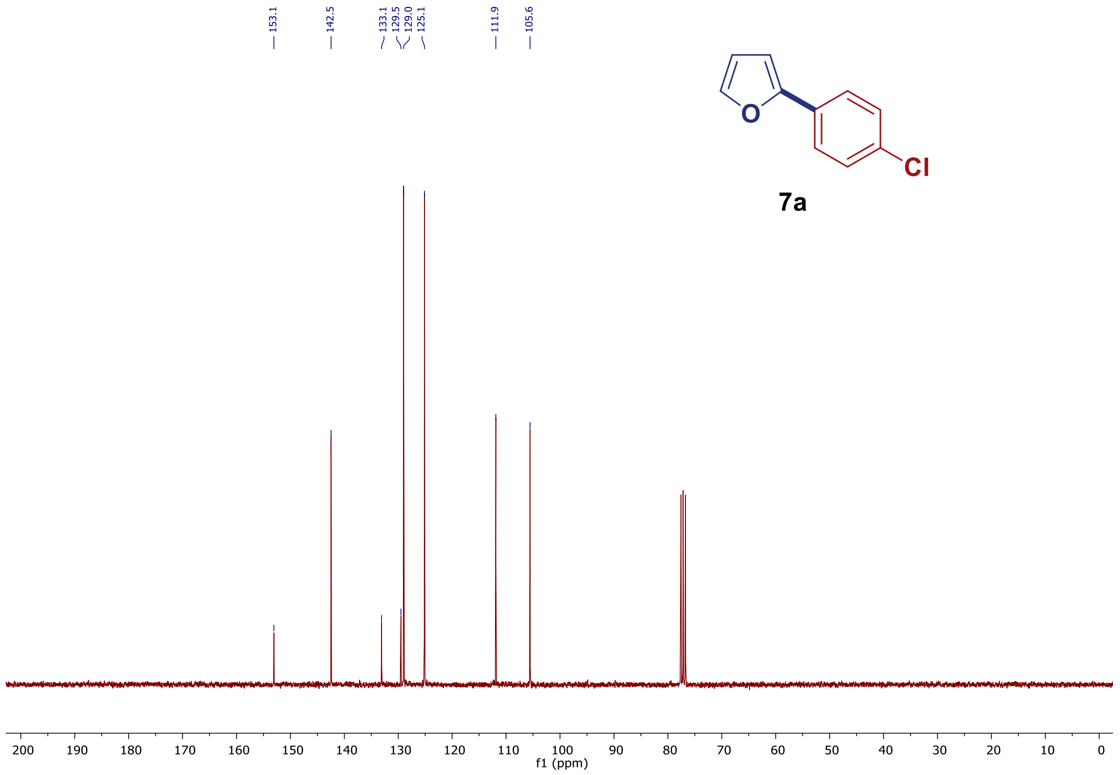

^{1}H-NMR (300 MHz, $CDCl_3$)

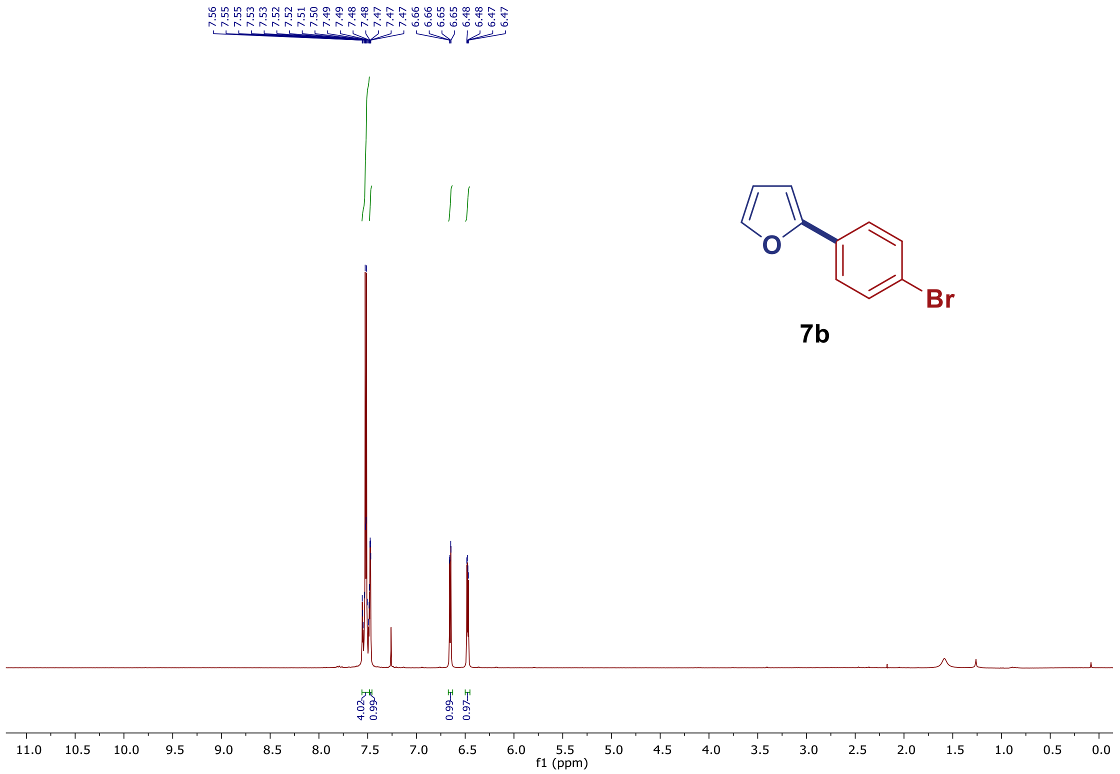

^{13}C-NMR (75 MHz, $CDCl_3$)

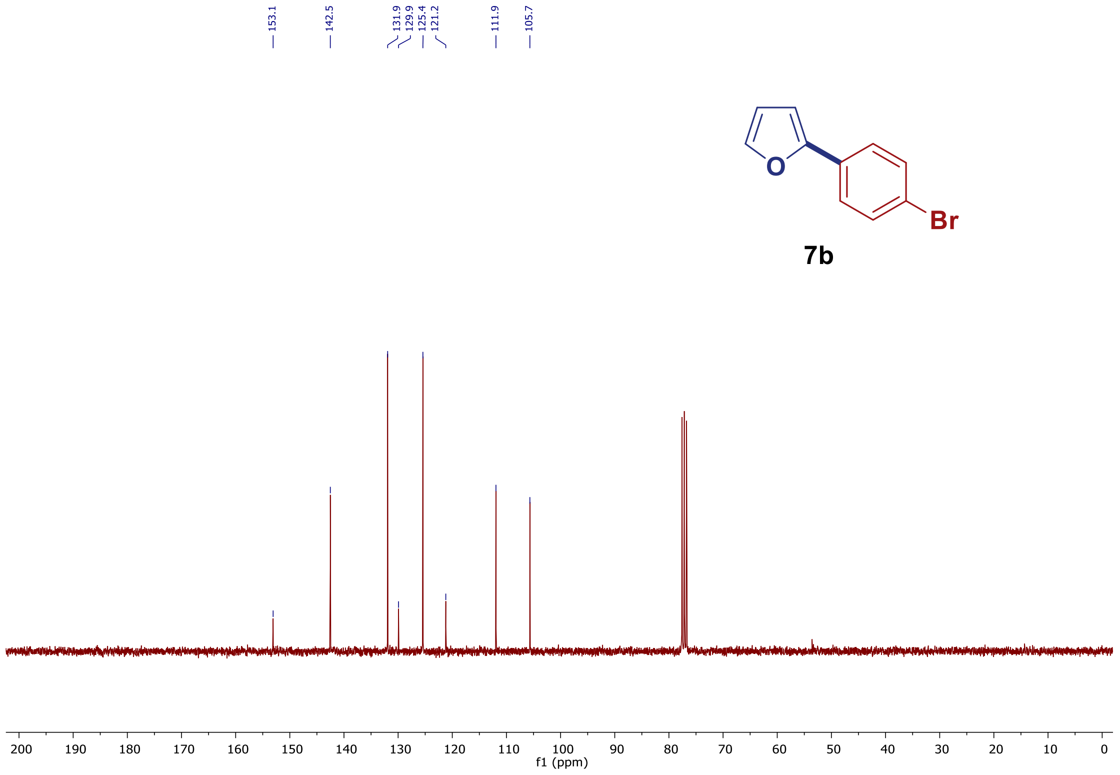

^{1}H-NMR (300 MHz, $CDCl_3$)

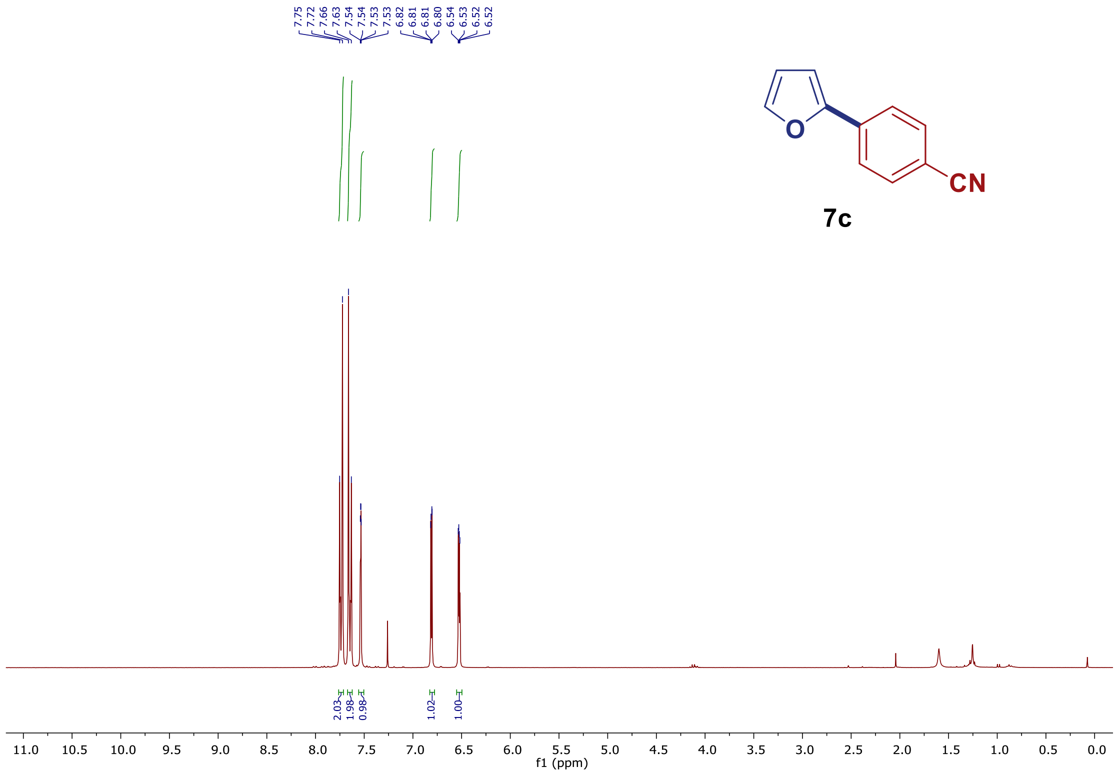

^{13}C-NMR (75 MHz, $CDCl_3$)

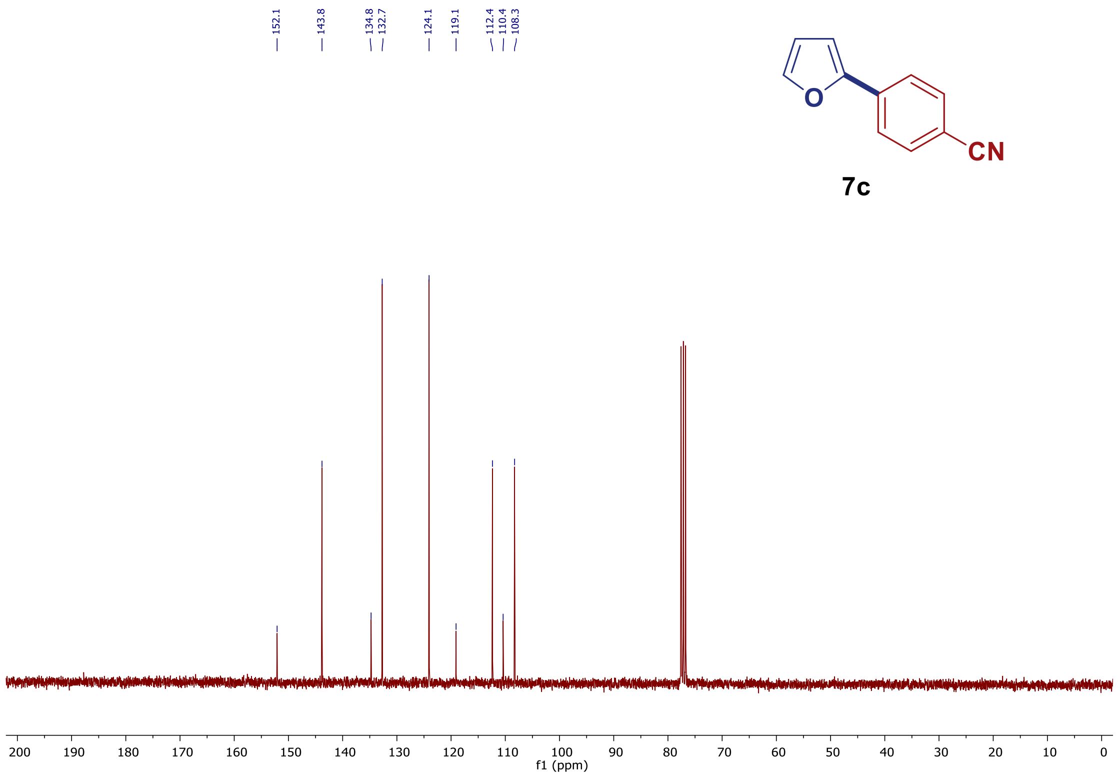

^{1}H-NMR (300 MHz, $CDCl_3$)

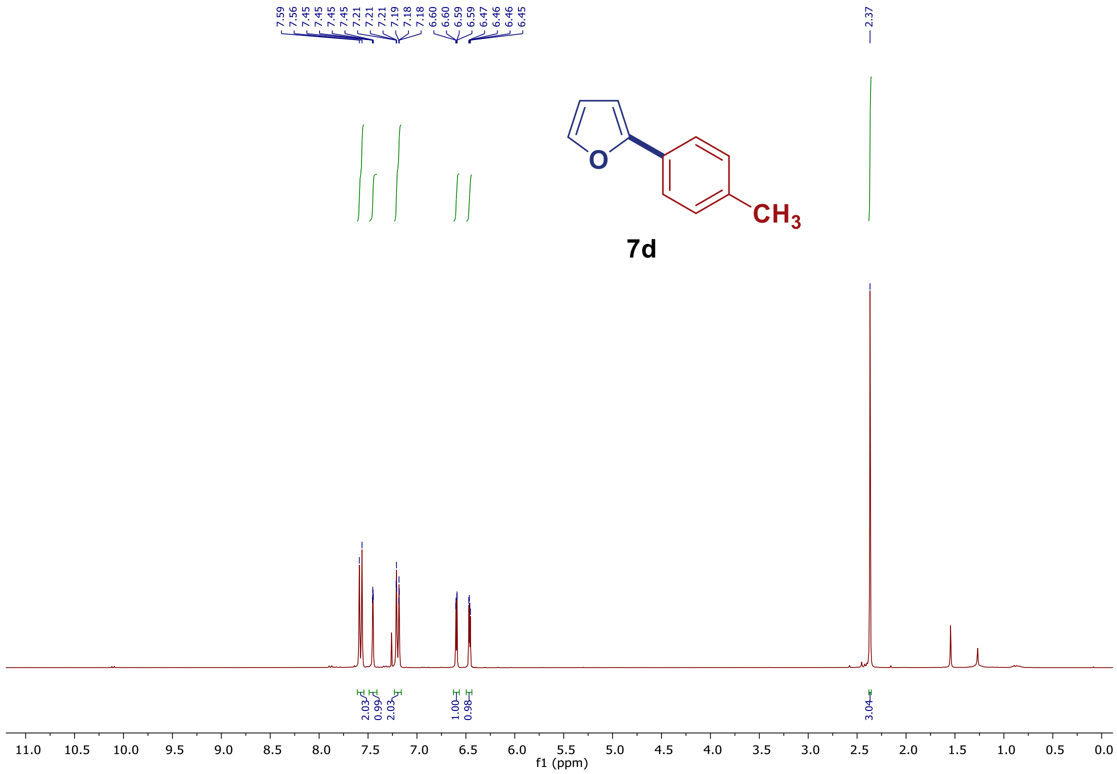

^{13}C-NMR (75 MHz, $CDCl_3$)

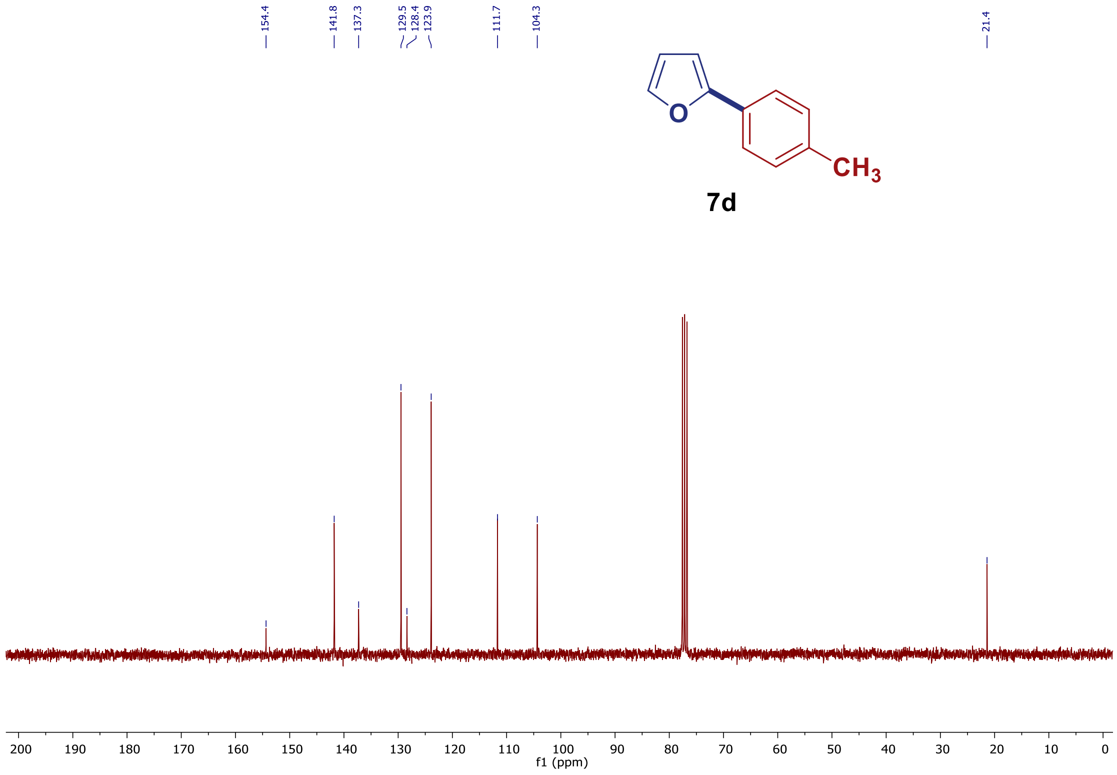

^{1}H-NMR (300 MHz, $CDCl_3$)

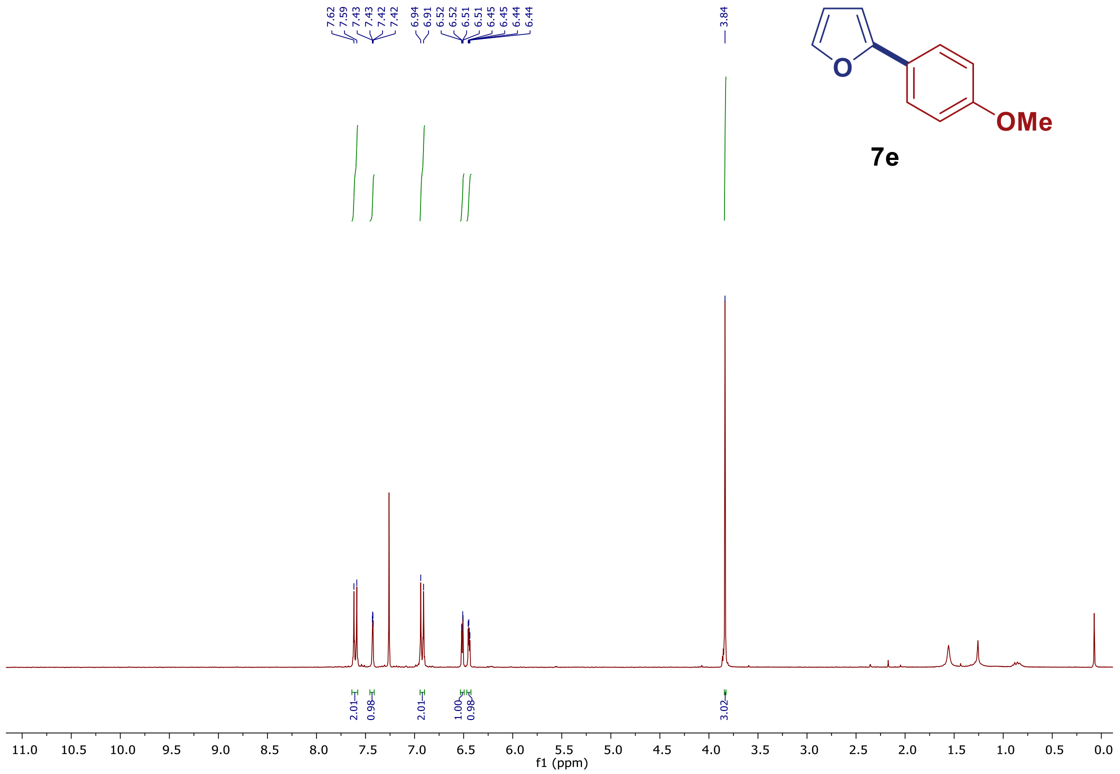

^{13}C-NMR (75 MHz, $CDCl_3$)

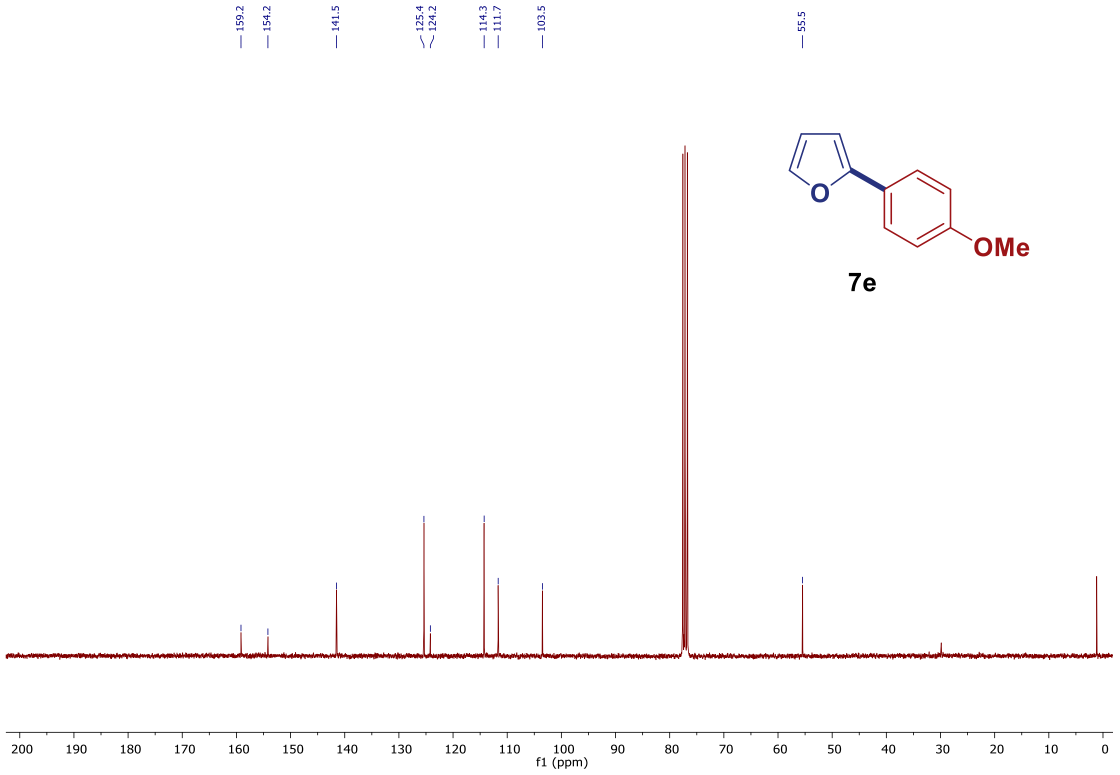

^{1}H-NMR (300 MHz, $CDCl_3$)

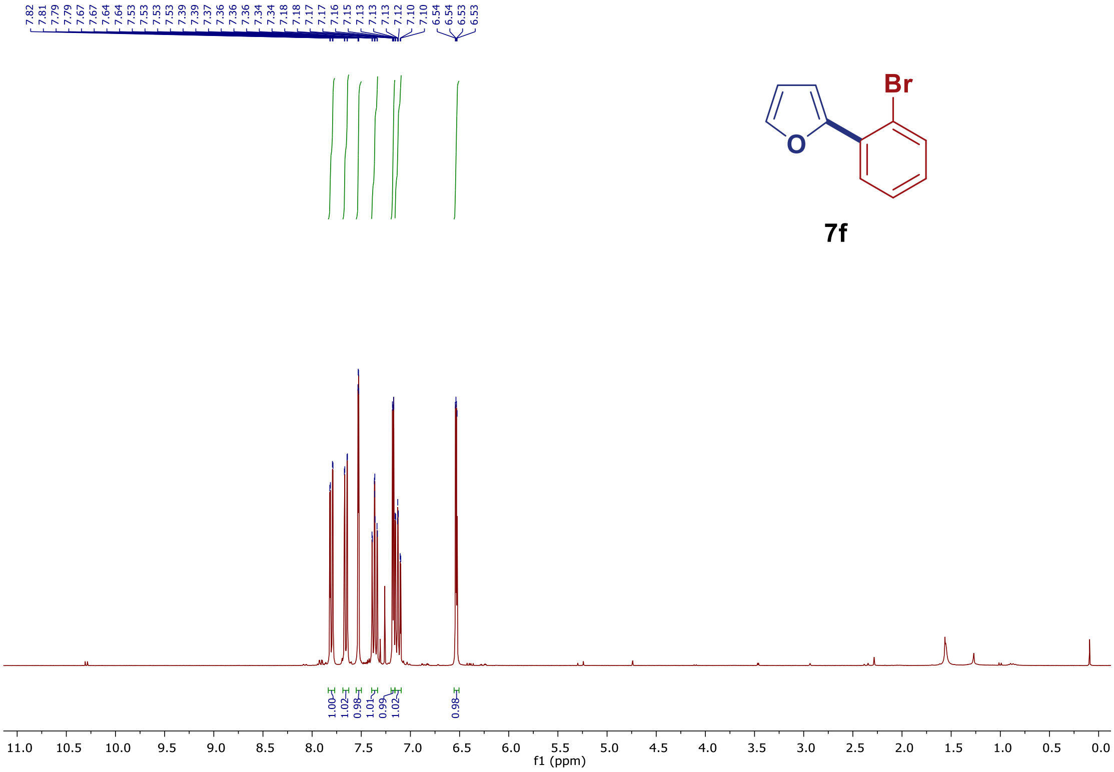

7f

^{13}C-NMR (75 MHz, $CDCl_3$)

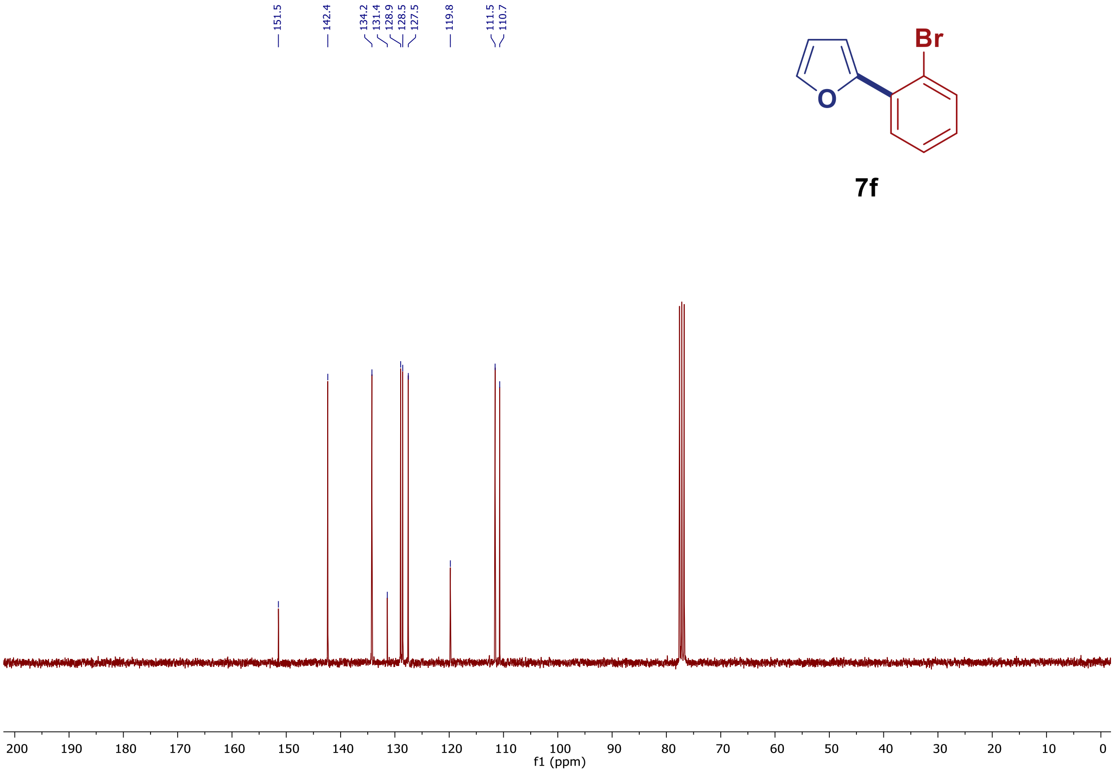

7f

^{1}H-NMR (300 MHz, $CDCl_3$)

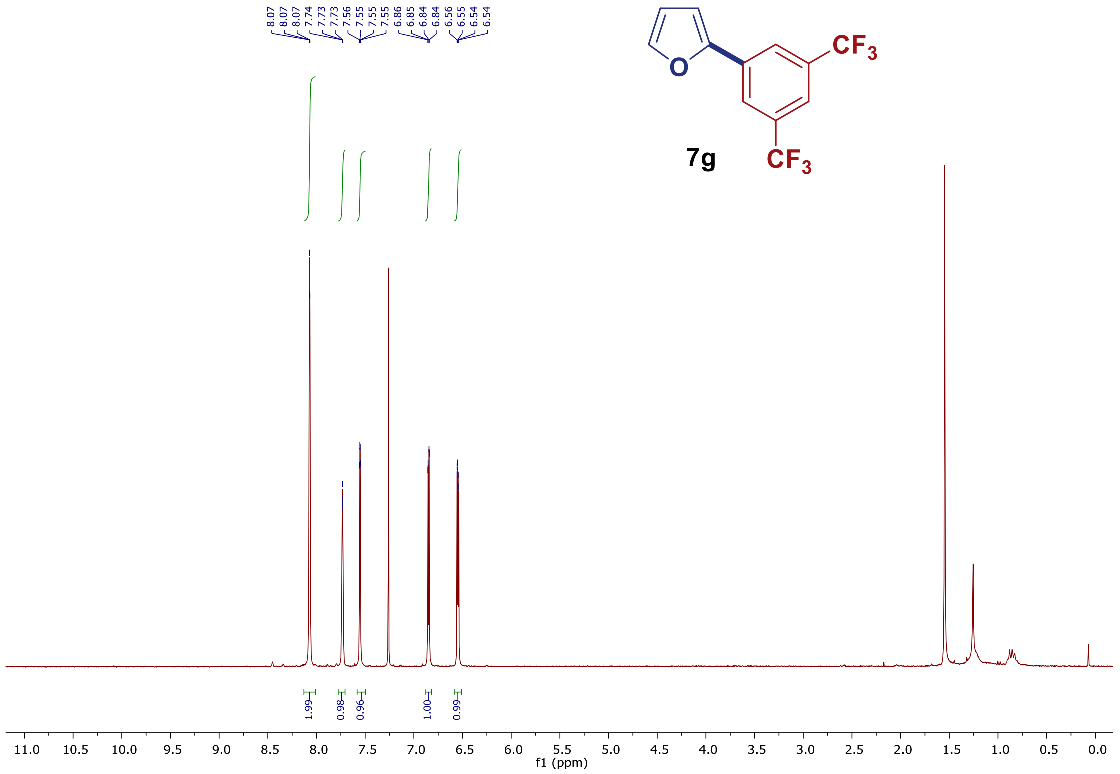

^{13}C-NMR (75 MHz, $CDCl_3$)

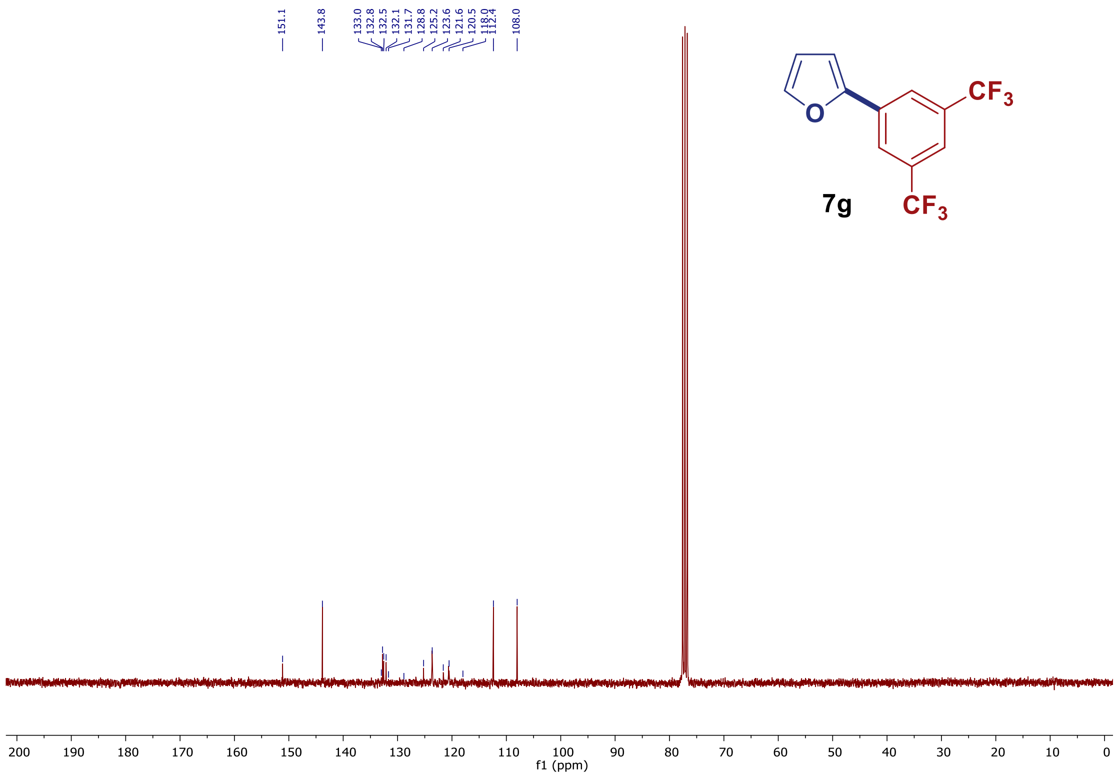

1H-NMR (300 MHz, $CDCl_3$)

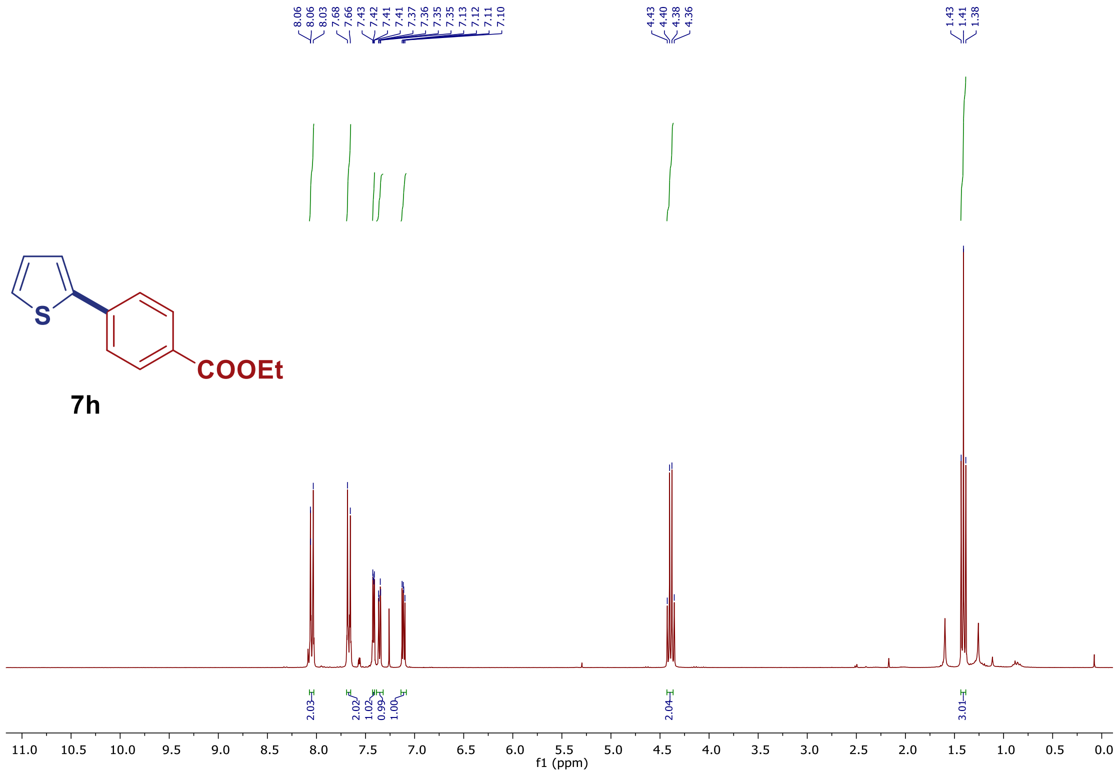

13C-NMR (75 MHz, $CDCl_3$)

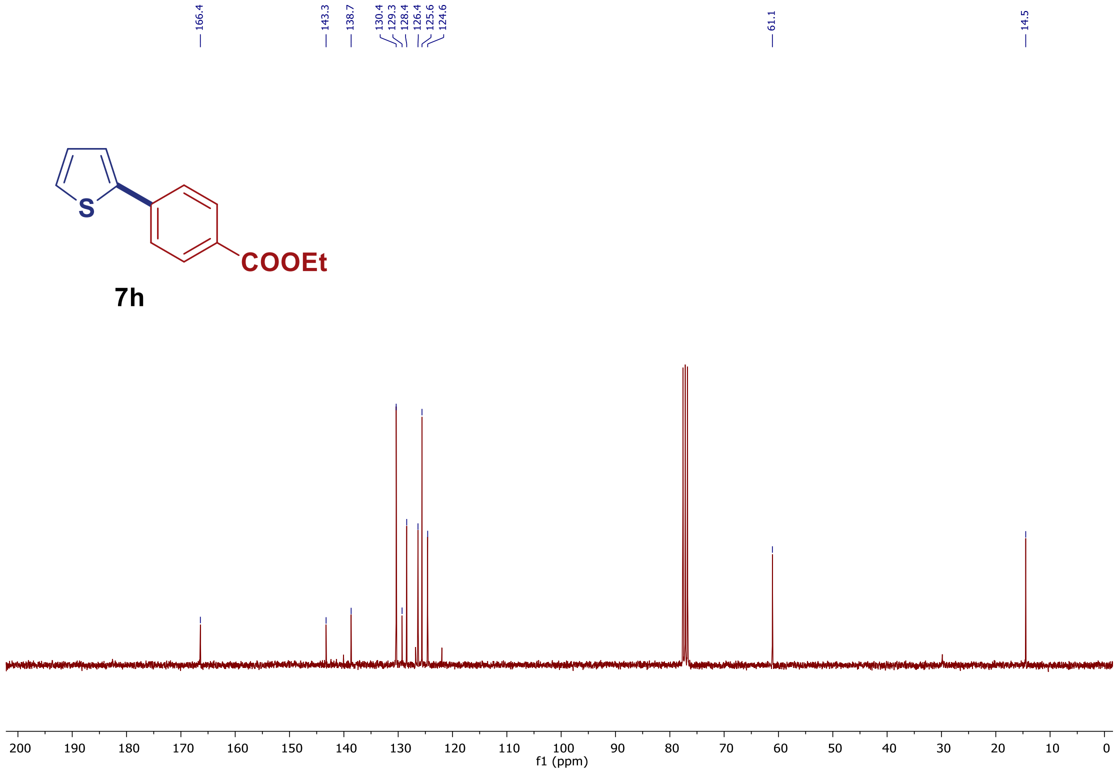

^{1}H-NMR (300 MHz, $CDCl_3$)

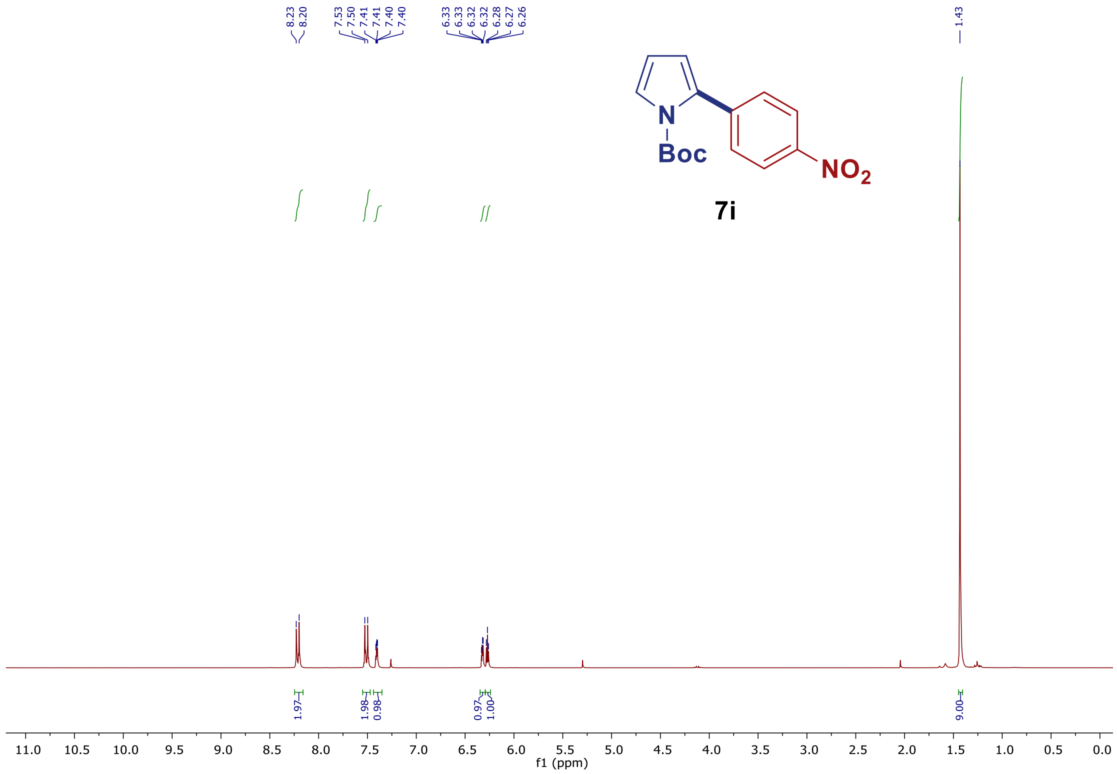

^{13}C-NMR (75 MHz, $CDCl_3$)

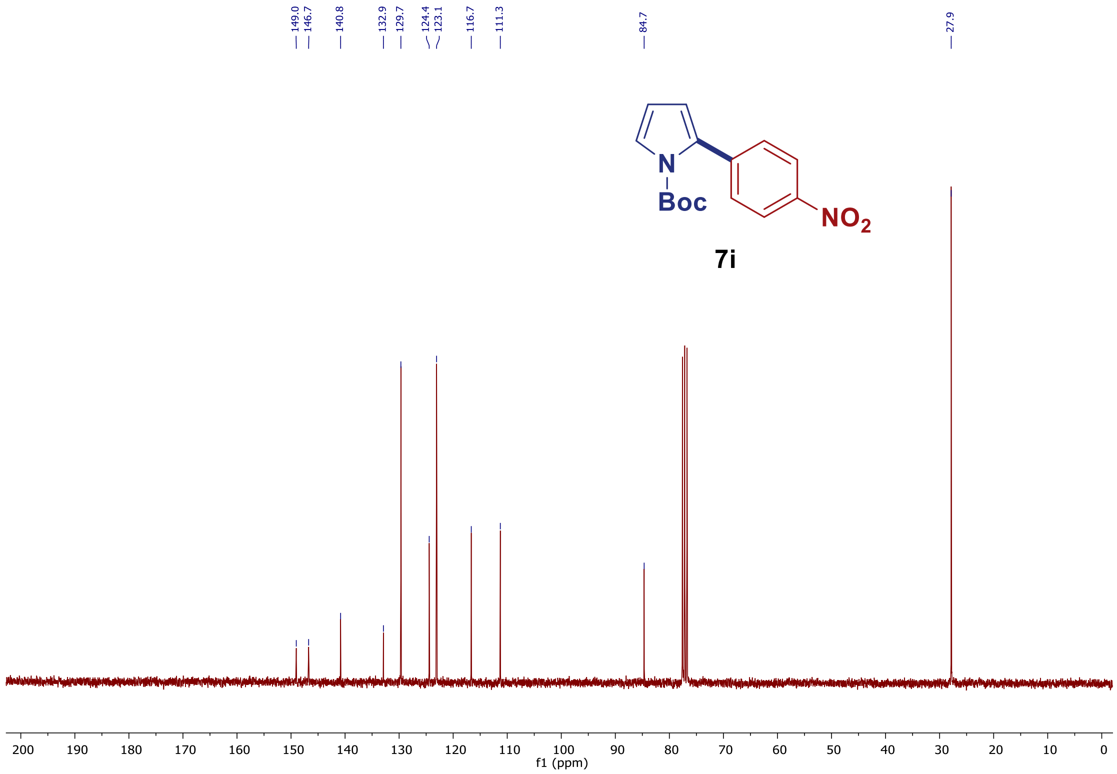

^{1}H-NMR (700 MHz, $CDCl_3$)

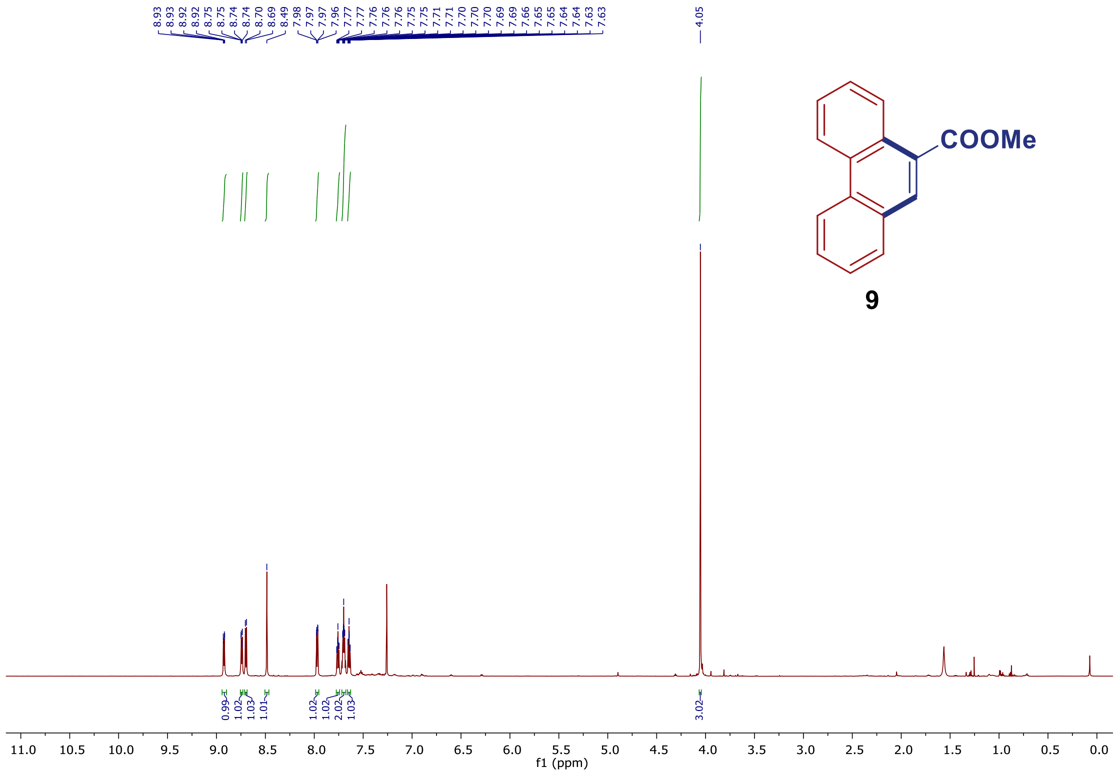

^{13}C-NMR (176 MHz, $CDCl_3$)

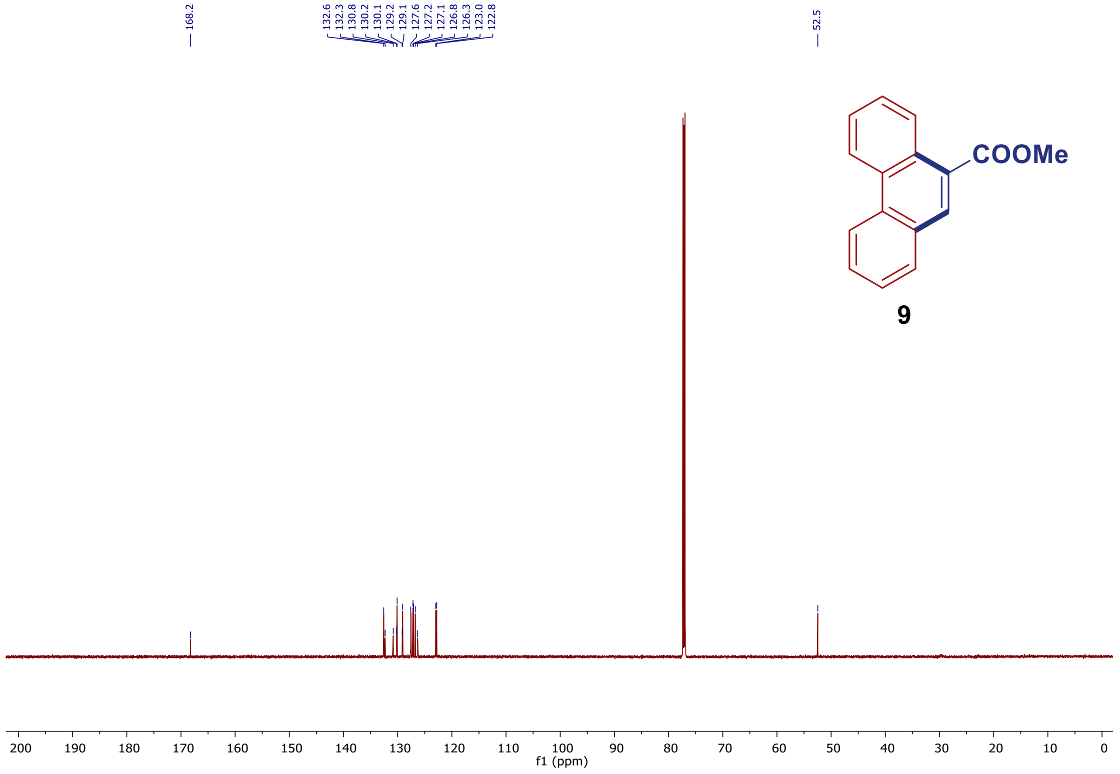

^{1}H-NMR (300 MHz, $CDCl_3$)

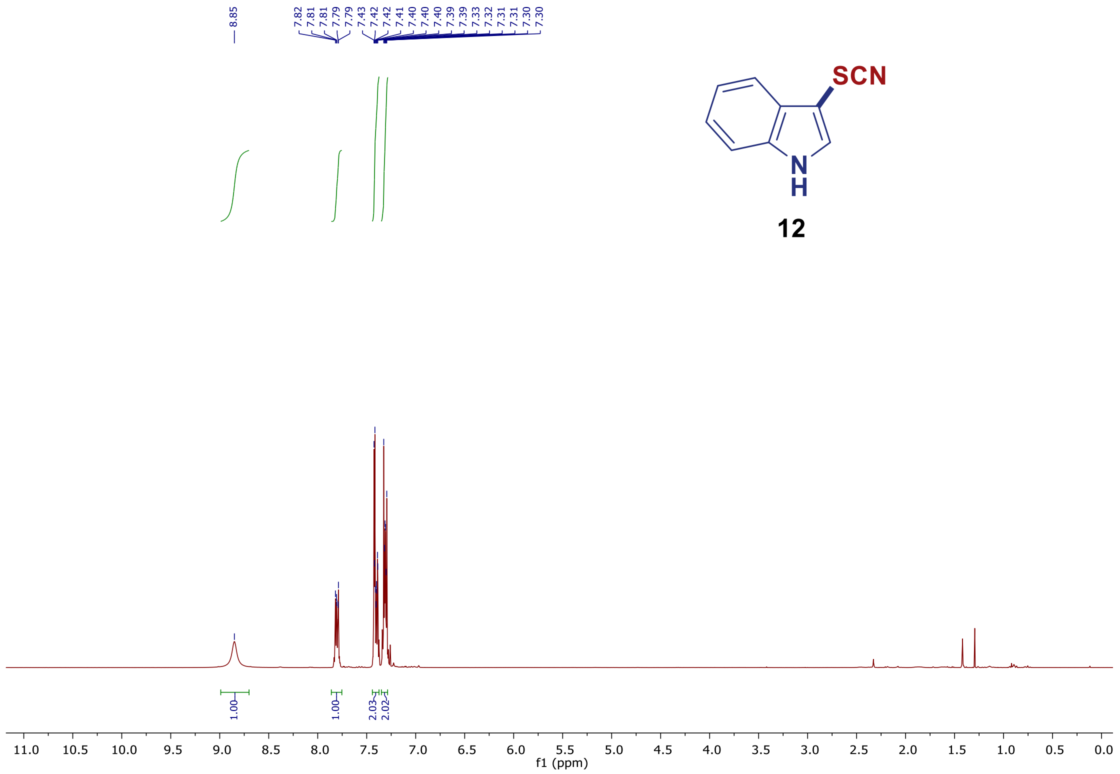

12

^{13}C-NMR (75 MHz, $CDCl_3$)

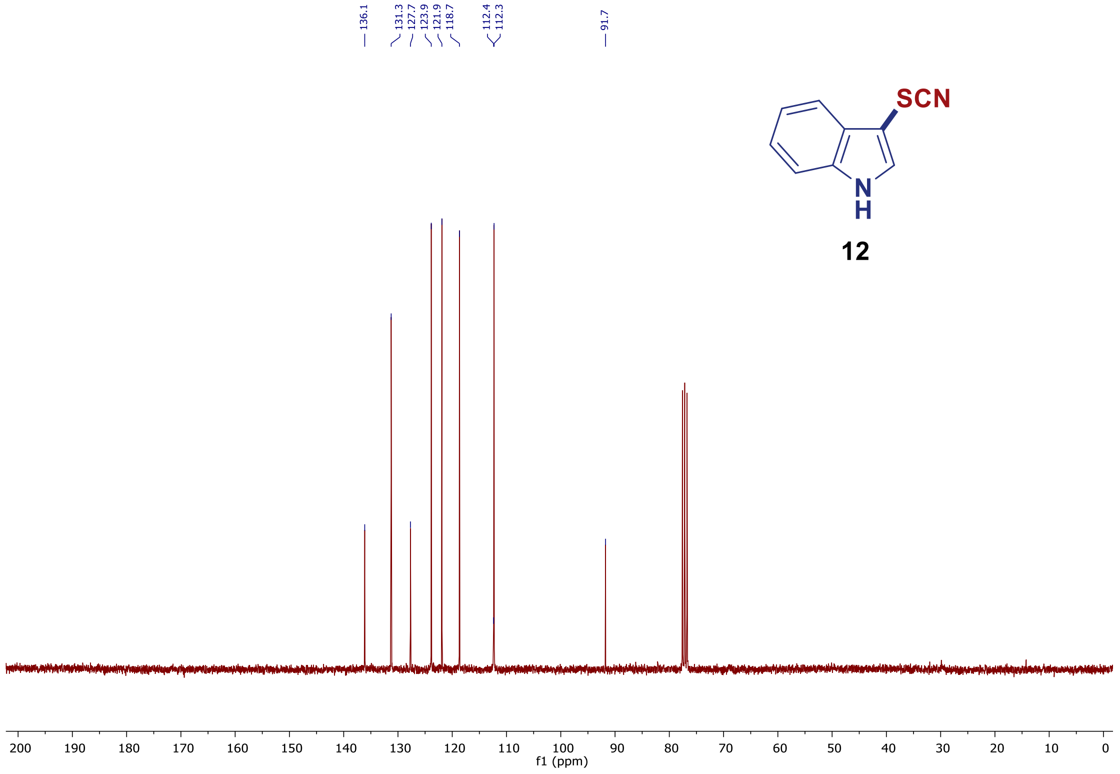

12

^{1}H-NMR (300 MHz, $CDCl_3$)

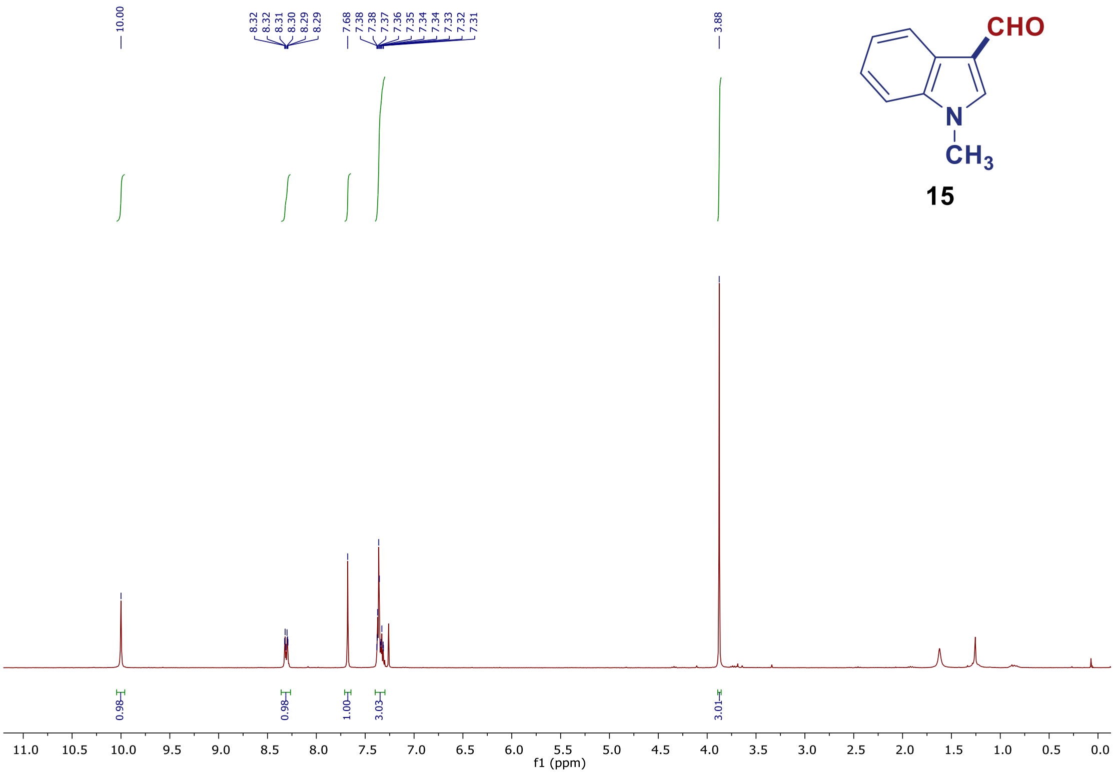

^{13}C-NMR (75 MHz, $CDCl_3$)

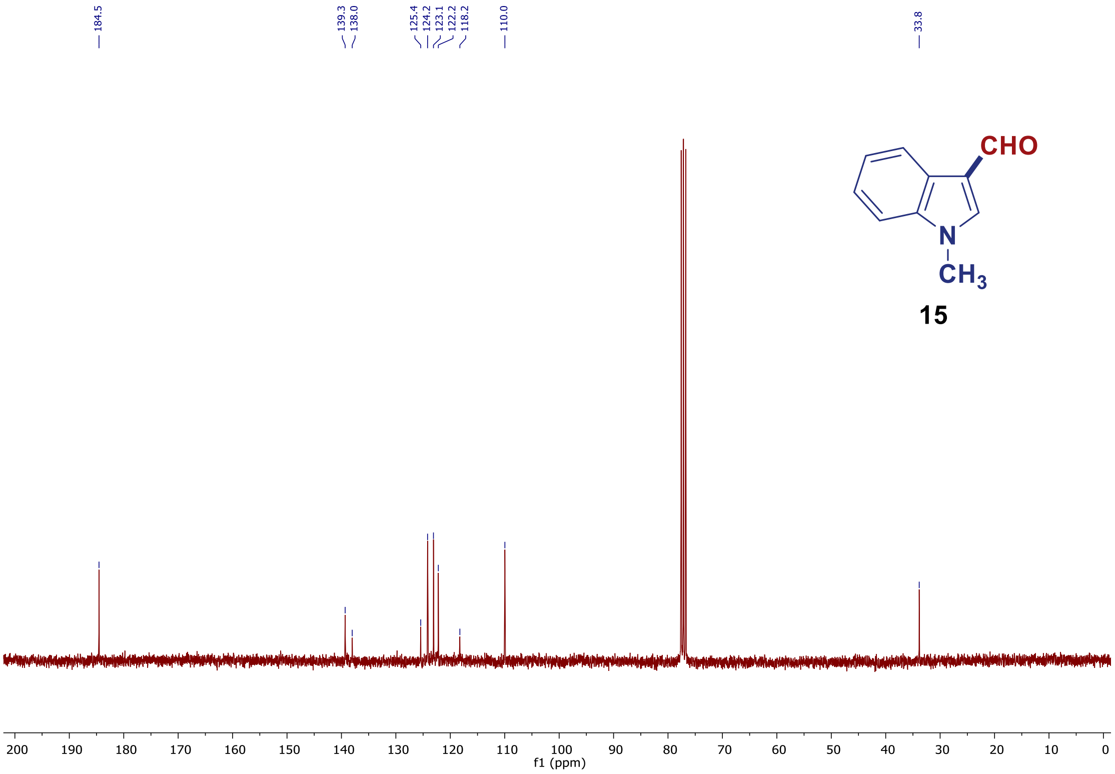

^{1}H-NMR (300 MHz, $CDCl_3$)

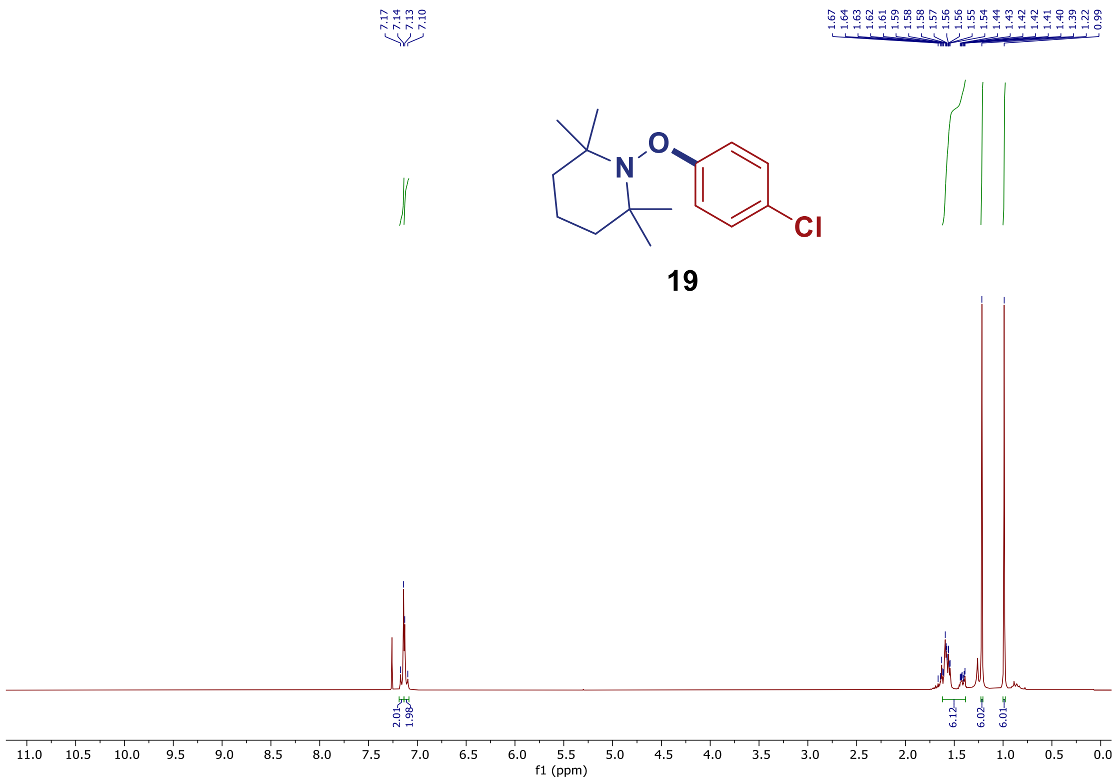

^{13}C-NMR (75 MHz, $CDCl_3$)

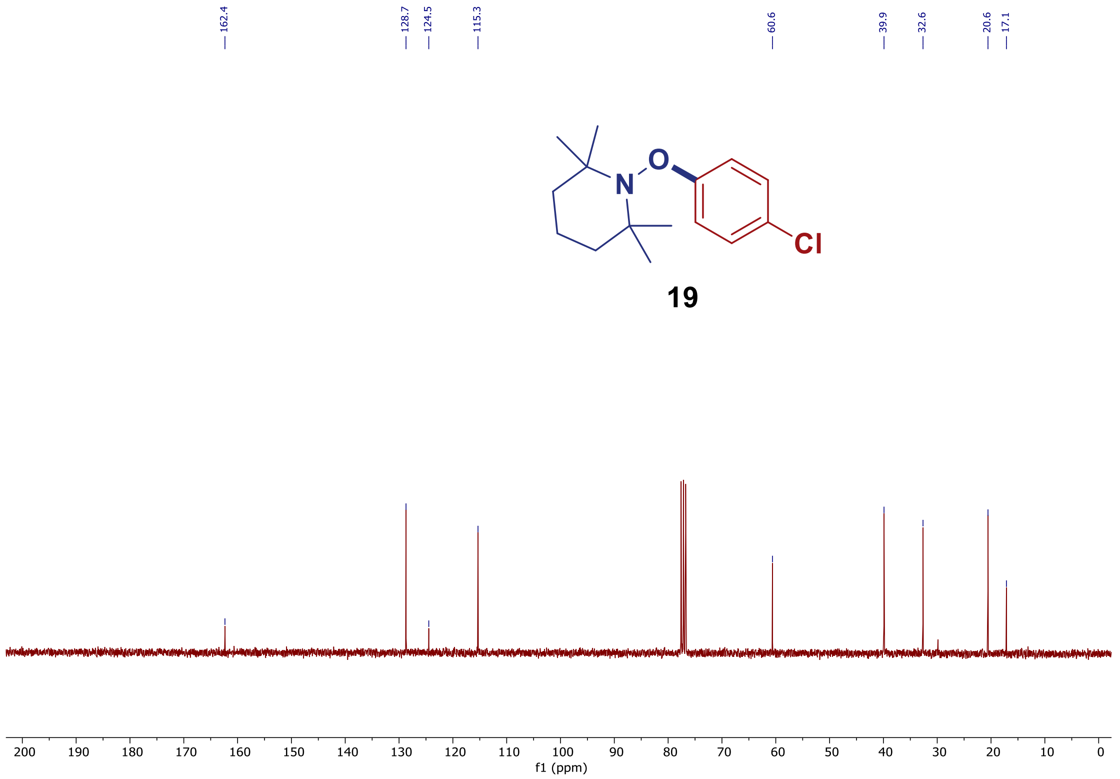

Supplement: Supplementary file 1 [file cs5c02173_si_001.pdf]
